# An umbrella review of effectiveness and efficacy trials for app-based health interventions

**DOI:** 10.1038/s41746-023-00981-x

**Published:** 2023-12-16

**Authors:** Sherry On Ki Chong, Sara Pedron, Nancy Abdelmalak, Michael Laxy, Anna-Janina Stephan

**Affiliations:** Professorship of Public Health and Prevention, TUM School of Medicine and Health, Technical University of Munich, Munich, Germany

**Keywords:** Outcomes research, Public health

## Abstract

Health interventions based on mobile phone or tablet applications (apps) are promising tools to help patients manage their conditions more effectively. Evidence from randomized controlled trials (RCTs) on efficacy and effectiveness of such interventions is increasingly available. This umbrella review aimed at mapping and narratively summarizing published systematic reviews on efficacy and effectiveness of mobile app-based health interventions within patient populations. We followed a pre-specified publicly available protocol. Systematic reviews were searched in two databases from inception until August 28, 2023. Reviews that included RCTs evaluating integrated or stand-alone health app interventions in patient populations with regard to efficacy/effectiveness were considered eligible. Information on indications, outcomes, app characteristics, efficacy/effectiveness results and authors’ conclusions was extracted. Methodological quality was assessed using the AMSTAR2 tool. We identified 48 systematic reviews published between 2013 and 2023 (35 with meta-analyses) that met our inclusion criteria. Eleven reviews included a broad spectrum of conditions, thirteen focused on diabetes, five on anxiety and/or depression, and others on various other indications. Reported outcomes ranged from medication adherence to laboratory, anthropometric and functional parameters, symptom scores and quality of life. Fourty-one reviews concluded that health apps may be effective in improving health outcomes. We rated one review as moderate quality. Here we report that the synthesized evidence on health app effectiveness varies largely between indications. Future RCTs should consider reporting behavioral (process) outcomes and measures of healthcare resource utilization to provide deeper insights on mechanisms that make health apps effective, and further elucidate their impact on healthcare systems.

## Introduction

Ageing populations, rising prevalence in treatment-intensive and costly non-communicable diseases and increasing shortage of specialized personnel pose serious threats to the financial sustainability of healthcare systems^1^. Without timely transformations of healthcare systems, rising socioeconomic and geographical inequalities in disease burden and unmet patient needs may be further exacerbated by inequalities in access to adequate healthcare services^2^.

Over the last decade, advancements in mobile technology have created new opportunities to meet this challenge. Most notably, mobile- or tablet-based health applications (apps) gained attention for their potentially beneficial effect on patients’ lives. For example, the use of mobile health (mhealth) apps can activate patients with chronic conditions to engage in online education, peer support, lifestyle monitoring and coaching consultations and help track their health status, fostering self-engagement and self-compliance in the disease management process to improve health outcomes^3^. Additionally, mhealth apps can bridge geographical barriers for access to healthcare, offering real-time reaction to patient needs in remote locations^4^. Lastly, health apps can relieve the burden on medical personnel by supporting medication prescription management and intake, as well as symptoms monitoring^5^.

The importance of new technologies has also been highlighted in the World Health Organization (WHO) global strategy on digital health 2020–2025^6^. Member countries are encouraged to develop digital healthcare strategies considering national contexts such as culture, public needs and available resources. However, large-scale integration of new technologies into standard care processes requires sufficient confidence in their effectiveness and cost-efficiency. Effectiveness can for example be hampered through technological challenges faced by users and delivery agents, data protection issues or privacy concerns, use of ineffective components and suboptimal sustainability in user engagement or long-term effects^7^. Also, population-wide implementation may, in some instances, add to existing health inequalities in society by introducing a digital divide^8,9^ with regard to access to (first), usage of (second), and benefits from usage (third) of digital health technology^10^. Large-scale implementation would, in such cases, entail the waste or misallocation of (usually scarce) healthcare system resources and, in the worst case, pose the risk of detrimental individual health effects, and loss of trust in technology or the healthcare system. A corroborated respective evidence base is therefore a prerequisite for health systems to initiate adequate policy reforms.

Mirroring the increase in available health apps, also the number of scientific evaluations of their efficacy and effectiveness has increased over the last decade. To make these research results actionable, an up-to-date, comprehensive yet concise mapping of the available high-quality evidence on efficacy and effectiveness of mhealth apps is required. A previous umbrella review attempted to summarize systematic reviews on a broad spectrum of telemedicine interventions beyond mhealth apps with heterogenous study designs including non-randomized controlled trials and various disease indications^11^. However, this broad summation of different intervention technologies and evidence levels makes it hard to draw conclusions specifically for mhealth apps. A second existent mapping of effectiveness reviews on mhealth interventions focused specifically on diabetes indications, but also included systematic reviews on heterogeneous study designs and various types of mhealth interventions beyond mhealth apps^12^. Another umbrella review focused on randomized controlled trials (RCTs) and restricted its scope to diabetes, dyslipidemia and hypertension, while trying to summarize evidence not only on mhealth apps, but for a broader range of telemedicine interventions^13^. Thus, there is still a currently unmet need to identify both well-researched and potentially under-researched indications with regard specifically to mhealth app effectiveness.

The objective of this umbrella review is to systematically map and summarize existing systematic reviews of RCTs investigating the effectiveness of mobile phone or tablet app-based mhealth interventions in patients. We provide a summary of investigated patient populations, the specific intervention configurations and features, reported comparators, outcomes used to assess efficacy and effectiveness, and assess overall review quality, whereas synthesizing or even re-analysing outcome data is beyond the scope of this study.

## Results

### Study selection

**Fig. 1:**
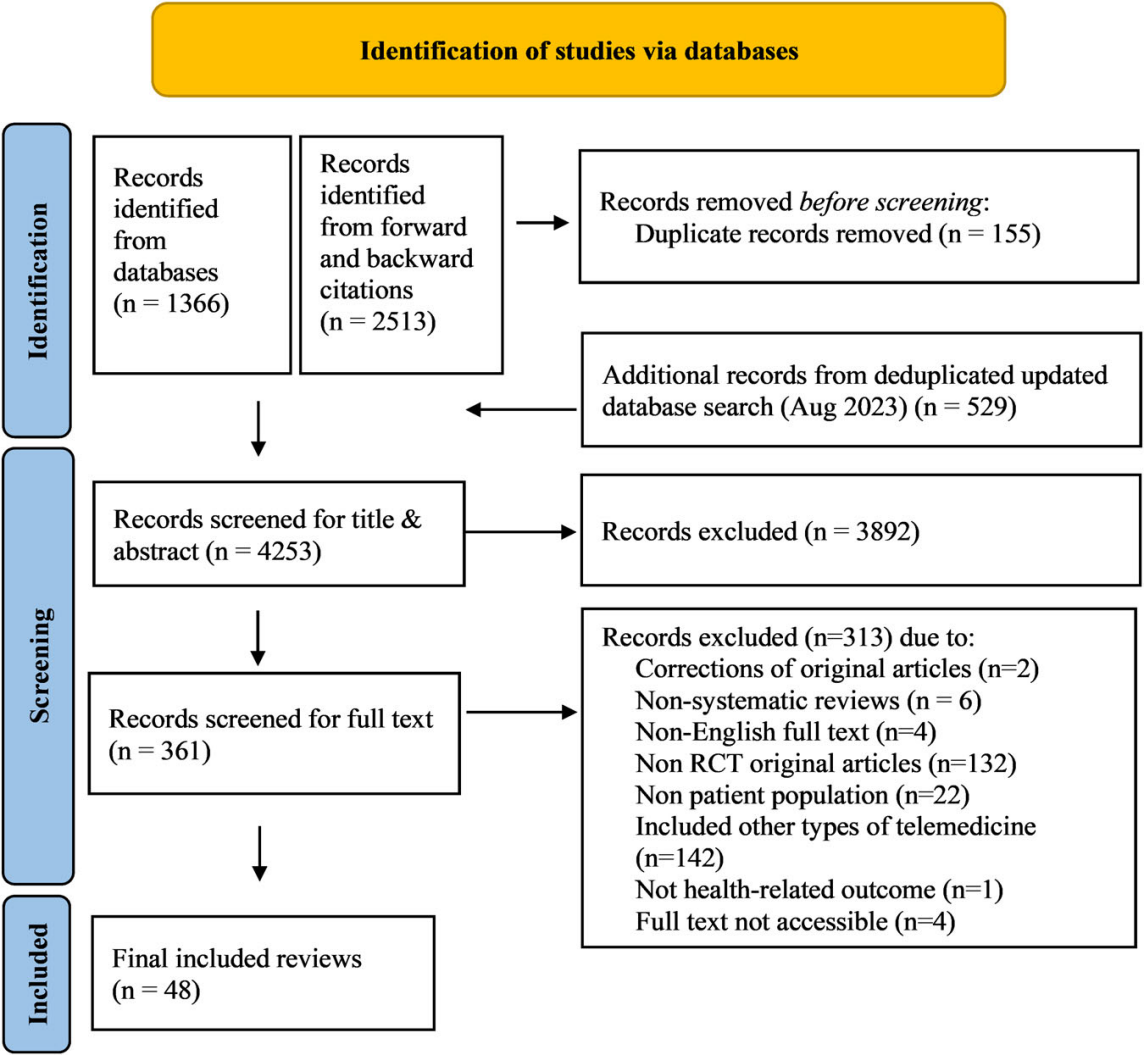
PRISMA flow chart of retrieved, screened and included articles. Flow chart illustrating the process of study identification for the present umbrella review with database searches (last updated on August 28, 2023), deduplication, title and abstract screening as well as full-text screening, leading to a final inclusion decision for *n* = 48 systematic reviews.

The study selection process according to PRISMA requirements^14^ is summarized in Fig. 1. The database search yielded a total of 1895 records, with additional 2513 records identified through forward and backward citation searching of records from the initial search deemed eligible after full text screening by the first author. After de-duplication, 4253 articles were screened by title and abstract. Of these, 3892 records were excluded, and 361 records were included for full text screening. The final number of included articles was 48. Inter-rater reliability (IRR) for title-/abstract screening and full-text screening was κ = 0.3469 and κ = 0.9326, respectively. A list of the 313 studies excluded after full-text screening with exclusion reasons for each study can be found in Supplementary Table 1.

### Review characteristics

**Fig. 2:**
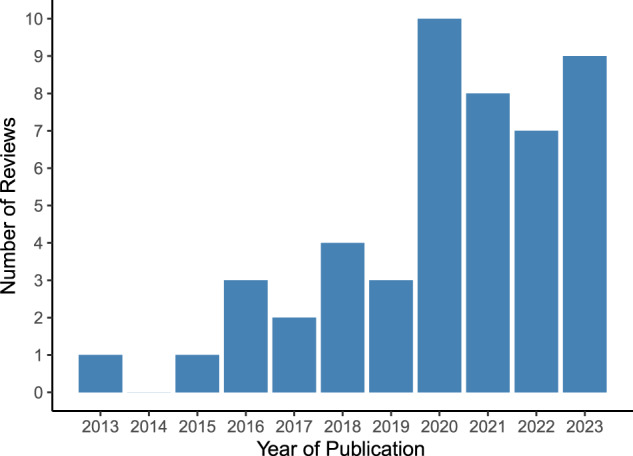
Number of included reviews by publication year. Vertical bar chart illustrating the number of included systematic reviews (*n* = 48 in total) on the y-axis stratified by year of publication on the x-axis.

Included reviews were published between 2013 and 2023, with the highest number of reviews published in 2020 (*n* = 10) and the first three quarters of 2023 (*n* = 9) (see Fig. 2).

All included reviews considered articles without geographic restrictions, except one focusing on China^15^. The number of RCT studies included in a review ranged from two to 36. Out of the 48 included reviews, 35^15–49^ conducted data pooling and meta-analyses whereas 13 reviews^50–62^ provided a narrative synthesis without meta-analysis. Median follow-up periods ranged from 1 to 10 months, with no respective information reported in six reviews^15,31–33,50,53^. A summary of review characteristics is shown in Supplementary Table 2.

### Methodological quality

**Fig. 3:**
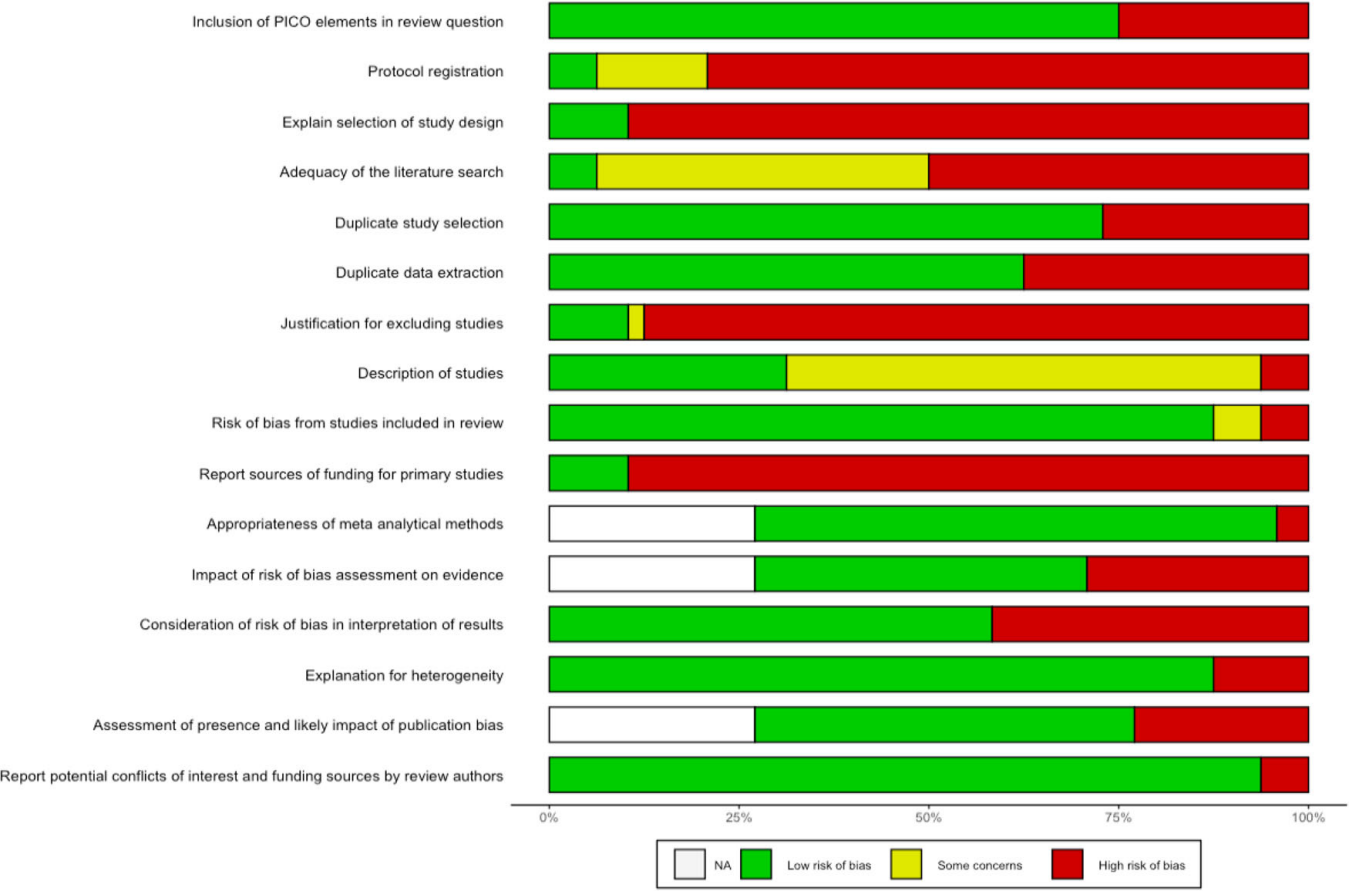
Frequency of risk of bias for each domain. Horizontal stacked bar chart illustrating on the x-axis the share of the *n* = 48 (100%) included systematic reviews which was rated as either low risk of bias (green), showing some concerns with regard to bias (yellow) or high risk of bias (red), for each of the 16 AMSTAR items (listed on the y-axis), respectively. White bar stacks represent the share of systematic reviews without meta-analysis, to which AMSTAR2 items 11, 12, and 15 were not applicable (“NA”). The acronym PICO in the first AMSTAR2 item stands for Population, Intervention, Comparator, Outcome.

Figure 3 summarizes the frequency of each AMSTAR2 rating for each domain across reviews. Supplementary Fig. 1 additionally presents the domain-specific methodological quality ratings for each review.

Sixteen reviews stated that they had registered or otherwise published a review protocol^17,19,25,34,35,38,40–42,47,48,51,54,56,60,62^. After checking these protocols, thirteen were rated as incomplete as they missed information on the search terms defining the search strategy (item 2)^17,19,34,38,40,41,47,48,51,54,56,60,62^. All reviews searched at least two databases and provided their full search strategy in the final report, but 25 reviews^16,17,19,21,22,27–29,33,38,39,41,43,45,47–50,52–55,58,59,61^ failed to justify publication restrictions, for example regarding language, entailing a “no” on item 4. Six reviews provided a list of studies excluded at full-text screening stage (item 7)^26,37,42,48,56,57^. Overall, a satisfactory assessment tool for risk of bias was used (item 9). Three reviews reported conflicts of interest (item 16)^16,42,49^. We rated one review as moderate quality^56^. IRR for quality assessment across all items and reviews was κ = 0.6671. Item-specific IRRs can be found in Supplementary Table 3.

### Extraction results

Included RCTs covered populations from all continents, with a majority of studies conducted in high- or middle-income countries such as the United States, China, Australia, United Kingdom, Spain, Norway and Japan. Seven reviews^33,38,45,46,48–50^ did not report countries of included studies.

**Fig. 4:**
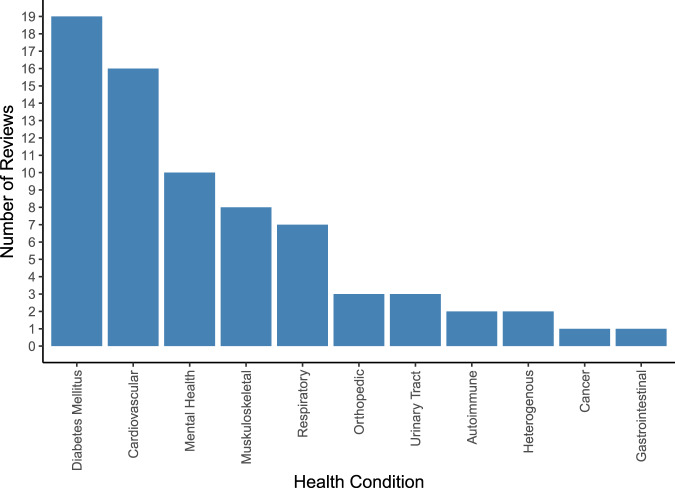
Frequency of aggregated disease indications addressed in the included systematic reviews. Vertical bar chart illustrating the number of included systematic reviews (on the y-axis) covering each of the 11 aggregated groups of health conditions (on the x-axis) which we identified across the *n* = 48 included systematic reviews. The total number of systematic reviews included in the graph exceeds the number of included systematic reviews as some systematic reviews cover more than one group of health conditions. Cardiovascular conditions include hypertension, stroke, obesity, atrial fibrillation, heart failure, myocardial infarction, coronary heart disease, hypercholesterolemia, prediabetes and cardiovascular disease. Diabetes mellitus includes type 2 diabetes, type 1 diabetes, diabetes, and gestational diabetes. Musculoskeletal conditions include fibromyalgic syndrome, musculoskeletal disorders, chronic pelvic pain, chronic musculoskeletal pain, multiple sclerosis, chronic low back pain, chronic neck pain, non-specific lower back pain, unspecified chronic pain, chronic pain or fibromyalgia, Parkinson, and neurological disorders. Mental health conditions include depression, anxiety, bipolar disorder, autism, post-traumatic stress disorder, attention deficit hyperactivity disorder, and schizophrenia. Respiratory conditions include asthma, chronic obstructive pulmonary disease, lung transplant, allergic rhinitis, and chronic lung disease. Autoimmune conditions include autoimmune deficiency syndrome and psoriasis. Orthopedic conditions include osteoarthritis, spina bifida, and post-operative knee aristoplasty. Urinary Tract Disorders include urinary incontinence and interstitial cystitis. Heterogenous diseases include unspecified chronic diseases and multimorbidity. Cancer includes chemotherapy related to cancer toxicity. Gastrointestinal conditions include irritable bowel syndrome. For a more detailed illustration of frequencies for all 49 ungrouped individual health conditions, see Supplementary Fig. 2.

An overview of covered health indications is displayed in Supplementary Fig. 2 and, in more aggregated disease groups, Fig. 4. Most reviews targeted specific indications, including type 2 diabetes (T2DM) (*n* = 5)^19,20,22,23,26^, hypertension (*n* = 4)^15,27,31,38^, depression (*n* = 3)^33,53,61^, overweight/obesity (*n* = 3)^40,41,52^, chronic obstructive pulmonary disease (COPD) (*n* = 2)^35,39^, urinary incontinence (*n* = 2)^56,62^, asthma (*n* = 1)^57^, autism spectrum disorders (*n* = 1)^32^, post-traumatic stress disorder (PTSD) (*n* = 1)^59^, type 1 diabetes (*n* = 1)^47^, Parkinson’s disease (*n* = 1)^45^, knee arthroplasty (*n* = 1)^46^ and lower back pain (*n* = 1)^51^. Twenty-two reviews covered multiple conditions within their scope, such as diabetes of various types (*n* = 7)^18,21,24,25,36,37,50^, chronic non-communicable diseases (*n* = 2)^55,58^, anxiety and depression (*n* = 2)^43,49^, conditions requiring rehabilitation (*n* = 2)^42,44^, pediatric diseases (*n* = 1)^54^, diseases requiring medication (*n* = 2)^17,34^, cardiovascular diseases (*n* = 2)^16,30^, pain conditions (*n* = 2)^48,60^, mental illnesses (*n* = 1)^28^, or a combination of diabetes and hypertension (*n* = 1)^29^.

Information on pooled sample size was provided by all except three reviews^31,45,46^ and ranged from 282 to 7669 patients. Further information on extracted population characteristics can be found in Supplementary Tables 4 and 5.

The health apps performed a wide array of functions including symptoms monitoring and assessments, medication reminders, real-time biofeedback, personalized programs and education, tailor-made motivational messages or cues and feedback, social support, communication with healthcare professionals, goal setting, data storage, and visualization.

A summary of reported app characteristics and functionalities is documented in Supplementary Table 4.

Comparator conditions were described in 43 out of the 48 reviews. Some reviews included usual care comparators only, others varied between usual care or other control apps, to lighter technological features, text messages, paper-based monitoring diaries, in-person and standard education, and no treatment. A summary of reported comparators is shown in Supplementary Table 4.

Eleven reviews reported results on T2DM patients. Five focused on T2DM alone^19,20,22,23,26^, while six included broader populations but conducted (subgroup) analyses specifically on T2DM^21,24,25,36,37,58^. All eleven reviews except one^19^ assessed glycemic control, operationalized as change in glycated hemoglobin (HbA1c) reduction, as main or secondary outcome. Further outcomes comprised changes in body weight, waist circumference or body mass index^19,20,22,23^, fat mass or percentage of body fat^19^, lipids, blood pressure, lifestyle changes, medication use^20,22,23^, psychological symptoms and quality of life (QoL)^23^. All studies that focused on other types of diabetes (e.g., type 1 diabetes, mixed types, prediabetes, gestational diabetes)^18,36,37,47,50^ focused on HbA1c changes as main outcome, while only a few included adverse events^37,54^ and QoL^54^. Another outcome reported for diabetic populations was medication adherence, but it was reported in samples that did not exclusively include diabetes patients (patients with prescription drugs^16^, chronic disease patients^34,54^).

Reviews including patients with hypertension focused on evaluating the impact of health app interventions on medication adherence^27,31,38^, systolic and diastolic blood pressure^15,27,38^, and health behaviors^27,38^. Three reviews^16,17,34^ reported on medication adherence, and two reviews^16,29^ on systolic and diastolic blood pressure, lipids and anthropometric outcomes in samples that did not exclusively include hypertensive patients.

Reviews focusing on patients with depression measured improvements of depressive symptoms^33,53,61^, and self-esteem and QoL^53,61^. Two reviews additionally reported results for medication adherence^17,61^, one^61^ on psychiatric admissions, medication adherence and side effects, resilience, attitudes, sleep disturbances and further psychological and behavioral outcomes^61^. Further reviews reported on depressive^28,43,49^, mania and psychotic symptoms as well as adverse events^28^ and anxiety symptoms^43,49^ in samples that did include depression patients, however not exclusively. Outcomes evaluated in other mental health indications included symptoms related to PTSD^59^, positive and negative psychotic symptoms including hallucinations or delusions and absence of experience (in schizophrenia), mania and depression symptoms (bipolar disorder)^28^, and autism-related outcomes based on the Mullen Scales of Early Learning, MacCarthur-Bates Communication Development Inventory and Communication and Symbolic Behavior Scales^32^.

Reviews focusing on overweight and obesity used the following outcomes: weight loss^40,41,52^, waist circumference, blood pressure, lipids, HbA1c, energy intake^40,41^, physical activity, body fat, BMI^40^, motivation and adherence^52^.

Outcomes reported in other indications can be found in Supplementary Tables 4 and 6.

**Fig. 5:**
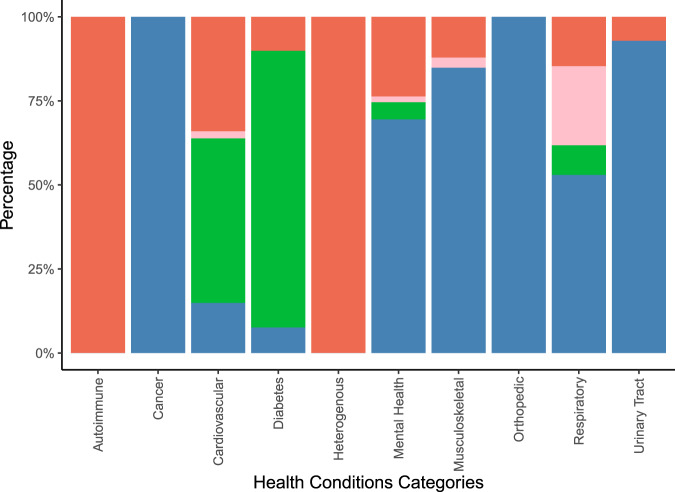
Distribution of outcome types reported by categorized disease indications. Vertical stacked bar chart illustrating the percentage of behavioral (red stacks), healthcare resource utilization (rose stacks), laboratory/anthropometric (green stacks), and patient reported (blue stacks) outcomes on the y-axis by aggregated groups of health conditions (on the x-axis) covered in the total of *n* = 48 included systematic reviews. Behavioral outcomes comprised behaviors such as medication adherence and physical activity. Healthcare resource utilization comprised outcomes such as hospitalizations, and doctor visits. Laboratory/anthropometric outcomes included clinical or body measurements. Patient-reported outcomes comprised subjectively reported outcomes such as quality of life or symptom improvement.

Figure 5 illustrates the types of outcomes reported in the systematic reviews by aggregated groups of investigated health conditions. More details on the uncategorized outcomes can be found in Supplementary Tables 4 and 6.

Twenty-three out of 35 meta-analyses conducted subgroup analyses^18–21,23–29,33,34,36,37,40,41,43,47–49,53,57^. Investigated subgroups were defined by number, types and intensities of app features, differentiation between standalone or integrated interventions, baseline demographic or disease-related participant characteristics, follow-up duration, intervention duration, study quality, type of comparator, sample size, attrition, analytic approaches, and outcome assessment methods. Summaries of the subgroups investigated are in Supplementary Table 7.

Overall, 41 out of the 48 reviews concluded that app-based health interventions were effective in improving health outcomes. The seven systematic reviews which did not conclude that app-based health interventions were effective reported inconclusive results as some studies showed effectiveness and others did not^35,51,53,54,57,61^, or reported clinically irrelevant improvements^41^. Reported synthesized outcomes, types of effect estimates, and number of underlying individual studies were heterogeneous. A complete overview of extracted results and summaries of author’s conclusions is shown in Supplementary Table 6. For example, for medication adherence, meta-analysed effect estimates reported in 6 systematic reviews ranged between 0.38 and 0.8 standardized mean difference, with 2−14 studies summarized, 6 out of 6 meta-analysed point estimates suggesting an increase in medication adherence, and 6 out of 6 meta-analytic results suggesting statistically significant effects. Three reviews additionally expressed effect estimates for medication adherence in terms of Odds Ratios or mean differences. For HbA1c, meta-analysed effect estimates from 13 systematic reviews ranged between 0.06% and −0.6% (weighted) mean difference, with 2−24 studies summarized, 27 out of 28 meta-analysed point estimates suggesting a reduction in % HbA1c, and 18 out of 28 meta-analytic results suggesting statistically significant effects. For systolic blood pressure (SBP), meta-analysed effect estimates from 9 systematic reviews ranged between 0.1 and −8.12 mmHg (weighted) mean difference, with 2−13 studies summarized, 8 out of 10 meta-analysed point estimates suggesting a reduction in SBP, and 4 out of 10 meta-analytic results suggesting statistically significant effects. Two reviews additionally expressed effect estimates for SBP in terms of Odds Ratios or mean differences. In two reviews with meta-analysed results on SBP the outcome unit was unclear.

## Discussion

Despite a rapid increase in evidence syntheses from RCTs on health app effectiveness, availability of systematic reviews varies widely between indications. By far, the most frequently covered indication is T2DM (confirming a trend that was already emerging in 2018 according to a previous umbrella review with a similar scope^63^), followed by hypertension, obesity and depression, potentially leaving evidence gaps for other diseases. A substantial proportion of systematic reviews attempted to cover multiple indications at the same time or had no disease focus or outcome restrictions, entailing less punctuated result interpretations and more frequent narrative syntheses without meta-analysis instead of quantitative syntheses due to the resulting large heterogeneity across included studies.

Generally, as also criticized in the abovementioned 2018 umbrella review^63^, average follow-up times remained short. Also, outcome measures varied between and within indications, ranging from more objective and proximal laboratory parameters such as HbA1c and blood pressure to more subjective patient-reported measures such as symptom scores and QoL. In contrast, measures of healthcare resource utilization such as frequency of physician visits or hospitalizations, were rarely reported. This leaves knowledge gaps as to how health apps affect health outcomes, how long their effects are sustained and the resulting burden for healthcare systems in terms of healthcare utilization effects.

Interestingly, effectiveness was evaluated with different degrees of standardization across indications (e.g., involving standardized cut-offs and objectively measurable outcomes). For the most frequently summarized cardiometabolic disease indications (i.e., diabetes, obesity and hypertension), we noted a more consistent and standardized use of outcome measures compared to other indications. Measurements of HbA1c levels in T2DM, blood pressure levels in hypertension, weight in obesity and medication adherence independent of indication^17,25,38^ were very frequently used. In contrast, symptom severity was overall measured in a much more heterogeneous fashion, as illustrated by a review which reported results on seven different measures for depressive symptoms alone^33^. This heterogeneity is partially expected given the nature of different conditions but makes comparability and pooling of evidence across studies more difficult.

In addition, a high proportion of the reviews mainly summarized evidence from high and higher-middle income countries, a phenomenon that did seem to be driven by a skewed availability of primary RCTs rather than selection bias introduced at the level of the systematic reviews (for example through narrowly defined inclusion criteria). This lack of research in low and lower-middle income countries is surprising and not proportional to the large potential for improving healthcare access in underserved communities usually credited to app-based interventions^64^. In these settings, healthcare staff is especially scarce, while smartphones are already widely available^64^. Consequently, more attention should be paid to these settings, especially to investigate how mhealth apps can help overcome barriers encountered to access healthcare.

While we extracted both effect estimates and authors' conclusions from all systematic reviews, with 41 out of the 48 reviews concluding that app-based health interventions may be effective and the other seven stating inconclusive results, we would like to point out not only that a large share of systematic reviews was rated to be of low quality, but also that interpretation should ideally be indication- and outcome specific, and take not just statistical significance, but also clinical relevance and timing of outcome measurement into account. The broad range of effect sizes and the effect heterogeneity within and across reviews included in this umbrella review underlines that, like other complex interventions, mhealth apps can have multidimensional effects as they can simultaneously target multiple patient-relevant outcome parameters and succeed in positively affecting some of these, but not others. We believe that this umbrella review can provide helpful and easily accessible orientation for policy leaders, clinicians and patients to find relevant synthesized evidence (and an assessment of its quality). However, weighing the potential benefits of mhealth app use against the resource investments that are required for their implementation, will necessarily remain a context-specific task.

Even though we did not extract the effect estimates from pooled stratified and subgroup analyses conducted by included systematic reviews, we extracted the type of the conducted subgroup analyses, which we believe provides already interesting insights about which factors researchers seem to perceive as impacting mhealth apps’ effectiveness in improving patients’ health outcomes. Our results suggest that specific app characteristics and features, such as data tracking and patient feedback modalities^24,28^, were seen as decisive for effectiveness. This is in line with previous findings: health tracking features and enhanced proximal engagement with the health app through push notifications with tailored health messages are essential in motivating users for self-management of their chronic diseases, and thereby enhance effectiveness of mhealth apps^65,66^.

Additionally, specific design aspects of the intervention trials were often investigated as potential contributors to heterogeneity in measured effects. Specifically, the choice of comparators^33,49^, duration of the app-based intervention and follow-up^23,36,47^, and integration of mhealth apps with other supplementary intervention strategies were often treated as stratification factors for additional analyses. It is known that comparator choice can affect effect sizes and success of blinding to study group assignment^67^. Subgroup analyses by intervention/follow-up duration may help identify waning efficacy over time, for example if participants lose motivation after having used a specific app for a period of time to track their health status, or in case of dissatisfaction with app features^68^. Frequently used cut-offs for differentiation of follow-up were three to six months compared to longer term duration (e.g., up to 12 months), indicating a relatively narrow focus on short- and mid-run effects. Subgroup meta-analyses by socio-economic characteristics of study participants, or by availability of alternative healthcare access routes (e.g., rural/urban setting), were rarely conducted, indicating that equity aspects may currently not be adequately considered in effectiveness and efficacy trials of health apps, despite ongoing discussions about the potential of digital health interventions to entail positive and negative equity effects (digital divide^69^).

To further increase the systematic and evidence-based evaluation of mhealth app interventions, more comprehensive and consistent reporting of app functionalities and selection of outcome measures is needed. For example, application developers and researchers could follow international guidelines such as the WHO’s mHealth evidence reporting and assessment checklist^70^. This checklist could be a useful tool in further standardizing reporting as it captures different essential domains from intervention delivery to replicability. Furthermore, future research could follow our comprehensive search strategy to map other parts (e.g., with regards to population base or study design) of the existing evidence base for effectiveness of mobile phone applications. Additionally, it might be an interesting next step to pool effect estimates across systematic reviews after deduplicating the pool of underlying individual studies. Additional extractions and syntheses of results from pooled stratified/subgroup analyses may further elucidate factors which drive app effectiveness.

To maximize efficacy and minimize harms of mhealth apps, the social determinants of successful app-based health interventions should be analyzed. In fact, previous effectiveness reviews did not investigate if different population subgroups (e.g., different age groups, genders, socio-economic status) benefit equally and equitably from app-based health interventions. Individuals who lack technological or digital literacy might engage less with or benefit less from such health interventions^71^ potentially aggravating existing inequities.

In spite of the remaining open questions outlined above, given their potential effectiveness in improving health behaviors and health outcomes, fostering diffusion of mhealth apps into health care systems to support patients in getting actively involved in their own disease management process, bridge geographical barriers in healthcare and relieve detrimental consequences of medical personnel shortages might be worthwhile.

The strength of this umbrella review is in its scope which was to map and characterize the growing volume of systematic reviews on randomized effectiveness trials of mhealth apps in patients, highlighting the skewed amount of scientific evidence for different indications and providing a concise overview of reported outcomes and the most frequently conducted subgroup analyses, stressing the importance of specific app features for effectiveness.

Further strengths of this umbrella review are the publicly available pre-specified protocol and the systematic process of summarizing the available evidence. This provides a transparent and replicable approach for future research and potentially regular updates in this fast-moving field. Our systematic search strategy with well-defined terms and criteria according to the PRISMA guidelines in two large and widely used databases enhanced the replicability of the results.

Nevertheless, several limitations should be considered when interpreting our findings. First, only published articles written in English were included because we considered it improbable that authors would decide on publishing the results of an extensive endeavor such as the conduct of a systematic review in a language other than English, as this would considerably restrict the potential for impact and uptake of the results. Nevertheless, systematic reviews written in other languages may have been missed. Even though we deemed this risk relatively low we cannot fully exclude that this decision may have contributed to the lop-sided distribution of countries represented in the included systematic reviews. Similarly, although we searched the most important systematic reviews database with the Cochrane Database of Systematic Reviews (CDSR), the most comprehensive international biomedical citation database (MEDLINE)^72^, and the high-recency database PubMedCentral, we cannot fully exclude that searches of additional (for example, more regionally focused) databases may have identified some additionally relevant reviews. However, since our objective was not so much to summarize or even re-analyze outcome data (which requires strict completeness of the evidence base), we deemed the residual risk of missing out on a minority of available systematic reviews reconcilable with the primary purpose of mapping and characterizing the growing volume of systematic reviews on randomized effectiveness trials of mhealth apps in patients through this umbrella review. Also, some of the primary RCTs may have been included in more than one of the systematic reviews which constituted the evidence base for this umbrella review. An interesting next step might be to conduct meta-analyses for specific indications and outcomes after deduplication of the underlying original empirical studies, taking into account additional heterogeneities at individual study level, such as those in terms of follow-up time and RCT quality. While beyond the mapping scope of this umbrella review, such synthesis attempts at deduplicated individual RCT level can lead to interpretable non-biased effect estimates and avoid attribution of undue weight to the results of those RCTs that were of low quality or included in multiple systematic reviews^73^, thereby providing additional insights.

We acknowledge that by focusing on effectiveness outcomes from systematic reviews of RCTs within patient groups only, this umbrella review only reflects a specific part of the available evidence on smartphone applications, since other evidence dimensions except effectiveness/efficacy (e.g., equity, cost effectiveness) were not considered. Furthermore, we did not include systematic reviews that comprised studies on the general population, other observational study designs that may allow for causal inference, or other intervention study types, such as non-randomized trials. Quasi-experimental studies could have also been an interesting source of evidence which we did not include. The rationale for our rather strict inclusion criteria with regard to study design was twofold: First, we wanted to specifically include systematic reviews with the highest internal validity when it comes to causal inference which could be undermined with lack of randomization^74^. Second, we attempted to keep the basis for this umbrella review manageable and at the same time as homogenous as possible. We acknowledge that the downside of these decisions is that some relevant studies may have been excluded from this umbrella review.

Lastly, there were several systematic reviews that we excluded because they marginally failed to meet our inclusion criteria. Examples include a large systematic review by Cucciniello et al.^75^ which included 69 studies from chronic disease indications, however at least one study used WhatsApp as the intervention instead of a full-blown health app. Another example is a systematic review by Widdison et al.^76^ which summarized three RCTs on health app effectiveness in urinary incontinence, but additionally included one observational follow-up study of a previous RCT intervention arm. Although these studies potentially summarized evidence that could have been relevant to our research question, they were excluded from our umbrella review.

In conclusion, we found 48 systematic reviews published since 2013 that narratively or quantitatively synthesized effectiveness results for app-based health interventions in patients. These reviews targeted a range of different health conditions, with diabetes and hypertension being the most intensely covered and evaluated. There was substantial heterogeneity of what was defined as primary outcomes, but the majority of reviews concluded that app-based health interventions are likely to be effective. In reviews focusing on diabetes, obesity and hypertension, variability in reported outcome measures was lowest. Future research in other indications might follow these examples and attempt higher standardization of measurements, easing quantitative inference and allowing for more actionable conclusions. Additionally, studies with longer follow-up periods are required. Furthermore, the heterogenous methodological quality of the evidence included in this umbrella review highlights the need to take quality assessments into account for policy decisions. Lastly, future evidence synthesis attempts should also map the additional evidence provided by systematic reviews summarizing other study designs and general population instead of diseased populations. This would provide a definitive and full picture of the effectiveness of health app-based interventions and would support evidence-based public health and healthcare policy decisions alleviating economic pressures on healthcare systems.

## Methods

### Study design

We conducted an umbrella review of existing systematic reviews following (where applicable) the Preferred Reporting Items for Systematic Reviews and Meta-Analysis guidelines (PRISMA) checklist^14,77^ (Supplementary Table 8) as recommended elsewhere^78^. We uploaded a pre-specified review protocol to the Open Science Framework database prior to conducting the initial literature search^79^. For protocol development, we consulted guidance documents^78^ including those published by Cochrane^71^ and JBI^80^. The scope of the review, as well as the pre-defined search strategy, eligibility criteria and extraction targets outlined below remained essentially unchanged throughout the conduct of the review.

### Eligibility criteria

We defined eligibility criteria around types of studies, population, interventions, outcomes, and study language (Table 1).

**Table 1:** Eligibility criteria.

	Inclusion criteria	Exclusion criteria
Publication type	Systematic reviews (with or without meta-analysis)	Protocols of reviews, scoping reviews, umbrella reviews, secondary studies, primary studies, meta-analyses in contexts other than systematic reviews
Study designs targeted by the systematic review	Systematic reviews including only randomized controlled trials (including randomized controlled feasibility and pilot trials and different forms of randomization, such as cluster-level or stepped wedge)	Systematic reviews including nonrandomized controlled trials, cross-sectional studies, case-control studies, cohort studies, case reports
Language of the systematic review	Full text in English	Full text written in non-English languages
Populations targeted by the systematic review	Individuals with a diagnosed or self-reported disease or health issue (a) for which there is an ICD-10 code and (b) that is targeted by the respective app	General population without known disease or health issues, sub-analyses on specific patient groups within a review targeting the general population, populations with addictive health behaviors (tobacco use, drinking, gambling, substance use), pregnant women without additional medical conditions, users of apps aiming to support primary prevention/induce preventative health behavior changes in the general population, diagnose/screen for undetected conditions or assist health-care personnel in care delivery
Interventions targeted by the systematic review	Smartphone or tablet apps aiming to improve specific health related outcomes, app is a standalone or complementary intervention tool	Based only on other non-app technologies e.g., text messaging, wearable devices and websites, delivery or online consultation via apps not developed for health purposes
Outcomes targeted by the systematic review	Changes in health or care process outcomes at follow-up, any outcomes pertaining to the efficacy / effectiveness dimension of evidence	Outcomes evaluating solely dimensions of evidence other than efficacy / effectiveness

This umbrella review included systematic reviews with and without meta-analyses. Being the gold standard for efficacy evaluations, we included only systematic reviews of RCTs. Systematic reviews that did not include RCTs or that included RCTs together with other study types were excluded.

We considered only reviews that included efficacy/effectiveness trials of app-based health interventions in *patients*. The study participants had to have a specific disease or health issue (as defined by the International Classification of Diseases, 10th Revision [ICD-10])^81^, that was targeted by the intervention in question. Health issues could be diagnosed by a health professional or self-reported. In contrast, reviews that considered studies on general populations without any specific health problems were excluded, even if they reported sub-analyses on separate patient groups. Furthermore, we excluded reviews targeting individuals with potentially addictive behaviors such as tobacco use, drinking, gambling or other substance use, as these behaviors are classified in Chapter XXI as “factors influencing health status and contact with health services”, and not within the disease-related ICD-10 chapters, and we deemed a potential separate umbrella review for such behavioral factors more appropriate than a combination with clear-cut diseases. Similarly, reviews on pregnant women without any additional specific medical condition were also excluded, as ICD-10 does not classify normal pregnancy within the disease-related chapters but in Chapter XXI.

We included reviews focusing on interventions which aimed at improving specific health-related outcomes via smartphone or tablet apps. The app could be a standalone or complementary intervention tool (i.e., coupled with personal interactions, text messaging or social media). In contrast, reviews comprising studies that evaluated *solely* non-app technologies such as text messaging, social media, wearable devices or websites were excluded. Reviews comprising studies that *solely* involved online communication applications (e.g., Instagram, WhatsApp, WeChat, Telegram, Skype) were excluded unless the app was specifically designed for health or medical purposes. Reviews comprising studies that evaluated health apps aiming to support users in primary prevention or the process of (self-)diagnosis and/or (self-)screening for yet undetected conditions were excluded.

No restrictions were set with regard to the types of comparators.

Reviews reporting on health or care process outcomes pertaining to the efficacy or effectiveness dimension of evidence were included. These outcomes included, but were not limited to health outcomes, medication adherence, chronic disease management, or symptoms relief. Reviews reporting exclusively on other dimensions of evidence such as diagnostic accuracy, concordance, feasibility, cost-effectiveness, resource consumption, costs, equity, or measurement accuracy were excluded.

Only articles with available full text in English were considered, as we assumed the efficiency gains of implementing a language restriction to outweigh the risk of missing out on important evidence.

### Databases and search strategy

We searched MEDLINE and PubMedCentral via PubMed and the CDSR. Articles were included from database inception until March 15, 2022 and the search was updated on August 28, 2023.

The search strategy combined keywords and Medical Subject Headings (MeSH) structured around three components: (i) intervention; (ii) study design; (iii) outcome dimension (see Supplementary Tables 9 and 10 for the complete search strategy and number of associated hits in each electronic database).

Forward and backward citation searches were additionally conducted for articles deemed eligible after initial full text screening. Forward citation searches were conducted until August 8, 2022 using PubMed and Scopus.

### Selection process

After search completion and deduplication, two authors (SOKC and NA) carried out independent title and abstract screening according to the predefined eligibility criteria using the online software Rayyan^82^. Diverging decisions were resolved unanimously after discussion with up to two additional authors (SP and AJS).

After title/abstract screening, we assessed full texts of all potentially eligible articles against the eligibility criteria^57^. Whenever an inclusion criterion was not met, we stopped the screening of the respective full text and excluded the systematic review. One author (SOKC) conducted the full text screening of all systematic reviews. A second author (NA) independently double-checked all exclusion decisions. Diverging decisions were resolved unanimously with up to two additional authors (AJS and SP) included in the discussion.

### Data collection/extraction process and data items

One author (SOKC or NA) extracted data from all eligible articles after full-text screening using a predefined and pretested extraction form^79^. A second reviewer (NA or AJS) double-checked the extracted data. Conflicts were resolved unanimously, where necessary after discussion with a third reviewer (AJS). We extracted the following information from the included reviews: general information about the review (e.g., publication date, number of included studies), pooled population characteristics, app characteristics, comparators, outcomes, subgroup analyses, authors’ narrative conclusions on overall efficacy/effectiveness of app-based interventions.

### Methodological quality assessment

We used the Assessing the Methodological Quality of Systematic Reviews (AMSTAR2, see Supplementary Note 1) tool to evaluate methodological quality of all included systematic reviews^83^. AMSTAR2 covers 16 domains, of which seven are considered critical. Critical domains are deemed especially influential for review validity and include protocol pre-registration (item 2), literature search strategy (item 4), list and justification for excluded studies (item 7), risk of bias assessment (item 9), meta-analytical methods (item 11), consideration of risk of bias in results interpretation (item 13) and assessment of presence and likely impact of publication bias (item 15).

Each included review was rated for adequacy on each domain as either “Yes”, “No”, or “Partial Yes” (available only for domains 2, 4, 7, 8, and 9). For those articles that did not conduct meta-analyses, items 11, 12 and 15 were rated “Not Applicable”. Fulfillment of each dimension across the different reviews was illustrated using a table and heat map. Based on these domains, we also assigned a summary quality rating as “critically low” ( ≥ 2 “no” ratings on critical domains), “low” ( ≤ 1 “no” ratings on critical domains), “moderate” ( ≥ 2 “no” ratings on non-critical domains) or “high” ( ≤ 1 “no” on a non-critical domain) to each review. Quality appraisals were conducted in duplicate by two review authors (SOKC and AJS) and diverging ratings were resolved through discussion.

### Inter-rater reliability

IRR was calculated using Cohen’s Kappa (κ) for title- and abstract screening, full-text screening and the methodological quality assessment (overall and item specific).

### Data synthesis

We provide narrative summaries and graphical representations of publication years, population characteristics, type of underlying condition, type of intervention and type of outcomes assessed. As data from the same RCT may have contributed to the pooled effect estimates of more than one included systematic review, and due to the high expected heterogeneity of diseases and outcomes covered in the systematic reviews, a meta-analysis pooling systematic review results was not planned nor performed.

### Supplementary information

Supplementary material

## Data Availability

All the data presented and analyzed in this study is available in the paper and in the Supplementary Information File.
