# Supplementary material for: An umbrella review of effectiveness and efficacy trials for app-based health interventions
Source: NPJ Digit Med. 2023 Dec 16;6:233. doi: 10.1038/s41746-023-00981-x (PMC10725431; doi:10.1038/s41746-023-00981-x)
Supplement: Supplementary file 1 — Supplementary material [file 41746_2023_981_MOESM1_ESM.pdf]

Supplementary information to “An umbrella review of effectiveness and efficacy trials for app-based health interventions”

Sherry On Ki Chong[1], Sara Pedron[1], Nancy Abdelmalak[1], Michael Laxy[1], Anna-Janina Stephan[1]

[1]Technical University of Munich, Germany; TUM School of Medicine and Health, Professorship of Public Health and Prevention

Supplementary Information File

Table of Contents

Supplementary Figure 1 Domain specific methodological quality ratings 2

Supplementary Figure 2 Frequency of ungrouped individual health conditions addressed in the included systematic reviews 4

Supplementary Table 1. List of excluded full texts with reasons 6

Supplementary Table 2. General review characteristics of 48 included reviews on the effectiveness of app-based health interventions 45

Supplementary Table 3. Interrater-Reliability for Quality Appraisal 47

Supplementary Table 4. Overview of included populations, interventions, comparators, and outcomes of 48 included reviews on the effectiveness of app-based health interventions 48

Supplementary Table 5. Details on review population characteristics of 48 included reviews on the effectiveness of app-based health interventions 69

Supplementary Table 6. Summary of outcomes, effect estimates and conclusions of 48 included reviews on the effectiveness of app-based health interventions 72

Supplementary Table 7. Summary of Subgroup Analyses 92

Supplementary Table 8. PRISMA Checklist 94

Supplementary Table 9. Search Strategy and Hits in PubMed 98

Supplementary Table 10. Search Strategy in the Cochrane Database of Systematic Reviews 100

Supplementary Note 1. 102

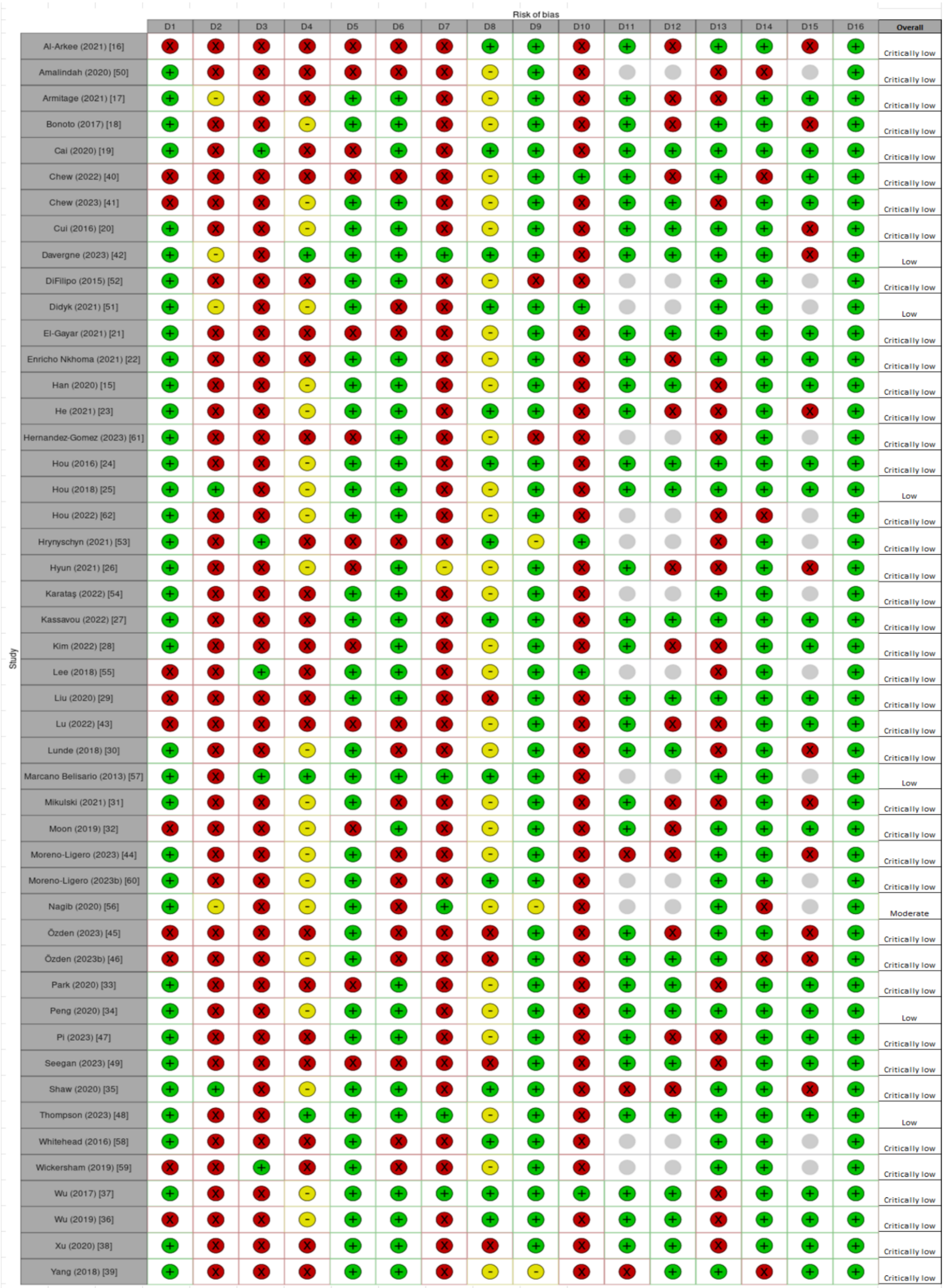

Supplementary Figure 1 Domain specific methodological quality ratings

Heatmap illustrating, for each of the 16 AMSTAR2 items (columns labelled D1-D16) and each of the n=48 included systematic reviews (listed row-wise in the first column), the item-specific quality rating, with a "plus" sign in a green circle indicating low risk of bias, a dash in a yellow circle indicating unclear risk of bias, an "X" in a red circle indicating high risk of bias, and a grey circle indicates that the respective AMSTAR2 item (D11, D12, D15) is not applicable to the respective systematic review (as no meta-analysis was conducted). Reference numbers in square brackets after the author year indication correspond to the reference numbering in the main manuscript text. Item D1 refers to inclusion of PICO ("Population, Intervention, Comparator, Outcome) elements in the review question. Item D2 refers to protocol registration. Item D3 refers to explanation of selection of included study designs. Item D4 refers to adequacy of the literature search. Item D5 refers to duplication of study selection. Item D6 refers to duplication of data extraction. Item D7 refers to justifications for excluding studies. Item D8 refers to description of studies. Item D9 refers to risk of bias from studies included in the review. Item D10 refers to reporting of funding sources for primary studies. Item D11 refers to appropriateness of meta-analytic methods. Item D12 refers to impact of the risk of bias on evidence. Item D13 refers to consideration f risk of bias in interpretation of results. Item D14 refers to explanation of heterogeneity. Item D15 refers to assessment of presence and likely impact of publication bias. Item D16 refers to reporting of potential conflict of interest and funding sources by review authors. The overall quality rating in the last column is based on the classification as so-called "critical domains" for items 2, 4, 7, 9, 11, 13, and 15. It was derived as "critically low" for ≥2 "no" ratings on critical domains, "low" for ≤1 "no" ratings on critical domains, "moderate" for ≥2 "no" ratings on non-critical domains or "high" for ≤1 "no" on a non-critical domain). Figure created with the help of: McGuinness, LA, Higgins, JPT. Risk-of-bias VISualization (robvis): An R package and Shiny web app for visualizing risk-of-bias assessments. Res Syn Meth. 2020; 1- 7. https://doi.org/10.1002/jrsm.1411.

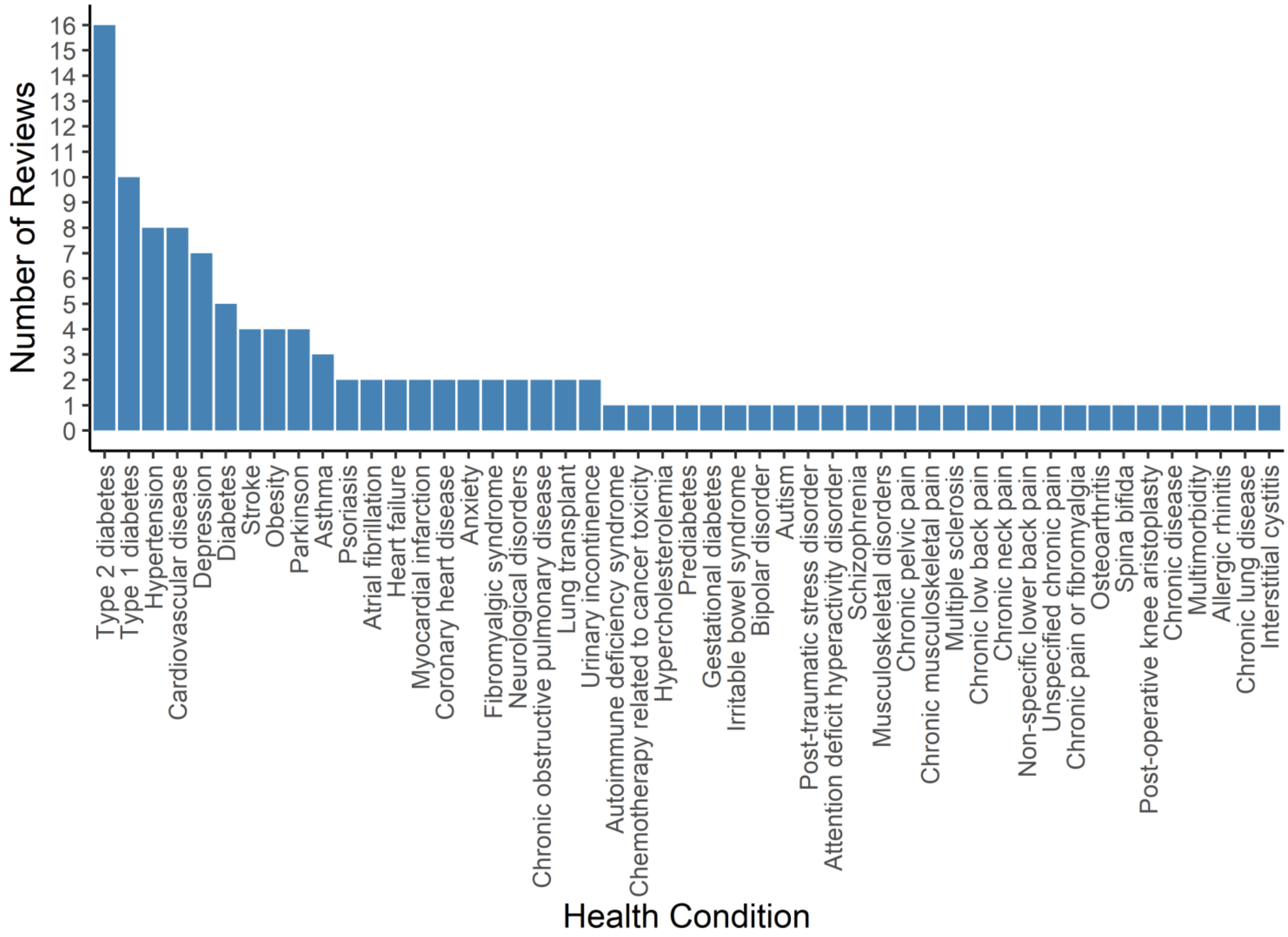

Supplementary Figure 2 Frequency of ungrouped individual health conditions addressed in the included systematic reviews

Vertical bar chart illustrating the number of included systematic reviews (out of n=48 in total) on the y-axis covering the 49 ungrouped individual health conditions which we identified across these systematic reviews, respectively. The total number of systematic reviews included in the graph exceeds the number of included systematic reviews as some reviews cover more than one health condition.

Supplementary Table 1. List of excluded full texts with reasons

First Author	Year	Article Title	Exclusion Reason
Abasi et al.	2021	Effectiveness of mobile health-based self-management application for posttransplant cares: A systematic review	Not RCT
Abdulhussein et al.	2022	A systematic review of the current availability of mobile applications in eyecare practices	Not RCT
Adesina et al.	2021	Effectiveness and Usability of Digital Tools to Support Dietary Self-Management of Gestational Diabetes Mellitus: A Systematic Review	Not RCT + not app-based intervention
Aji et al.	2021	Framework for the Design Engineering and Clinical Implementation and Evaluation of mHealth Apps for Sleep Disturbance: Systematic Review	Not RCT
Akbari et al.	2019	The effects of mobile health interventions on lipid profiles among patients with metabolic syndrome and related disorders: A systematic review and meta-analysis of randomized controlled trials	Not app-based intervention
Akingbade et al.	2023	Effect of mHealth interventions on psychological issues experienced by women undergoing chemotherapy for breast cancer: A systematic review and meta-analysis	Not app-based intervention
Alhussein et al.	2022	Digital Health Technologies for Long-term Self-management of Osteoporosis: Systematic Review and Meta-analysis	Not RCT

Allida et al.	2020	mHealth education interventions in heart failure	Not app-based intervention
Allman-Farinelli et al.	2020	The efficacy of electronic health interventions targeting improved sleep for achieving prevention of weight gain in adolescents and young to middle-aged adults: A systematic review	Not RCT + not app-based intervention
Almasi et al.	2020	Mobile health technology for hypertension management: A systematic review	Not RCT + not app-based intervention
Almeida et al.	2020	Methodological Quality of Manuscripts Reporting on the Usability of Mobile Applications for Pain Assessment and Management: A Systematic Review	Not RCT
Alwashmi et al.	2016	The Effect of Smartphone Interventions on Patients with Chronic Obstructive Pulmonary Disease Exacerbations: A Systematic Review and Meta-Analysis	Not RCT + not app-based intervention
Alzahrani et al.	2022	The Adoption and Acceptance of mHealth Interventions for Self-Management of Hypertension Among Adult Patients: A Systematic Review	Not app-based intervention
Aminuddin et al.	2021	Effectiveness of smartphone-based self-management interventions on self-efficacy, self-care activities, health-related quality of life and clinical outcomes in patients with type 2 diabetes: A systematic review and meta-analysis	Not app-based intervention
Anmella et al.	2022	Smartphone-based interventions in bipolar disorder: Systematic review and meta-analyses of efficacy. A position paper from	Not RCT

		the International Society for Bipolar Disorders (ISBD) Big Data Task Force	
Antosik-Wójcińska et al.	2020	Smartphone as a monitoring tool for bipolar disorder: a systematic review including data analysis, machine learning algorithms and predictive modelling	Not RCT
Antoun et al.	2022	The Effectiveness of Combining Nonmobile Interventions With the Use of Smartphone Apps With Various Features for Weight Loss: Systematic Review and Meta-analysis	Not RCT + population not diseased
Armitage et al.	2017	A systematic review of the literature with met-analysis and meta-regression: the use of smartphone applications to support medication adherence.	Not systematic review
Avcı et al.	2023	The Effect of Mobile Phone-Based Interventions on Blood Pressure in Stroke Patients: A Systematic Review of Randomized Controlled Trials	Not app-based intervention
Bacigalupo et al.	2013	Interventions employing mobile technology for overweight and obesity: an early systematic review of randomized controlled trials	Not app-based intervention
Bahadori et al.	2020	A review of current use of commercial wearable technology and smartphone apps with application in monitoring individuals following total hip replacement surgery	Not RCT + not app-based intervention
Baniasadi et al.	2020	Surgical Patients Follow-Up by Smartphone-Based Applications: A Systematic Literature Review	Not RCT

Barengo et al.	2022	Mobile Health Technology in the Primary Prevention of Type 2 Diabetes: a Systematic Review	Not app-based intervention
Baron et al.	2012	The impact of mobile monitoring technologies on glycosylated hemoglobin in diabetes: a systematic review	Not RCT + not app-based intervention
Bassi et al.	2021	Efficacy of eHealth Interventions for Adults with Diabetes: A Systematic Review and Meta-Analysis	Not app-based intervention
Bateman et al.	2017	Categorizing Health Outcomes and Efficacy of mHealth Apps for Persons With Cognitive Impairment: A Systematic Review	Not app-based intervention
Baxter et al.	2021	Effectiveness of mobile health interventions to improve nasal corticosteroid adherence in allergic rhinitis: A systematic review	Not app-based intervention
Bellei et al.	2018	Diabetes Mellitus m-Health Applications: A Systematic Review of Features and Fundamentals	Not RCT + not app-based intervention + no effectiveness outcome
Bennett et al.	2014	Electronic health (eHealth) interventions for weight management among racial/ethnic minority adults: a systematic review	Not app-based intervention
Bond et al.	2021	Systematic Review of RCTs Assessing the Effectiveness of mHealth Interventions to Improve Statin Medication Adherence: Using the Behaviour-Change Technique Taxonomy to Identify the Techniques That Improve Adherence	Not app-based intervention

Bonet et al.	2017	Use of mobile technologies in patients with psychosis: A systematic review	Not English
Bonnechère et al.	2021	Is mHealth a Useful Tool for Self-Assessment and Rehabilitation of People with Multiple Sclerosis? A Systematic Review	Not app-based intervention
Brannon et al.	2015	A systematic review: is there an app for that? Translational science of pediatric behavior change for physical activity and dietary interventions	Population not diseased
Buettner et al.	2020	Self-Management of Diabetes Mellitus Patients Using mHealth Applications: A Systematic Review	Not RCT
Buettner et al.	2010	Evidence supporting technology-based interventions for people with early-stage Alzheimer's disease	Not RCT + not app-based intervention
Butler et al.	2020	Usability of eHealth and Mobile Health Interventions by Young People Living With Juvenile Idiopathic Arthritis: Systematic Review	Not app-based intervention
Buttazzoni et al.	2021	Smartphone-Based Interventions and Internalizing Disorders in Youth: Systematic Review and Meta-analysis	Not RCT
Camacho et al.	2019	Smartphone Apps to Support Coordinated Specialty Care for Prodromal and Early Course Schizophrenia Disorders: Systematic Review	Not RCT
Cavero-Redondo et al.	2020	Effect of Behavioral Weight Management Interventions Using Lifestyle mHealth Self-Monitoring on Weight Loss: A Systematic Review and Meta-Analysis	Not RCT

Chan et al.	2017	Review of Use and Integration of Mobile Apps Into Psychiatric Treatments	Not systematic review + not RCT + population not diseased + not app-based intervention + no effectiveness outcome
Chandan et al.	2021	Multimedia based education on bowel preparation improves adenoma detection rate: Systematic review & meta-analysis of randomized controlled trials	Not app-based intervention
Changizi et al.	2017	Effectiveness of the mHealth technology in improvement of healthy behaviors in an elderly population-a systematic review	Not app-based intervention
Chaplais et al.	2015	Smartphone Interventions for Weight Treatment and Behavioral Change in Pediatric Obesity: A Systematic Review	Not app-based intervention
Chen et al.	2016	The Effect of Information Communication Technology Interventions on Reducing Social Isolation in the Elderly: A Systematic Review	Not app-based intervention
Cheng et al.	2020	Technology-Delivered Psychotherapeutic Interventions in Improving Depressive Symptoms Among People with HIV/AIDS: A Systematic Review and Meta-analysis of Randomised Controlled Trials	Not app-based intervention
Cheng et al.	2017	Effectiveness of Interactive Self-Management Interventions in Individuals With Poorly Controlled Type 2 Diabetes: A Meta-Analysis of Randomized Controlled Trials	Not app-based intervention
Chin-Jung et al.	2021	Effects of mobile health interventions on improving glycemic stability and quality of	Not RCT

		life in patients with type 1 diabetes: A meta-analysis	
Chivilgina et al.	2020	mHealth for schizophrenia spectrum disorders management: A systematic review	Not RCT
Cho et al.	2017	Technology-Mediated Interventions and Quality of Life for Persons Living with HIV/AIDS. A Systematic Review	Not app-based intervention
Choi et al.	2021	Efficacy of mobile health care in patients undergoing fixed orthodontic treatment: A systematic review	Not app-based intervention
Choi et al.	2020	Mobile-Application-Based Interventions for Patients With Hypertension and Ischemic Heart Disease: A Systematic Review	Not RCT
Choi et al.	2019	mHealth technologies for osteoarthritis self-management and treatment: A systematic review	Not app-based intervention
Choi et al.	2020	A systematic review of mobile health technologies to support self-management of concurrent diabetes and hypertension	Not RCT
Chong et al.	2023	Effects of mobile apps intervention on medication adherence and type 2 diabetes mellitus control: A systematic review and meta-analysis	Full text not accessible
Colombo et al.	2019	Current State and Future Directions of Technology-Based Ecological Momentary Assessment and Intervention for Major Depressive Disorder: A Systematic Review	Not RCT + not app-based intervention + no effectiveness outcome
Coorey et al.	2018	Effectiveness, acceptability and usefulness of mobile applications for cardiovascular	Not RCT

		disease self-management: Systematic review with meta-synthesis of quantitative and qualitative data	
Cornet et al.	2018	Systematic review of smartphone-based passive sensing for health and wellbeing	Population not diseased
Cruz-Cobo et al.	2022	Effectiveness of mHealth Interventions in the Control of Lifestyle and Cardiovascular Risk Factors in Patients After a Coronary Event: Systematic Review and Meta-analysis	Not app-based intervention
Cucciniello et al. (excluded after data extraction stage)	2021	Development features and study characteristics of mobile health apps in the management of chronic conditions: a systematic review of randomised trials	Not app-based intervention
Dario et al.	2017	Effectiveness of telehealth-based interventions in the management of non-specific low back pain: a systematic review with meta-analysis	Not app-based intervention
Darling et al.	2017	Systematic Review and Meta-Analysis Examining the Effectiveness of Mobile Health Technologies in Using Self-Monitoring for Pediatric Weight Management	Not app-based intervention
David et al.	2022	The effect of mobile health focused on diet and lifestyle on blood pressure: a systematic review and Meta-analysis	Population not diseased + not app-based intervention
Davis et al.	2021	Gamified applications for secondary prevention in patients with high cardiovascular disease risk: A systematic review of effectiveness and acceptability	Not RCT

Dawes et al.	2021	Mobile health technology for remote home monitoring after surgery: a meta-analysis	Not RCT
de Araújo et al.	2021	Mobile apps for the treatment of diabetes patients: a systematic review	Not RCT + not app-based intervention + no effectiveness outcome
De La Cruz Monroy et al.	2019	The Use of Smartphone Applications (Apps) for Enhancing Communication With Surgical Patients: A Systematic Review of the Literature	Not RCT + population not diseased
Debon et al.	2019	Mobile health applications for chronic diseases: A systematic review of features for lifestyle improvement	Not RCT + no effectiveness outcome
Deng et al.	2017	Effects of mobile phone management applications for biological and biochemical parameters in patients with type 2 diabetes mellitus: A meta-analysis	Not English
Desai et al.	2019	Use of smartphone applications to improve quality of bowel preparation for colonoscopy: a systematic review and meta-analysis	Not RCT + not app-based intervention
Diano et al.	2023	A Systematic Review of Mobile Apps as an Adjunct to Psychological Interventions for Emotion Dysregulation	Not RCT
Ding et al.	2020	Effects of Different Telemonitoring Strategies on Chronic Heart Failure Care: Systematic Review and Subgroup Meta-Analysis	Not app-based intervention
Direito et al.	2017	mHealth Technologies to Influence Physical Activity and Sedentary Behaviors: Behavior Change Techniques, Systematic	Not app-based intervention

		Review and Meta-Analysis of Randomized Controlled Trials	
Domhadrt et al.	2021	Efficacy of digital health interventions in youth with chronic medical conditions: A meta-analysis	Not app-based intervention
Domhadrt et al.	2020	Are Internet- and mobile-based interventions effective in adults with diagnosed panic disorder and/or agoraphobia? A systematic review and meta-analysis	Not RCT + not app-based intervention
Domhardt et al.	2019	Internet- and mobile-based interventions for anxiety disorders: A meta-analytic review of intervention components	Not app-based intervention
Donker et al.	2013	Smartphones for smarter delivery of mental health programs: a systematic review	Not RCT
Dounavi et al.	2019	Mobile Health Applications in Weight Management: A Systematic Literature Review	Not RCT
Du et al.	2020	The efficacy of e-health in the self-management of chronic low back pain: A meta analysis	Not app-based intervention
Dubad et al.	2018	A systematic review of the psychometric properties, usability and clinical impacts of mobile mood-monitoring applications in young people	Not RCT
Dunham et al.	2021	Smartphone Applications Designed to Improve Older People's Chronic Pain Management: An Integrated Systematic Review	Not RCT
Dwiputra et al.	2023	Smartphone-Based Cardiac Rehabilitation Program Improves Functional Capacity in	Not app-based intervention

		Coronary Heart Disease Patients: A Systematic Review and Meta-Analysis	
El Bizri et al.	2021	Mobile health technologies supporting colonoscopy preparation: A systematic review and meta-analysis of randomized controlled trials	Not app-based intervention
El-Gayar et al.	2013	Mobile applications for diabetes self-management: status and potential	Not RCT
El-Haj-Mohamad et al.	2023	Smartphone-delivered mental health care interventions for refugees: A systematic review of the literature	Not RCT + population not diseased
Eberle et al.	2021	Effectivness of specific mobile health applications (mHealth-apps) in gestational diabtetes mellitus: a systematic review	Population not diseased
Elkefi et al.	2023	The role of digital health in supporting cancer patients' mental health and psychological well-being for a better quality of life: A systematic literature review	Not app-based intervention
Emberson et al.	2021	Effectiveness of Smartphone-Based Physical Activity Interventions on Individuals' Health Outcomes: A Systematic Review	Population not diseased
Enyioha et al.	2022	Effectiveness of Mobile Phone and Web-Based Interventions for Diabetes and Obesity Among African American and Hispanic Adults in the United States: Systematic Review	Not app-based intervention
Escobar-Viera et al.	2021	Feasibility of mHealth interventions for depressive symptoms in Latin America: a systematic review	Not RCT + not app-based intervention

Escriva Boulley et al.	2018	Digital health interventions to help living with cancer: A systematic review of participants' engagement and psychosocial effects	Not RCT
Fakih El Khoury et al.	2019	The Effects of Dietary Mobile Apps on Nutritional Outcomes in Adults with Chronic Diseases: A Systematic Review and Meta-Analysis	Not RCT
Fanning et al.	2012	Increasing physical activity with mobile devices: a meta-analysis	Not app-based intervention
Farzandipour et al.	2017	Patient Self-Management of Asthma Using Mobile Health Applications: A Systematic Review of the Functionalities and Effects	Not RCT
Feigerlova et al.	2020	E-health education interventions on HbA(1c) in patients with type 1 diabetes on intensive insulin therapy: A systematic review and meta-analysis of randomized controlled trials	Not app-based intervention
Fekete et al.	2021	Role of new digital technologies and telemedicine in pulmonary rehabilitation : Smart devices in the treatment of chronic respiratory diseases	Not app-based intervention
Feldman et al.	2021	A systematic review of mHealth application interventions for peripartum mood disorders: trends and evidence in academia and industry	Not RCT
Feng et al.	2021	Psychological or educational eHealth interventions on depression, anxiety or stress following preterm birth: a systematic review	Not RCT + not app-based intervention

Firth et al.	2017	The efficacy of smartphone-based mental health interventions for depressive symptoms: a meta-analysis of randomized controlled trials	Population not diseased
Firth et al.	2017	Can smartphone mental health interventions reduce symptoms of anxiety? A meta-analysis of randomized controlled trials	Not app-based intervention
Firth et al.	2015	Smartphone Apps for Schizophrenia: A Systematic Review	Not RCT
Flaucher et al.	2023	Evaluating the Effectiveness of Mobile Health in Breast Cancer Care: A Systematic Review	Not RCT
Flores Mateo et al.	2015	Mobile Phone Apps to Promote Weight Loss and Increase Physical Activity: A Systematic Review and Meta-Analysis	Not RCT
Fowler et al.	2021	Harnessing technological solutions for childhood obesity prevention and treatment: a systematic review and meta-analysis of current applications	Not app-based intervention
Furness et al.	2020	Impact of the Method of Delivering Electronic Health Behavior Change Interventions in Survivors of Cancer on Engagement, Health Behaviors, and Health Outcomes: Systematic Review and Meta-Analysis	Not app-based intervention
Gál et al.	2021	The efficacy of mindfulness meditation apps in enhancing users' well-being and mental health related outcomes: a meta-analysis of randomized controlled trials	Population not diseased

Gan et al.	2021	Effect of Engagement With Digital Interventions on Mental Health Outcomes: A Systematic Review and Meta-Analysis	Not app-based intervention
Gandapur et al.	2016	The role of mHealth for improving medication adherence in patients with cardiovascular disease: a systematic review	Not app-based intervention
Gandhi et al.	2017	Effect of Mobile Health Interventions on the Secondary Prevention of Cardiovascular Disease: Systematic Review and Meta-analysis	Not app-based intervention
Garabedian et al.	2015	Mobile Phone and Smartphone Technologies for Diabetes Care and Self-Management	Not systematic review + not RCT
Garg et al.	2022	Application of Mobile Technology for Disease and Treatment Monitoring of Gestational Diabetes Mellitus Among Pregnant Women: A Systematic Review	Not RCT
Garrido et al.	2019	What Works and What Doesn't Work? A Systematic Review of Digital Mental Health Interventions for Depression and Anxiety in Young People	Not app-based intervention
Gire et al.	2017	mHealth based interventions for the assessment and treatment of psychotic disorders: a systematic review	Not app-based intervention
Gong et al.	2023	Effectiveness of mHealth diet interventions in cancer survivors: A systematic review and meta-analysis of randomized controlled trials	Not app-based intervention
Gual-Montolio et al.	2020	How Are Information and Communication Technologies Supporting Routine Outcome	Not RCT

		Monitoring and Measurement-Based Care in Psychotherapy? A Systematic Review	
Hamilton et al.	2018	Smartphones in the secondary prevention of cardiovascular disease: a systematic review	Not RCT
Han et al.	2018	Effectiveness of Mobile Health Application Use to Improve Health Behavior Changes: A Systematic Review of Randomized Controlled Trials	Population not diseased
He et al.	2023	Conversational Agent Interventions for Mental Health Problems: Systematic Review and Meta-analysis of Randomized Controlled Trials	Not app-based intervention
Helsel et al.	2018	Telemedicine and Mobile Health Technology Are Effective in the Management of Digestive Diseases: A Systematic Review	Not app-based intervention
Holmen et al.	2017	Tailored Communication Within Mobile Apps for Diabetes Self-Management: A Systematic Review	Not RCT
Holtz et al.	2012	Diabetes management via mobile phones: a systematic review	Not RCT + population not diseased + not app-based intervention
Huang et al.	2021	Efficacy of Telemedicine for Patients with Chronic Wounds: A Meta-Analysis of Randomized Controlled Trials	Not app-based intervention
Huguet et al.	2016	A Systematic Review of Cognitive Behavioral Therapy and Behavioral Activation Apps for Depression	Not RCT
Hui et al.	2017	The use of mobile applications to support self-management for people with asthma: a	Not app-based intervention

		systematic review of controlled studies to identify features associated with clinical effectiveness and adherence	
Ilagan et al.	2020	Smartphone applications targeting borderline personality disorder symptoms: a systematic review and meta-analysis	Not RCT
Indraratna et al.	2020	Mobile Phone Technologies in the Management of Ischemic Heart Disease, Heart Failure, and Hypertension: Systematic Review and Meta-Analysis	Not app-based intervention
Iribarren et al.	2021	Effectiveness of Mobile Apps to Promote Health and Manage Disease: Systematic Review and Meta-analysis of Randomized Controlled Trials	Not RCT + population not diseased
Islam et al.	2020	Use of Mobile Phone App Interventions to Promote Weight Loss: Meta-Analysis	Not RCT + population not diseased
Jacoby	2019	Systematic Review of Mobile Phone Apps Currently Available to Norwegian Users to Support Diabetes Self-management	Not RCT + not app-based intervention + no effectiveness outcome
Jamshidnezhad et al.	2019	The Effects of Smartphone Applications on Patients Self-care with Hypertension: A Systematic Review Study	Not RCT
Jiménez-Chala	2022	Use of Mobile Applications to Increase Therapeutic Adherence in Adults: A Systematic Review	Not app-based intervention
Jiménez-Muñoz et al.	2022	Mobile applications for the management of chronic physical conditions: a systematic review	Not RCT
Jin et al.	2021	The Effectiveness of Technology-Based Interventions for Reducing Loneliness in Older Adults: A Systematic Review and	Not app-based intervention

		Meta-Analysis of Randomized Controlled Trials	
Jung et al.	2021	Cognitive Intervention Using Information and Communication Technology for Older Adults with Mild Cognitive Impairment: A Systematic Review and Meta-Analysis	Not app-based intervention
Jung et al.	2022	Promoting Physical Activity and Weight Loss With mHealth Interventions Among Workers: Systematic Review and Meta-analysis of Randomized Controlled Trials	Not app-based intervention
Jung et al.	2018	Effectiveness of telemonitoring intervention in children and adolescents with asthma: A systematic review and meta-analysis	Not English
Kalagara et al.	2022	Blood pressure management through application-based telehealth platforms: a systematic review and meta-analysis	Full text not accessible
Kaufman et al.	2022	E-Mentoring to Address Youth Health: A Systematic Review	Not RCT
Kerst et al.	2020	Smartphone applications for depression: a systematic literature review and a survey of health care professionals' attitudes towards their use in clinical practice	Not RCT
Khokhar et al.	2014	Effectiveness of mobile electronic devices in weight loss among overweight and obese populations: a systematic review and meta-analysis	Not app-based intervention
Khoo et al.	2021	mHealth Interventions to Address Physical Activity and Sedentary Behavior in Cancer Survivors: A Systematic Review	Not RCT

Kiani et al.	2022	Evaluation of m-Health-rehabilitation for respiratory disorders: A systematic review	Not RCT
Kim et al.	2022	The Clinical Effects of Type 2 Diabetes Patient Management Using Digital Healthcare Technology: A Systematic Review and Meta-Analysis	Not app-based intervention
Kim et al.	2018	Smartphone Apps for Autism Spectrum Disorder—Understanding the Evidence	Not RCT + not app-based intervention + no effectiveness outcome
Kim et al.	2021	Effectiveness of Mobile Health-Based Exercise Interventions for Patients with Peripheral Artery Disease: Systematic Review and Meta-Analysis	Not app-based intervention
Kim et al.	2023	Effectiveness of Digital Mental Health Tools to Reduce Depressive and Anxiety Symptoms in Low- and Middle-Income Countries: Systematic Review and Meta-analysis	Not app-based intervention
Kitsiou et al.	2021	Effectiveness of Mobile Health Technology Interventions forÂ Patients With Heart Failure: Systematic Review and Meta-analysis	Not app-based intervention
Kolcun et al.	2020	Systematic review of telemedicine in spine surgery	Not RCT
Königbauer et al.	2017	Internet- and mobile-based depression interventions for people with diagnosed depression: A systematic review and meta-analysis	Not app-based intervention
Kosa et al.	2019	Nutritional Mobile Applications for CKD Patients: Systematic Review	Not RCT

Kouvari et al.	2022	Digital Health Interventions for Weight Management in Children and Adolescents: Systematic Review and Meta-analysis	Not app-based intervention
Kraaijkamp et al.	2021	eHealth in Geriatric Rehabilitation: Systematic Review of Effectiveness, Feasibility, and Usability	Not RCT
Kumar et al.	2021	The Potential of mHealth as a Game Changer for the Management of Sickle Cell Disease in India	Not systematic review + not RCT + not app-based intervention + no effectiveness outcome
Langarizadeh et al.	2021	Mobile apps for weight management in children and adolescents; An updated systematic review	Population not diseased
Lara-Palomo et al.	2022	Efficacy of e-Health Interventions in Patients with Chronic Low-Back Pain: A Systematic Review with Meta-Analysis	Full text not accessible
Lau et al.	2022	Effect of digital cognitive behavioral therapy on psychological symptoms among perinatal women in high income-countries: A systematic review and meta-regression	Not app-based intervention
Lee et al.	2023	Effects of mobile health interventions on health-related outcomes in older adults with type 2 diabetes: A systematic review and meta-analysis	Not app-based intervention
Leung et al.	2021	Effectiveness of Using Mobile Technology to Improve Cognitive and Social Skills Among Individuals With Autism Spectrum Disorder: Systematic Literature Review	Not app-based intervention
Li et al.	2020	Technology-supported lifestyle interventions to improve maternal-fetal	Not app-based intervention

		outcomes in women with gestational diabetes mellitus: A meta-analysis	
Li et al. (excluded after data extraction stage)	2020	The Effectiveness of Self-Management of Hypertension in Adults Using Mobile Health: Systematic Review and Meta-Analysis	Not app-based intervention
Liang et al.	2011	Effect of mobile phone intervention for diabetes on glycaemic control: a meta-analysis	Not RCT + not app-based intervention
Lieber et al.	2015	Meta-analysis of telemonitoring to improve HbA1c levels: promise for stroke survivors	Not app-based intervention
Linardon	2020	Can Acceptance, Mindfulness, and Self-Compassion Be Learned by Smartphone Apps? A Systematic and Meta-Analytic Review of Randomized Controlled Trials	Population not diseased
Linardon	2019	The efficacy of app-supported smartphone interventions for mental health problems: a meta-analysis of randomized controlled trials	Population not diseased
Liu et al.	2020	Correction: Effectiveness of Mobile App-Assisted Self-Care Interventions for Improving Patient Outcomes in Type 2 Diabetes and/or Hypertension: Systematic Review and Meta-Analysis of Randomized Controlled Trials	Correction
Liu et al.	2017	Mobile health as a viable strategy to enhance stroke risk factor control: A systematic review and meta-analysis	Population not diseased + not app-based intervention
Liu et al.	2013	Reducing blood pressure with Internet-based interventions: a meta-analysis	Not RCT + not app-based intervention

Liu et al.	2015	Mobile phone intervention and weight loss among overweight and obese adults: a meta-analysis of randomized controlled trials	Population not diseased + not app-based intervention
Loucas et al.	2014	E-therapy in the treatment and prevention of eating disorders: A systematic review and meta-analysis	Population not diseased + not app-based intervention
Lu et al.	2021	Effectiveness of Telemonitoring for Reducing Exacerbation Occurrence in COPD Patients With Past Exacerbation History: A Systematic Review and Meta-Analysis	Not app-based intervention
Lu et al.	2019	Interactive Mobile Health Intervention and Blood Pressure Management in Adults	Not app-based intervention
Luo et al.	2022	Effectiveness of mobile health-based self-management interventions in breast cancer patients: a meta-analysis	Not RCT + not app-based intervention
Luxton et al.	2011	mHealth for mental health: Integrating smartphone technology in behavioral healthcare	Not systematic review + not RCT + population not diseased + not app-based intervention + no effectiveness outcome
Lv et al.	2021	Effects of Telemedicine and mHealth on Systolic Blood Pressure Management in Stroke Patients: Systematic Review and Meta-Analysis of Randomized Controlled Trials	Not app-based intervention
Machado et al.	2016	Smartphone apps for the self-management ofÂ low back pain: A systematic review	Not RCT + not app-based intervention
Magalhães et al.	2021	The Use of Mobile Applications for Managing Care Processes During	Not RCT

		Chemotherapy Treatments: A Systematic Review	
Maisto et al.	2021	Digital Interventions for Psychological Comorbidities in Chronic Diseases-A Systematic Review	Not app-based intervention
Malakouti et al.	2020	Effectiveness of self-help mobile telephone applications (apps) for suicide prevention: A systematic review	Not RCT
Mao et al.	2020	Impact and efficacy of mobile health intervention in the management of diabetes and hypertension: a systematic review and meta-analysis	Not app-based intervention
Marcolino et al.	2013	Telemedicine application in the care of diabetes patients: systematic review and meta-analysis	Not app-based intervention
Marthick et al.	2021	Supportive Care Interventions for People With Cancer Assisted by Digital Technology: Systematic Review	Not app-based intervention
Martin et al.	2021	The efficacy of mobile health interventions used to manage acute or chronic pain: A systematic review	Not RCT + not app-based intervention
Martos-Cabrera et al.	2020	Smartphones and Apps to Control Glycosylated Hemoglobin (HbA1c) Level in Diabetes: A Systematic Review and Meta-Analysis	Not app-based intervention
Mashhadi et al.	2021	Post Discharge mHealth and Teach-Back Communication Effectiveness on Hospital Readmissions: A Systematic Review	Not RCT + not app-based intervention
Matamala-Gomez et al.	2020	The Role of Engagement in Teleneurorehabilitation: A Systematic Review	Not app-based intervention

Matis et al.	2020	Mindfulness-Based Programs for Patients With Cancer via eHealth and Mobile Health: Systematic Review and Synthesis of Quantitative Research	Not app-based intervention
Matthews et al.	2016	Persuasive Technology in Mobile Applications Promoting Physical Activity: a Systematic Review	Not RCT
McHenry et al.	2019	A systematic review of portable electronic technology for health education in resource-limited settings	Not RCT
McLean et al.	2016	Digital interventions to promote self-management in adults with hypertension systematic review and meta-analysis	Not app-based intervention
Melbye et al.	2020	Smartphone-Based Self-Monitoring, Treatment, and Automatically Generated Data in Children, Adolescents, and Young Adults With Psychiatric Disorders: Systematic Review	Not RCT
Michaud et al.	2021	Assessing the Impact of Telemonitoring-Facilitated Lifestyle Modifications on Diabetes Outcomes: A Systematic Review and Meta-Analysis	Not app-based intervention
Miller et al.	2017	Mobile Technology Interventions for Asthma Self-Management: Systematic Review and Meta-Analysis	Not app-based intervention
Miura et al.	2023	App-based interventions for the prevention of postpartum depression: a systematic review and meta-analysis	Population not diseased
Mobasheri et al.	2015	The uses of smartphones and tablet devices in surgery: A systematic review of the literature	Population not diseased

Moman et al.	2019	A Systematic Review and Meta-analysis of Unguided Electronic and Mobile Health Technologies for Chronic Pain-Is It Time to Start Prescribing Electronic Health Applications?	Not app-based intervention
Morris et al.	2023	The Impact of Digital Health Interventions for the Management of Type 2 Diabetes on Health and Social Care Utilisation and Costs: A Systematic Review	Not RCT
Moses et al.	2021	Application of Smartphone Technologies in Disease Monitoring: A Systematic Review	Not RCT + not app-based intervention
Moshe et al.	2021	Digital interventions for the treatment of depression: A meta-analytic review	Not app-based intervention
Mt et al.	2019	Smartphone app for asthma self-management ‚Äì a literature review of contents and functions	Not RCT
Nair et al.	2018	The effectiveness of telemedicine interventions to address maternal depression: A systematic review and meta-analysis	Not app-based intervention
Najm et al.	2019	Mobile Health Apps for Self-Management of Rheumatic and Musculoskeletal Diseases: Systematic Literature Review	Not RCT
Neumayr et al.	2021	Psych-APP-Therapy: Smartphone-Based Interventions in Psychotherapy A Systematic Review	Not English
Nicholl et al.	2017	Digital Support Interventions for the Self-Management of Low Back Pain: A Systematic Review	Not app-based intervention

Ning et al.	2022	Effect and feasibility of gamification interventions for improving physical activity and health-related outcomes in cancer survivors: an early systematic review and meta-analysis	Not app-based intervention
Nkhoma et al.	2021	Digital interventions self-management education for type 1 and 2 diabetes: A systematic review and meta-analysis	Not app-based intervention
Nwolise et al.	2016	Preconception Care Education for Women With Diabetes: A Systematic Review of Conventional and Digital Health Interventions	Not RCT
Obrero-Gaitán et al.	2022	Digital and Interactive Health Interventions Minimize the Physical and Psychological Impact of Breast Cancer, Increasing Women's Quality of Life: A Systematic Review and Meta-Analysis	Not RCT
Oliveira et al.	2021	Effectiveness of Mobile App-Based Psychological Interventions for College Students: A Systematic Review of the Literature	Not RCT
Or et al.	2014	Does the use of consumer health information technology improve outcomes in the patient self-management of diabetes? A meta-analysis and narrative review of randomized controlled trials	Not app-based intervention
Park et al.	2019	Effect of Mobile Health on Obese Adults: A Systematic Review and Meta-Analysis	Not app-based intervention
Parker et al.	2018	Electronic, mobile and telehealth tools for vulnerable patients with chronic disease: a systematic review and realist synthesis	Not RCT + not app-based intervention

Pastora-Bernal et al.	2017	Evidence of Benefit of Telerehabilitation After Orthopedic Surgery: A Systematic Review	Not RCT + not app-based intervention
Patterson et al.	2021	Smartphone applications for physical activity and sedentary behaviour change in people with cardiovascular disease: A systematic review and meta-analysis	Not RCT
Patterson et al.	2022	Behaviour change techniques in cardiovascular disease smartphone apps to improve physical activity and sedentary behaviour: Systematic review and meta-regression	Not RCT
Payne et al.	2015	Behavioral functionality of mobile apps in health interventions: a systematic review of the literature	Not RCT
Pérez-Jover et al.	2019	Mobile Apps for Increasing Treatment Adherence: Systematic Review	Not RCT
Pi et al.	2021	Meta-Analysis of RCTs of Technology-Assisted Parent-Mediated Interventions for Children with ASD	Not app-based intervention
Planas et al.	2021	Technological prescription: evaluation of the effectiveness of mobile applications to improve depression and anxiety. Systematic review	Not RCT
Popovici et al.	2023	Benefits of Using Smartphones and Other Digital Methods in Achieving Better Cardiac Rehabilitation Goals: A Systematic Review and Meta-Analysis	Not app-based intervention
Porter et al.	2016	The Effect of Using Mobile Technology-Based Methods That Record Food or	Not RCT + not app-based intervention

		Nutrient Intake on Diabetes Control and Nutrition Outcomes: A Systematic Review	
Powell et al.	2017	What is the level of evidence for the use of currently available technologies in facilitating the self-management of difficulties associated with ADHD in children and young people? A systematic review	Not RCT
Punukollu et al.	2019	Use of mobile apps and technologies in child and adolescent mental health: a systematic review	Not RCT + not app-based intervention
Qin et al.	2022	Effect of Mobile Phone App-Based Interventions on Quality of Life and Psychological Symptoms Among Adult Cancer Survivors: Systematic Review and Meta-analysis of Randomized Controlled Trials	Not app-based intervention
Ramachandran et al.	2022	Effectiveness of home-based cardiac telerehabilitation as an alternative to Phase 2 cardiac rehabilitation of coronary heart disease: a systematic review and meta-analysis	Not app-based intervention
Rathbone et al.	2017	Assessing the Efficacy of Mobile Health Apps Using the Basic Principles of Cognitive Behavioral Therapy: Systematic Review	Population not diseased
Rebolledo Del Toro et al.	2023	Effectiveness of mobile telemonitoring applications in heart failure patients: systematic review of literature and meta-analysis	Not app-based intervention

Riazi et al.	2015	Managing diabetes mellitus using information technology: a systematic review	Not RCT + not app-based intervention
Rincon et al.	2017	Mobile Phone Apps for Quality of Life and Well-Being Assessment in Breast and Prostate Cancer Patients: Systematic Review	Not RCT
Romeo et al.	2019	Can Smartphone Apps Increase Physical Activity? Systematic Review and Meta-Analysis	Population not diseased
Rootes-Murdy et al.	2018	Mobile technology for medication adherence in people with mood disorders: A systematic review	Not app-based intervention
Rush et al.	2020	Transitions in Atrial Fibrillation Care: A Systematic Review	Not RCT + not app-based intervention
Russell-Minda et al.	2009	Health technologies for monitoring and managing diabetes: a systematic review	Not app-based intervention
Saevarsdottir et al.	2023	Mobile Apps and Quality of Life in Patients With Breast Cancer and Survivors: Systematic Literature Review	Not RCT
Saramago et al.	2021	Digital Interventions for Generalized Anxiety Disorder (GAD): Systematic Review and Network Meta-Analysis	Not app-based intervention
Sarfo et al.	2018	Tele-Rehabilitation after Stroke: An Updated Systematic Review of the Literature	Not RCT + not app-based intervention
Schippers et al.	2017	A meta-analysis of overall effects of weight loss interventions delivered via mobile phones and effect size differences according to delivery mode, personal	Not app-based intervention

		contact, and intervention intensity and duration	
Semper et al.	2016	A systematic review of the effectiveness of smartphone applications that encourage dietary self-regulatory strategies for weight loss in overweight and obese adults	Not RCT
Sequi-Dominguez et al.	2020	Effectiveness of Mobile Health Interventions Promoting Physical Activity and Lifestyle Interventions to Reduce Cardiovascular Risk Among Individuals With Metabolic Syndrome: Systematic Review and Meta-Analysis	Not RCT
Serrano-Ripoll et al.	2022	Impact of Smartphone App-Based Psychological Interventions for Reducing Depressive Symptoms in People With Depression: Systematic Literature Review and Meta-analysis of Randomized Controlled Trials	Not app-based intervention
Seyyedi et al.	2019	Mobile phone applications to overcome malnutrition among preschoolers: a systematic review	Not RCT
Shaker et al.	2023	Psychiatric Treatment Conducted via Telemedicine Versus In-Person Modality in Posttraumatic Stress Disorder, Mood Disorders, and Anxiety Disorders: Systematic Review and Meta-Analysis	Not app-based intervention
Shi et al.	2023	Mobile Health Application-Based Interventions to Improve Self-management of Chemotherapy-Related Symptoms Among People with Breast Cancer Who	No effectiveness outcome

		Are Undergoing Chemotherapy: A Systematic Review	
Shields et al.	2018	Mobile direct observation of therapy (MDOT) - A rapid systematic review and pilot study in children with asthma	Not RCT
Shrivastava et al.	2021	Mobile App Interventions to Improve Medication Adherence Among Type 2 Diabetes Mellitus Patients: A Systematic Review of Clinical Trials	Not RCT
Silva et al.	2020	Effectiveness of Mobile Applications Running on Smartphones to Promote Physical Activity: A Systematic Review with Meta-Analysis	Population not diseased
Sin et al.	2020	Digital Interventions for Screening and Treating Common Mental Disorders or Symptoms of Common Mental Illness in Adults: Systematic Review and Meta-analysis	Not app-based intervention
Six et al.	2021	Examining the Effectiveness of Gamification in Mental Health Apps for Depression: Systematic Review and Meta-analysis	Not RCT
Slater et al.	2017	End User and Implementer Experiences of mHealth Technologies for Noncommunicable Chronic Disease Management in Young Adults: Systematic Review	Not RCT
Slugocki et al.	2019	A Review of Emerging Technologies in Diabetes Management for Multiple-Dose Insulin-Injecting Patients With Type 2 Diabetes Who Self-monitor Blood Glucose	Not RCT + not app-based intervention + no effectiveness outcome

So et al.	2018	Telehealth for diabetes self-management in primary healthcare: A systematic review and meta-analysis	Not app-based intervention
Son et al.	2020	Effectiveness of Mobile Phone-Based Interventions for Improving Health Outcomes in Patients with Chronic Heart Failure: A Systematic Review and Meta-Analysis	Not app-based intervention
Song et al.	2023	Randomized Controlled Trials of Digital Mental Health Interventions on Patients with Schizophrenia Spectrum Disorder: A Systematic Review	Not app-based intervention
Stark et al.	2022	App-Based Rehabilitation in Back Pain, a Systematic Review	Not RCT
Stefanopoulou et al.	2020	Are digital interventions effective in reducing suicidal ideation and self-harm? A systematic review	Not app-based intervention
Stephani et al.	2016	A systematic review of randomized controlled trials of mHealth interventions against non-communicable diseases in developing countries	Not app-based intervention
Stephen et al.	2022	Using mHealth applications for self-care - An integrative review on perceptions among adults with type 1 diabetes	Not RCT + no effectiveness outcome
Stevens et al.	2022	The effectiveness of digital health technologies for patients with diabetes mellitus: A systematic review	Not app-based intervention
Steubl et al.	2021	Mechanisms of change in Internet- and mobile-based interventions for PTSD: a systematic review and meta-analysis	Not app-based intervention

Stogios et al.	2020	Advancing Digital Health Interventions as a Clinically Applied Science for Blood Pressure Reduction: A Systematic Review and Meta-analysis	Not app-based intervention
Sua et al.	2020	Effectiveness of mobile phone-based self-management interventions for medication adherence and change in blood pressure in patients with coronary heart disease: A systematic review and meta-analysis	Not app-based intervention
Subedi et al.	2020	Implementation of telerehabilitation interventions for the self-management of cardiovascular disease: systematic review	Not RCT + not app-based intervention
Sun et al.	2019	Improving Glycemic Control in Adults and Children With Type 1 Diabetes With the Use of Smartphone-Based Mobile Applications: A Systematic Review	Not RCT
Sunjaya et al.	2020	Efficacy, patient-doctor relationship, costs and benefits of utilizing telepsychiatry for the management of post-traumatic stress disorder (PTSD): a systematic review	Not app-based intervention
Sunjaya et al.	2022	Efficacy of self-management mobile applications for patients with breathlessness: Systematic review and quality assessment of publicly available applications	Not RCT
Svendsen et al.	2018	eHealth Technologies as an intervention to improve adherence to topical antipsoriatics: a systematic review	Not app-based intervention
Tanhapour et al.	2023	The effect of personalized intelligent digital systems for self-care training on	Not app-based intervention

		type II diabetes: a systematic review and meta-analysis of clinical trials	
Tao et al.	2017	Effects of consumer-oriented health information technologies in diabetes management over time: a systematic review and meta-analysis of randomized controlled trials	Not app-based intervention
Theng et al.	2015	The Use of Videogames, Gamification, and Virtual Environments in the Self-Management of Diabetes: A Systematic Review of Evidence	Not RCT + not app-based intervention
Thurnheer et al.	2018	Benefits of Mobile Apps in Pain Management: Systematic Review	Not RCT
Timmers et al.	2020	Educating Patients by Providing Timely Information Using Smartphone and Tablet Apps: Systematic Review	Not app-based intervention
Tonga et al.	2022	Components, design and effectiveness of digital physical rehabilitation interventions for older people: A systematic review	Not app-based intervention
Toresdahl et al.	2021	A Systematic Review of Telehealth and Sport-Related Concussion: Baseline Testing, Diagnosis, and Management	Not RCT
Torok et al.	2020	Suicide prevention using self-guided digital interventions: a systematic review and meta-analysis of randomised controlled trials	Not app-based intervention
Triantafyllidis et al.	2023	Mobile App Interventions for Parkinson's Disease, Multiple Sclerosis and Stroke: A Systematic Literature Review	Not RCT
Triberti et al.	2019	eHealth for improving quality of life in breast cancer patients: A systematic review	Not RCT + not app-based intervention

Tsai et al.	2022	Evaluating the effectiveness and quality of mobile applications for perinatal depression and anxiety: A systematic review and meta-analysis	Not app-based intervention
Tufts et al.	2015	Novel interventions for HIV self-management in African American women: a systematic review of mHealth interventions	Not app-based intervention
Turner et al.	2015	Prevention and treatment of pediatric obesity using mobile and wireless technologies: a systematic review	Not app-based intervention
Uthman et al.	2019	Comparison of mHealth and Face-to-Face Interventions for Smoking Cessation Among People Living With HIV: Meta-Analysis	Not app-based intervention
Välimäki et al.	2013	Information and communication technology in patient education and support for people with schizophrenia	Not app-based intervention
Valentijn et al.	2022	Digital Health Interventions for Musculoskeletal Pain Conditions: Systematic Review and Meta-analysis of Randomized Controlled Trials	Not app-based intervention
van Ameringen et al.	2017	There is an app for that! The current state of mobile applications (apps) for DSM-5 obsessive-compulsive disorder, posttraumatic stress disorder, anxiety and mood disorders	Not RCT + not app-based intervention
Vázquez-de Sebastián et al.	2021	Analysis of Effectiveness and Psychological Techniques Implemented in mHealth Solutions for Middle-Aged and	Not RCT + not app-based intervention

		Elderly Adults with Type 2 Diabetes: A Narrative Review of the Literature	
Veiga et al.	2022	A systematic review on smartphone use for activity monitoring during exercise therapy in intermittent claudication	Full text not accessible
Vergani et al.	2019	Training Cognitive Functions Using Mobile Apps in Breast Cancer Patients: Systematic Review	Not RCT
Versluis et al.	2016	Changing Mental Health and Positive Psychological Well-Being Using Ecological Momentary Interventions: A Systematic Review and Meta-analysis	Population not diseased
Viola et al.	2020	Digital interventions for adolescent and young adult cancer survivors	Not RCT + not app-based intervention
Wang et al.	2019	Mobile health in the management of type 1 diabetes: a systematic review and meta-analysis	Not app-based intervention
Wang et al.	2022	Effectiveness of Smartphone-Based Lifestyle Interventions on Women with Gestational Diabetes: A Systematic Review and Meta-Analysis of Randomized Controlled Trials	Not app-based intervention
Wang et al.	2023	Effects of Patient-Reported Outcome Tracking and Health Information Provision via Remote Patient Monitoring Software on Patient Outcomes in Oncology Care: A Systematic Review and Meta-Analysis	Not app-based intervention
Weisel et al.	2019	Standalone smartphone apps for mental health-a systematic review and meta-analysis	Population not diseased

Wesley et al.	2015	A review of mobile applications to help adolescent and young adult cancer patients	Not systematic review + not RCT + not app-based intervention + no effectiveness outcome
Widdison et al. (excluded after data extraction stage)	2022	Effectiveness of mobile apps to improve urinary incontinence: a systematic review of randomised controlled trials	Not RCT
Winders et al.	2021	The effects of mobile health on emergency care in low- and middle-income countries: A systematic review and narrative synthesis	Not RCT + population not diseased + not app-based intervention
Witt et al.	2017	Effectiveness of online and mobile telephone applications ('apps') for the self-management of suicidal ideation and self-harm: a systematic review and meta-analysis	Not RCT + not app-based intervention
Wu et al.	2021	Smartphone apps for depression and anxiety: a systematic review and meta-analysis of techniques to increase engagement	Not RCT
Wu et al.	2018	Effectiveness of smartphone technologies on glycaemic control in patients with type 2 diabetes: systematic review with meta-analysis of 17 trials	Not app-based intervention
Wu et al.	2017	Correction: Mobile App-Based Interventions to Support Diabetes Self-Management: A Systematic Review of Randomized Controlled Trials to Identify Functions Associated with Glycemic Efficacy	Correction

Xiao et al.	2018	Effectiveness of mHealth Interventions for Asthma Self-Management: A Systematic Review and Meta-Analysis	Not RCT + not app-based intervention
Xiong et al.	2018	Effectiveness of mHealth Interventions in Improving Medication Adherence Among People with Hypertension: a Systematic Review	Not RCT
Xu	2021	The efficacy of mobile health in alleviating risk factors related to the occurrence and development of coronary heart disease: A systematic review and meta-analysis	Not app-based intervention
Xu et al.	2022	The Effects of mHealth-Based Gamification Interventions on Participation in Physical Activity: Systematic Review	Not RCT
Yang et al.	2019	The Comparative Effectiveness of Mobile Phone Interventions in Improving Health Outcomes: Meta-Analytic Review	Not app-based intervention
Yap et al.	2021	Effectiveness of technology-based psychosocial interventions on diabetes distress and health-relevant outcomes among type 2 diabetes mellitus: A systematic review and meta-analysis	Not app-based intervention
Yen et al.	2023	Smartphone app-based interventions targeting physical activity for weight management: A meta-analysis of randomized controlled trials	Population not diseased
Yien et al.	2021	Effect of Mobile Health Technology on Weight Control in Adolescents and Preteens: A Systematic Review and Meta-Analysis	Not app-based intervention

Young et al.	2018	Efficacy of online lifestyle interventions targeting lifestyle behaviour change in depressed populations: A systematic review	Not app-based intervention
Yun et al.	2018	Comparative Effectiveness of Telemonitoring Versus Usual Care for Heart Failure: A Systematic Review and Meta-analysis	Not app-based intervention
Zangger et al.	2023	Benefits and Harms of Digital Health Interventions Promoting Physical Activity in People With Chronic Conditions: Systematic Review and Meta-Analysis	Not app-based intervention
Zeng et al.	2022	Impact of mobile health and telehealth technology on medication adherence of stroke patients: a systematic review and meta-analysis of randomized controlled trials	Not app-based intervention
Zhou et al.	2022	Effectiveness of mHealth interventions for improving hypertension control in uncontrolled hypertensive patients: A meta-analysis of randomized controlled trials	Not app-based intervention
Zhou et al.	2022	Effectiveness of Mobile Medical Apps in Ensuring Medication Safety Among Patients With Chronic Diseases: Systematic Review and Meta-analysis	Not app-based intervention
Zhou et al.	2022	The effectiveness of mHealth interventions on postpartum depression: A systematic review and meta-analysis	Not app-based intervention

Zhou et al.	2023	Effectiveness of smartphone-assisted cardiac rehabilitation: a systematic review and meta-analysis	Not app-based intervention

Supplementary Table 2. General review characteristics of 48 included reviews on the effectiveness of app-based health interventions

First Author (Year) [reference number in manuscript]	Time period covered for review		Eligibility criteria of origin of primary studies	Number of articles screened after de-duplication	Number of included articles	Number of articles for meta-analysis	Range of Follow-up duration in months (Median)
	Start date	End date					
Al-Arkee (2021) [16]	Inception	January, 2020	N/A	1679	16	9	1 - 12 (3)
Amalindah (2020) [50]	January, 2016	January, 2020	N/A	3085	12	N/A	3 - 12 (-)
Armitage (2020) [17]	1990	November, 2018	N/A	9971	9	9	1 - 4 (3)
Bonoto (2017) [18]	2008	2016	N/A	1236	13	13	1 - 12 (6)
Cai (2020) [19]	Inception	May, 2019	N/A	1741	14	14	3 - 12 (6)
Chew (2022) [41]	Inception	March, 2022	N/A	1992	16	16	3 - 24 (7)
Chew (2023) [40]	Inception	June, 2022	N/A	878	14	14	2 - 24 (6)
Cui (2016) [20]	2005	June, 2016	N/A	2596	13	6	3 - 12 (10)
Davergne (2023) [42]	N/A	March, 2022	N/A	1641	10	10	0.75 - 12 (3)
Didyk (2021) [51]	2008	June, 2021	N/A	1059	6	N/A	1 - 3 (1.5)
DiFilippo (2015) [52]	January, 2008	October, 2013 and January, 2014	N/A	17032	3	N/A	2 - 6 (6)
El-Gayar (2021) [21]	January, 2010	October, 2020	N/A	488	21	21	3 - 18 (6)
Enricho Nkhoma (2021) [22]	Inception	July 2020	N/A	3080	6	5	1.5 - 6 (3)
Han (2020) [15]	N/A	August, 2019	Chinese patients	56	18	18	1 - 24 (-)
He (2021) [23]	January, 2008	January, 2021	N/A	4872	19	19	3 - 12 (mean 7.3)
Hernández-Gómez (2022) [61]	Inception	June, 2021	N/A	271	7	N/A	0.75 - 8 (6)
Hou (2016) [24]	January, 1996	June, 2015	N/A	4238	18	18	2 - 12 (6)
Hou (2018) [25]	June, 2015	May, 2017	N/A	7433	21	21	1.5 - 12 (6)
Hou (2022) [62]	Inception	October, 2021	N/A	8428	6	N/A	3 - 24 (12)
Hrynyschyn (2021) [53]	N/A	May, 2020	N/A	450	8	N/A	0.75 - 6 (-)
Hyun (2021) [26]	N/A	October, 2020	N/A	10391	14	14	3 - 12 (6)
Karatas (2022) [54]	Inception	November, 2020	N/A	18701	7	N/A	2 - 13 (6)
Kassavou (2022) [27]	2000	August, 2021	N/A	4474	15	15	1 - 12 (3)
Kim (2022) [28]	N/A	October, 2020	N/A	5182	14	14	0.75 - 12 (5)
Lee (2018) [55]	January, 2005	March, 2016	N/A	2217	13	N/A	0.5 - 12 (5)

Leme Nagib (2020) [56]	N/A	June, 2019	N/A	11108	3	N/A	3 - 4 (3)
Liu (2020) [29]	January, 2007	January, 2019	N/A	2263	24	24	2 - 12 (6)
Lu (2022) [43]	January, 2012	April, 2020	N/A	3921	15	15	1 - 3 (1)
Lunde (2018) [30]	N/A	February, 2017	N/A	1233	9	7	3 - 12 (6)
Marcano Belisario (2013) [57]	2000	June, 2013	N/A	973	2	N/A	6 (6)
Mikulski (2021) [31]	N/A	July, 2020	N/A	1199	10	9	1 - 18 (-)
Moon (2019) [32]	2009	March, 2019	N/A	1100	7	7	0 - 4 (-)
Moreno-Ligero (2023a) [44]	N/A	April, 2023	N/A	445	13	11	1 - 3 (1.6)
Moreno-Ligero (2023b) [60]	N/A	March, 2022	N/A	395	22	N/A	0.75 - 12 (2)
Özden (2023a) [45]	1990	2021	N/A	2175	5	2	1.5 - 12 (4)
Özden (2023b) [46]	Inception	January, 2022	N/A	1595	6	3	1 - 24 (3)
Park (2020) [33]	Inception	January, 2019	N/A	545	17	17	0.5 - 6 (-)
Peng (2020) [34]	N/A	December, 2018	N/A	2608	14	14	1 - 12 (3)
Pi (2023) [47]	N/A	18-Feb-23	N/A	858	9	9	1 - 12 (6)
Seegan (2023) [49]	January, 2007	October, 2022	N/A	2235	36	36	0.5 - 3 (1)
Shaw (2020) [35]	Inception	April, 2019	N/A	971	13	10	0.5 - 12 (4)
Thompson (2023) [48]	Inception	January, 2022	N/A	7978	11 of 10 studies	9	1 - 12 (2)
Whitehead (2016) [58]	2005	2016	N/A	893	9	N/A	3 - 12 (6)
Wickersham (2019) [59]	2007	February, 2018	N/A	421	5	N/A	1 - 12 (2)
Wu (2017) [37]	January, 2007	May, 2016	N/A	3131	13	13	3 - 12 (6)
Wu (2019) [36]	January, 2006	May, 2018	N/A	2232	26	18	3 - 12 (6)
Xu (2020) [38]	N/A	May, 2020	N/A	134	8	6	1.5 - 18 (6)
Yang (2018) [39]	N/A	November, 2017	N/A	4072	8	6	1 - 12 (7.5)
N/A: The information was not available in the text							

Supplementary Table 3. Interrater-Reliability for Quality Appraisal

Domain	Cohen's Kappa
D1: Inclusion of PICO elements in review question	0.7156
D2: Protocol registration	0.2672
D3: Explain selection of study design	0.4146
D4: Adequacy of the literature search	0.2387
D5: Duplicate study selection	0.5328
D6: Duplicate data extraction	0.7372
D7: Justification for excluding studies	0.7798
D8: Description of studies	0.1314
D9: Risk of bias from studies included in review	0.3514
D10: Report sources of funding for primary studies	0.7391
D11: Appropriateness of met-analytical methods	0.7303
D12: Impact of risk of bias assessment on evidence	0.7477
D13: Consideration of risk of bias in interpretation of results	0.4667
D14: Explanation for heterogeneity	0.4167
D15: Assessment of presence and likely impact of publication bias	0.8366
D16: Report potential conflicts of interest and funding sources by review authors	-0.0213
Overall	0.6671

Supplementary Table 4. Overview of included populations, interventions, comparators, and outcomes of 48 included reviews on the effectiveness of app-based health interventions

First Author (Year) [reference number in manuscript]	Countries of Included Studies	Health Conditions in Included Studies	Intervention Characteristics	Control Group Treatments	Outcomes Measured
Al-Arkee (2021) [16]	• Australia<br>• US<br>• China<br>• Sweden<br>• Korea<br>• Spain<br>• Germany<br>• Ghana	• Atrial fibrillation<br>• CHD<br>• HF<br>• Hypercholesterolemia<br>• Hypertension<br>• MI<br>• Stroke<br>• Diabetes	• Reminder<br>• Education	• SMS text messages<br>• No/usual care<br>• Paper diary<br>• WeChat app<br>• Standard care<br>• Phone<br>• Pillbox	• Medication adherence<br>• SBP/DBP<br>• LDL-c/Total cholesterol<br>• Patient activation measures<br>• Patients' knowledge of their CVD<br>• QoL<br>• HbA1c
Amalindah (2020) [50]	N/A	• T1DM<br>• T2DM	• Track health data<br>• Patient feedback and reminders<br>• Diabetes education	• Usual care<br>• Standard education<br>• Use of a paper diary	• HbA1c<br>• LDL-c/HDL-c<br>• BMI<br>• Hypoglycemia effect<br>• Psychological aspects (self-efficacy, depression, QoL, knowledge, and adherence)
Armitage (2020) [17]	• US<br>• UK<br>• Germany<br>• Spain<br>• Australia<br>• Denmark	• Depression<br>• Ischaemic stroke requiring secondary oral anticoagulation<br>• Parkinson's disease<br>• CVD<br>• Multimorbidity<br>• Hypertension<br>• CHD	• Tailored prompts and cues<br>• Monitoring of behavior by others without feedback<br>• Feedback on behavior<br>• Social support<br>• Habit formation<br>• Goal setting of behavior	N/A	• Medication adherence

First Author (Year) [reference number in manuscript]	Countries of Included Studies	Health Conditions in Included Studies	Intervention Characteristics	Control Group Treatments	Outcomes Measured
		• Cardiovascular stent<br>• Psoriasis			
Bonoto (2017) [18]	• US<br>• Italy<br>• England<br>• Norway<br>• Germany<br>• Finland<br>• Australia<br>• Netherlands<br>• France<br>• Spain	• T1DM<br>• T2DM	• Health data storage<br>• Feedback on physiological parameters<br>• Motivational messages<br>• Function for healthy diet and exercise<br>• Functions for insulin dosage adjustment<br>• Chat and video conferencing with health professionals<br>• Alarm for drug therapy compliance<br>• Health goals<br>• Calculating carbohydrate intake	• Standardized health treatment	• HbA1c/FBG<br>• Body weight<br>• SBP/DBP<br>• Total cholesterol/ HDL-c/ LDL-c/ TG
Cai (2020) [19]	• Spain<br>• Mexico<br>• US<br>• Norway<br>• Finland<br>• Korea<br>• India<br>• China<br>• Japan<br>• Canada	• T2DM	• Exercise recording/ monitoring<br>• Tailored diet-based information<br>• Dietary, weight, glucose recording/monitoring<br>• Medication adjustment support<br>• Feedback	• Standardized counselling<br>• Care management<br>• Group education<br>• Accelerometer<br>• Standardized diabetes self-management<br>• Usual care<br>• Standard care	• Anthropometric measurements: body weight/ BMI/ waist circumference/ fat mass/ percentage of body fat

First Author (Year) [reference number in manuscript]	Countries of Included Studies	Health Conditions in Included Studies	Intervention Characteristics	Control Group Treatments	Outcomes Measured
				• Standard medical care<br>• Routine outpatient care<br>• Self-care regimen<br>• Usual care with education<br>• Health coach support<br>• In-person meetings, scheduled routine follow-up	
Chew (2022) [41]	• UK<br>• US<br>• Australia<br>• Japan<br>• China	• Overweight and obesity	• Food logging<br>• Real-time self-monitoring of weight, diet and exercise<br>• Tailored messages according to progress<br>• Feedback<br>• Personalized progress reports<br>• Libraries of diet and physical activity ideas<br>• Remote progress monitoring<br>• Education<br>• Goal setting<br>• Discussion forum	• Diary book<br>• Basic version of apps<br>• Newsletters<br>• Printed material<br>• Usual care<br>• Other apps<br>• Information website<br>• No treatment<br>• Waitlist<br>• Text messages<br>• Emails<br>• Self-monitoring tools<br>• DVDs	• Weight loss/waist circumference<br>• HbA1c<br>• HDL-c/LDL-c<br>• Total energy intake<br>• SBP/DBP

First Author (Year) [reference number in manuscript]	Countries of Included Studies	Health Conditions in Included Studies	Intervention Characteristics	Control Group Treatments	Outcomes Measured
			• Peer information sharing on weight-related challenges<br>• Behavioural change techniques	• Bite counter	
Chew (2023) [40]	• US<br>• Australia<br>• Belgium<br>• Korea<br>• Japan	• Overweight or obesity	• Education and coaching<br>• Self-monitoring of behaviors<br>• Goal setting and planning<br>• Social support<br>• Problem solving<br>• Rewards<br>• Reduction of negative emotions	• Self-monitoring apps<br>• Usual care<br>• Websites<br>• Waitlists<br>• Group education<br>• Self-monitoring<br>• Print resources	• Weight loss/BMI/waist circumference/body fat<br>• SBP/DBP<br>• Total cholesterol/HDL-c/LDL-c/TG<br>• HbA1c<br>• Total calorie consumption<br>• Physical activity
Cui (2016) [20]	• Finland<br>• Norway<br>• US<br>• Korea<br>• Spain<br>• UK<br>• Canada<br>• Netherlands	• T2DM	• Self-monitoring/management<br>• Education<br>• Tele-assistance<br>• Real time feedback	• Usual care	• HbA1c with/without feedback group<br>• SBP/DBP<br>• LDL-c/HDL-c/TG/total cholesterol<br>• Body weight<br>• Physical activity<br>• Medication use changes
Davergne (2023) [42]	• Australia<br>• New Zealand<br>• Portugal<br>• Austria<br>• US<br>• China<br>• Hong Kong	• Musculoskeletal disorder, neurological disorders	• Real-time biofeedback through wearable motion sensors<br>• Feedback on steps per day<br>• Home exercise program	• Usual care<br>• Home exercise program<br>• Advice to stay physically active	• Physical function<br>• QoL<br>• Adherence<br>• Self-confidence<br>• Health care use<br>• Adverse events

First Author (Year) [reference number in manuscript]	Countries of Included Studies	Health Conditions in Included Studies	Intervention Characteristics	Control Group Treatments	Outcomes Measured
			• Home-based physiotherapy sessions	• Education pieces regarding self-care	
Didyk (2021) [51]	• India<br>• US<br>• Nigeria<br>• Germany<br>• China	• Non-specific lower back pain	• Personalized activity and home exercise programs<br>• Reminders to target engagement and compliance<br>• Education behavioral change techniques	• Usual care<br>• Physiotherapy in-clinic Mckenzie therapy<br>• Usual physiotherapy care	• Pain intensity<br>• Disability/function<br>• Pain self-efficacy<br>• QoL<br>• Adherence to home exercise
DiFilippo (2015) [52]	• Australia<br>• UK<br>• US	• Obesity/ overweight	• Dashboard for information tracking and display<br>• Prompts/ messages<br>• Weigh loss goal setting<br>• Food and physical activity diary	• Meal replacement<br>• Information for meal replacement<br>• Weight loss resources web<br>• Paper diary/book<br>• Podcast	• Weight loss<br>• Perceived motivation<br>• Positive affect<br>• Adherence to diet monitoring
El-Gayar (2021) [21]	• US<br>• Netherlands<br>• China<br>• Norway<br>• Australia<br>• Taiwan<br>• Canada	• T1DM<br>• T2DM	• Record and store data<br>• Health behavior tracking<br>• Self-management education<br>• Supports with messages and prompts<br>• Tailored feedback	N/A	• HbA1c

First Author (Year) [reference number in manuscript]	Countries of Included Studies	Health Conditions in Included Studies	Intervention Characteristics	Control Group Treatments	Outcomes Measured
	• Finland<br>• France<br>• India<br>• Indonesia<br>• Italy<br>• Japan<br>• Sri Lanka<br>• UK		• Goal settings<br>• Communication with healthcare providers		
Enricho Nkhoma (2021) [22]	• Austria<br>• Spain<br>• Singapore<br>• Poland<br>• India<br>• South Korea	• T2DM	• Data recording<br>• Medication scheduling<br>• Reminder<br>• Tracking<br>• Assessments<br>• Monitoring<br>• Interactions with providers	• Standard blister<br>• Usual care	• Medication adherence<br>• Patient adherence<br>• HbA1c<br>• BMI<br>• BP<br>• Cholesterol
Han (2020) [15]	• China	• Hypertension	• Health education<br>• Doctor-patient communication<br>• Personalized lifestyle and medication guidance<br>• Medication reminders<br>• Lifestyle modification	N/A	• SBP/DBP

First Author (Year) [reference number in manuscript]	Countries of Included Studies	Health Conditions in Included Studies	Intervention Characteristics	Control Group Treatments	Outcomes Measured
He (2021) [23]	• South Korea<br>• Australia<br>• China<br>• Canada<br>• Mexico<br>• Netherlands<br>• Norway<br>• India<br>• US<br>• Japan	• T2DM	• Delivery of educational information and health consultation<br>• Provision of feedback<br>• Data management (cardiometabolic factors and self-management behaviours)<br>• Trend report and alert	• Usual care<br>• Standard care<br>• Consultation<br>• Maintain previous diabetes management/rout ine<br>• Regular education<br>• Follow-up<br>• Monitoring<br>• Feedback	• HbA1c<br>• Self-management (diet management, exercise management, medication adherence, blood glucose self-monitoring, foot care)<br>• Psychological wellbeing (depression, distress)<br>• HDL-c/ total cholesterol/ LDL-c/ TG<br>• SBP/DBP<br>• BMI<br>• QoL<br>• Participant engagement evaluation
Hernández-Gómez (2022) [61]	• Finland<br>• Denmark	• Major depression disorder, depression	• CBT<br>• Mindfulness-Based Stress Reduction<br>• Mindfulness- Based Cognitive Therapy<br>• Behavioral Activation Therapy<br>• Psychoeducation	• Face to face therapy<br>• Other apps which included mindfulness techniques<br>• Other apps to monitor mood and sleep quality and quantity<br>• Other included mindfulness techniques<br>• Email messages	• Depressive symptomatology<br>• Rate of and accumulated duration of psychiatric admissions<br>• QoL<br>• Perceived and unspecified psychological stress<br>• Anxiety levels<br>• Medication side effects<br>• Internalization of mindfulness skills and resilience<br>• Dysfunctional attitudes<br>• Sleep disturbances

First Author (Year) [reference number in manuscript]	Countries of Included Studies	Health Conditions in Included Studies	Intervention Characteristics	Control Group Treatments	Outcomes Measured
				• Antidepressant medication switch without app<br>• No CBT or interpersonal therapy<br>• Standard of care<br>• Computer-based CBT	• Experiential avoidance<br>• Self-esteem<br>• Adherence to medication<br>• Wellbeing<br>• Rumination<br>• Worrying<br>• Recovery<br>• Empowerment<br>• Psychosocial functioning<br>• Behavioural activation<br>• Severity of depressive symptoms
Hou (2016) [24]	• Italy<br>• England<br>• Spain<br>• Australia<br>• US<br>• Asia<br>• Africa	• T1DM<br>• T2DM	• Carbohydrate/insulin bolus calculator<br>• Medication adjustment support<br>• Graphical feedback<br>• HCP feedback<br>• Glycemic target setting<br>• BG target setting<br>• General education system<br>• Reminders	• Standard education<br>• Usual care<br>• Standard medical care<br>• Self-care management<br>• Conventional therapy<br>• Care from the centre	• HbA1c
Hou (2018) [25]	• Finland<br>• Norway<br>• US<br>• Japan<br>• UK<br>• Korea<br>• China	• T1DM<br>• T2DM	• Carbohydrate/insulin bolus calculator<br>• Medication adjustment support<br>• Real-time personalized feedback<br>• HCP feedback	• Standard medical care<br>• Usual care<br>• Self-management<br>• Care from the centre	• HbA1c

First Author (Year) [reference number in manuscript]	Countries of Included Studies	Health Conditions in Included Studies	Intervention Characteristics	Control Group Treatments	Outcomes Measured
	• Canada<br>• Saudi Arabia<br>• Singapore<br>• Poland<br>• India		• Glycemic target setting<br>• BG target setting<br>• General education system<br>• Reminders<br>• Food exchange<br>• Plasma glucose targets	• Health coach support<br>• Logbooks<br>• Written instructions	
Hou (2022) [62]	• Netherlands<br>• China<br>• Brazil<br>• Sweden	• Stress urinary incontinence	• Instructions on pelvic floor muscle training<br>• Reminders<br>• Education<br>• Feedback	• Regular care<br>• Pelvic floor rehabilitation education through lectures<br>• Waiting list<br>• Printed instructions	• Severity of symptoms<br>• QoL<br>• Adherence<br>• Self-reported adherence
Hrynyschyn (2021) [53]	• US<br>• Sweden<br>• Australia<br>• South Korea<br>• Germany	• Mild to moderate depression or depressive symptoms	• Mood managing<br>• Self-esteem and acceptance<br>• Behavioral activation<br>• Cognitive restructuring<br>• Self-monitoring<br>• Mood and anxious feelings report<br>• Self-help	• Waitlist<br>• Face to face behavioral activation sessions<br>• Health tips<br>• Another app<br>• Treatment as usual	• Depressive symptoms<br>• Anxiety<br>• Self-efficacy/self-esteem<br>• QoL
Hyun (2021) [26]	• US<br>• UK<br>• Finland<br>• Canada<br>• Malaysia<br>• Korea	• T2DM	• Key indicators recording<br>• Educational messages<br>• Real time graphical feedback<br>• Reminders<br>• Advice	N/A	• HbA1c/ FBG/ hypoglycemia frequency

First Author (Year) [reference number in manuscript]	Countries of Included Studies	Health Conditions in Included Studies	Intervention Characteristics	Control Group Treatments	Outcomes Measured
	• China<br>• India<br>• Saudi Arabia<br>• Indonesia<br>• Norway		• Patient community<br>• Communication with clinicians<br>• Goal setting<br>• Information look-up		
Karatas (2022) [54]	• Denmark<br>• Switzerland<br>• Israel<br>• Netherlands<br>• China<br>• US<br>• Thailand	• Obesity<br>• Asthma<br>• T1DM<br>• Congenital HD<br>• ADHD	• Goals<br>• Diary<br>• Tips<br>• Messaging/ chat function<br>• Feedback<br>• Medication reminder<br>• Education<br>• Communication with health professionals<br>• Records<br>• Monitoring<br>• Personalized exercise planning tools	• Usual care	• Asthma control: frequency of asthma attacks, respiratory infection and antibiotic use days, and medication adherence<br>• Mean peak oxygen uptake<br>• HbA1c<br>• BMI<br>• ADHD: pill counts and clinician rating scores<br>• Health behaviour changes<br>• QoL<br>• Psychosocial outcomes
Kassavou (2022) [27]	• US<br>• Australia<br>• Canada<br>• China<br>• New Zealand<br>• Ghana<br>• India	• Hypertension	• Support changes in both blood pressure and related health behaviors	• Text messages<br>• Video clips<br>• Counselling<br>• No additional advice<br>• Usual care<br>• Remote BP measurement<br>• Tailored exercise program	• SBP/DBP<br>• Medication adherence<br>• Physical activity<br>• Healthy diet<br>• Smoking and alcohol

First Author (Year) [reference number in manuscript]	Countries of Included Studies	Health Conditions in Included Studies	Intervention Characteristics	Control Group Treatments	Outcomes Measured
				• Enhanced usual care	
Kim (2022) [28]	• US<br>• Australia<br>• Denmark<br>• UK<br>• South Korea<br>• Sweden<br>• Japan	• Schizophrenia<br>• Bipolar disorder<br>• Depressive disorder or major depression	• Self-assessment services<br>• Monitoring disease-related conditions and daily activities<br>• Education<br>• Feedback providing<br>• Notification with auditory and visual signals to encourage engagement and data tracking	• Treatment as usual<br>• Clinic-based group intervention<br>• Paper and pencil condition<br>• Standardized treatment<br>• Daily mood diary<br>• Monitoring of daily activities<br>• Standard care<br>• Antidepressant switch<br>• Waitlist<br>• Computer intervention	• Depression symptoms<br>• Mania symptoms<br>• Positive/negative psychotic symptoms<br>• Adverse event

First Author (Year) [reference number in manuscript]	Countries of Included Studies	Health Conditions in Included Studies	Intervention Characteristics	Control Group Treatments	Outcomes Measured
Lee (2018) [55]	• UK<br>• Norway<br>• Spain<br>• Finland<br>• Turkey<br>• US<br>• Sweden<br>• Belgium	• Chemotherapy related toxicity in cancer patients<br>• Chronic pain or fibromyalgia<br>• FMS<br>• HF<br>• Allergic rhinitis<br>• Asthma<br>• Spina bifida<br>• CVD<br>• PD<br>• Lung transplant recipients	• Tailor self-care advice<br>• Self-management strategies<br>• Self-record<br>• Feedback<br>• Reminder<br>• Lifestyle information<br>• Step count<br>• Encouragement	N/A	• Physical functioning<br>• Adherence to prescribed medication<br>• Ease of symptoms<br>• Fatigue<br>• Hand-foot syndrome<br>• Pain management<br>• QoL<br>• Self-care/self-monitoring<br>• HF-related hospital days<br>• Balance in gait<br>• Physical activity<br>• Medication adherence<br>• Caregiver communication<br>• Abnormal health indicators<br>• PD functional scale
Leme Nagib (2020) [56]	• Sweden<br>• Brazil	• Urinary incontinence	• Information about SUI<br>• Instructions of PFMT<br>• Statistics about patient's training<br>• Urotherapist support<br>• Reminder about PFMT and the exercise	• Post treatment plan<br>• Printed instructions	• Urinary incontinence<br>• QoL<br>• Impression of improvement<br>• Treatment adherence

First Author (Year) [reference number in manuscript]	Countries of Included Studies	Health Conditions in Included Studies	Intervention Characteristics	Control Group Treatments	Outcomes Measured
Liu (2020) [29]	• Mexico<br>• US<br>• Denmark<br>• Norway<br>• Finland<br>• India<br>• UK<br>• China<br>• Congo<br>• Japan<br>• Canada<br>• Ghana<br>• Hong Kong<br>• Korea	• T2DM<br>• Hypertension	• Data logging<br>• Personalized feedback<br>• Communication with hcps<br>• Education materials<br>• Data visualization	• Usual care<br>• Education<br>• Fitbit<br>• HCP counseling	• HbA1c/ FBG<br>• SBP/ DBP<br>• Waist circumference/ body weight/ BMI<br>• Total cholesterol/ LDL-c/ HDL-c/ TG
Lu (2022) [43]	• Worldwide<br>• US<br>• Germany<br>• Sweden<br>• Australia<br>• Japan<br>• Korea<br>• Switzerland<br>• Taiwan<br>• UK	• Anxiety<br>• Depression<br>• Both	• CBT<br>• Gaming<br>• Regulating emotions through mindfulness<br>• Acceptance<br>• Self-soothing activities<br>• Problem solving	• Waitlist<br>• Bibliotherapy<br>• Education<br>• Usual care<br>• Face-to-face behavior activation therapy	• Anxiety symptom management<br>• Depressive symptom management
Lunde (2018) [30]	• Norway<br>• Sweden<br>• Finland<br>• US<br>• Japan	• T1DM<br>• T2DM<br>• MI<br>• Heart disease	• Registration of lifestyle factors<br>• Exercise and/or physical activity<br>• Dietary habits<br>• Self-monitoring	• Usual care<br>• Simplified app with drug adherence e-diary<br>• Health coaching	• HbA1c

First Author (Year) [reference number in manuscript]	Countries of Included Studies	Health Conditions in Included Studies	Intervention Characteristics	Control Group Treatments	Outcomes Measured
	• Canada<br>• China		• Health parameters monitoring<br>• Health coaching		
Marcano Belisario (2013) [57]	• Taiwan<br>• UK	• Asthma	• Symptoms and medication recording<br>• Self-management feedback	• Paper-based self-management	• Asthma symptom scores<br>• Unscheduled visits to emergency department<br>• Hospital admission<br>• GP consultations for asthma<br>• Unscheduled general practice nurse consultation<br>• Out of hours attendances<br>• HRQoL<br>• Adherence to intervention<br>• Healthcare costs<br>• Lung function<br>• Adverse event
Mikulski (2021) [31]	• US<br>• China<br>• Spain<br>• Ghana	• Arterial hypertension	• Logbooks of BP and lifestyle<br>• Reports and feedback on recorded data<br>• Educational and instructional content<br>• Encouragement messages<br>• Communication with health professionals, caregivers and peers	N/A	• Medication adherence

First Author (Year) [reference number in manuscript]	Countries of Included Studies	Health Conditions in Included Studies	Intervention Characteristics	Control Group Treatments	Outcomes Measured
Moon (2019) [32]	• Australia<br>• US<br>• Ireland<br>• UK (Scotland)	• Autism Spectrum Disorder	• Communication and social skills improvement<br>• Behavioral change	• Delayed intervention<br>• Usual care	• MSEL: Visual reception, fine motor, receptive language, fine expressive<br>• MCDI: word produced, gestures<br>• CSBS: social communication, speech, symbolic
Moreno-Ligero (2023a) [44]	• Indonesia<br>• Spain<br>• Australia<br>• US<br>• Germany<br>• Singapore<br>• Belgium<br>• Israel<br>• South Korea	• Stroke<br>• PD<br>• Neurological conditions<br>• Multiple sclerosis	• Gait training<br>• Balance training or home-based physical activity programs<br>• Speed-Interactive Treadmill Training<br>• Monitoring of physical activity and health outcomes<br>• Feedback<br>• Information<br>• Music-motor training<br>• Core stability training<br>• Balance disc training	• Conventional physical and occupational therapies<br>• Electrical stimulation therapy<br>• Conventional Speed-Interactive Treadmill Training<br>• Conventional gait training<br>• Paper-based physical activity intervention<br>• Information leaflet<br>• Phone calls<br>• Usual care	• Gait spatiotemporal parameters (speed, cadence, stride length, affected step length, and non-affected step length)<br>• Dynamic balance

First Author (Year) [reference number in manuscript]	Countries of Included Studies	Health Conditions in Included Studies	Intervention Characteristics	Control Group Treatments	Outcomes Measured
Moreno-Ligero (2023b) [60]	• Asia<br>• Europe<br>• North and South America<br>• Oceania	• Ostearthritis<br>• Chronic low back pain<br>• Chronic musculoskeletal pain<br>• Chronic neck pain<br>• Chronic pelvic pain<br>• Interstitial cystitis/bladder pain syndrome<br>• Irritable bowel syndrome<br>• Fibromyalgia<br>• Unspecified chronic pain	• Home-based physical activity<br>• Education<br>• CBT<br>• Monitoring pain-related outcomes and symptoms<br>• Monitoring physical activity parameters<br>• Mind relaxation techniques<br>• Motivational reminders<br>• Nutrition information<br>• Feedback	• Usual care<br>• Waiting list<br>• Written instructions about postural hygiene<br>• Physical activity information booklet and advice to stay active<br>• Online standard dietary education materials<br>• Rest<br>• Physiotherapy	• Pain intensity<br>• QoL<br>• Functional disability
Özden (2023a) [45]	N/A	• PD	• Education<br>• Support<br>• Sensor-based training<br>• Reminders<br>• Exercise prescription	• Face to face rehabilitation<br>• Regular rehabilitation/exercise program<br>• Motor symptom diary<br>• Telephone-based education	• Disease/symptom severity<br>• Drug adherence<br>• QoL<br>• Function (balance and gait kinematics)<br>• Aerobic capacity<br>• Cognition and psychology

First Author (Year) [reference number in manuscript]	Countries of Included Studies	Health Conditions in Included Studies	Intervention Characteristics	Control Group Treatments	Outcomes Measured
Özden (2023b) [46]	N/A	• Post-operative knee aristoplasty	• Education<br>• Monitoring<br>• Exercise instructions	• Face to face rehabilitation<br>• Formal physiotherapy<br>• Basic information	• Function<br>• Pain<br>• Range of motion
Park (2020) [33]	N/A	• Depression/ depressive symptoms	• Cognitive training<br>• Mood monitoring<br>• Gaming or interactive<br>• In-app feedback<br>• Reminders or prompts<br>• HCP support	• Another control app<br>• Information control<br>• Pen-paper mood monitoring<br>• Regular smartphone use<br>• Waitlist control<br>• Face-to-face BA sessions<br>• Pharmacotherapy<br>• Computerized CBT	• Depressive symptoms
Peng (2020) [34]	• Austria<br>• Spain<br>• US<br>• China<br>• India<br>• Australia<br>• Ghana<br>• Denmark	• Diabetes<br>• T2DM<br>• Hypertension<br>• Lung transplant recipients<br>• Atrial fibrillation<br>• AIDS<br>• Stroke<br>• Chronic disease<br>• CVD	• Documentation<br>• Education<br>• Medication reminder<br>• Appointment reminder<br>• Data sharing<br>• Feedback message<br>• Customization<br>• Clinical decision support<br>• Data statistics	• Usual care	• Medication adherence

First Author (Year) [reference number in manuscript]	Countries of Included Studies	Health Conditions in Included Studies	Intervention Characteristics	Control Group Treatments	Outcomes Measured
		• PCI<br>• Psoriasis			
Pi (2023) [47]	• Canada<br>• Germany<br>• Denmark<br>• Greece<br>• US<br>• Switzerland<br>• Italy<br>• China	• T1DM	• Calculating carbohydrate content or insulin bolus<br>• Collecting biodata<br>• Tracking patterns or trends in diabetes management<br>• Self-monitoring<br>• Diabetes education<br>• Social support	• Usual care<br>• Routine carbohydrate count<br>• Traditional glucose meter	• HbA1c
Seegan (2023) [49]	N/A	• Anxiety<br>• Depression<br>• Both	• Coaching<br>• Feedback<br>• Reminders	• Treatment as usual<br>• Waitlist	• Anxiety symptom severity<br>• Depression symptom severity
Shaw (2020) [35]	• Taiwan<br>• UK<br>• Hong Kong<br>• US<br>• Netherlands<br>• Belgium<br>• Greece<br>• Switzerland<br>• China	• COPD	• Data recording and monitoring<br>• Feedback/ alerts<br>• Education<br>• Self-help strategies<br>• Exercises	• Usual care<br>• Self-management education<br>• Regular phone call	• Frequency of COPD exacerbations<br>• Physical function<br>• QoL<br>• Dyspnea<br>• Fatigue<br>• Physical activity<br>• Self-efficacy<br>• Anxiety and depression
Thompson (2023) [48]	N/A	• Osteoarthritis<br>• Low back pain	• Training<br>• Enablement	• Same therapeutic exercise	• Pain intensity<br>• Pain interference

First Author (Year) [reference number in manuscript]	Countries of Included Studies	Health Conditions in Included Studies	Intervention Characteristics	Control Group Treatments	Outcomes Measured
		• Ankle sprains<br>• Frozen shoulder<br>• Neck pain<br>• Wrist, hand and finger injuries	• Environmental restructuring<br>• Education<br>• Persuasion and incentivization	programs without the use of a mobile app with similar tailored therapeutic exercise or physical activity intervention	• Self-reported physical function<br>• Physical performance<br>• Psychosocial outcomes<br>• QoL
Whitehead (2016) [58]	• Europe<br>• Oceania<br>• Asia<br>• US	• T1DM<br>• T2DM<br>• CVD<br>• Chronic lung disease	• Data recording<br>• Feedback<br>• Self-management	• Standard care<br>• Offline self-management<br>• Clinic visit<br>• Usual care	• HbA1c<br>• 6-minute walking test<br>• Lung function parameters
Wickersham (2019) [59]	• US	• Subthreshold or full PTSD	• Psychoeducation<br>• Self-assessment<br>• Symptoms management tools<br>• Links to resources for individuals with PTSD<br>• Dyadic intervention program for veterans and their partners (for psychological, social, and physical support)<br>• Social engagement<br>• Relaxation	• Waitlist control<br>• App access without daily direction<br>• Clinician-supported PTSD coach	• PTSD symptoms<br>• Stress<br>• Anxiety<br>• Depression<br>• Self-compassion<br>• Sleep quality<br>• Pain<br>• Psychosocial functioning<br>• QoL

First Author (Year) [reference number in manuscript]	Countries of Included Studies	Health Conditions in Included Studies	Intervention Characteristics	Control Group Treatments	Outcomes Measured
Wu (2017) [37]	• US<br>• UK<br>• Netherlands<br>• Norway<br>• Japan<br>• Australia<br>• Italy<br>• France<br>• Korea	• T1DM<br>• T2DM	• Monitoring<br>• Medication management<br>• Lifestyle modification<br>• Complication prevention<br>• Psychological care	• Standard face to face care<br>• Standard care<br>• Standard paper diary/logbook<br>• Usual care<br>• Self-care regimen<br>• Standard carbohydrate counting<br>• Conventional clinic visits<br>• Self-monitoring blood glucose	• HbA1c<br>• Adverse events
Wu (2019) [36]	• Europe<br>• Australia<br>• North America<br>• Asia<br>• US<br>• China	• T1DM<br>• T2DM<br>• Prediabetes<br>• Gestational diabetes	• Health information and education<br>• Medication adjustment support<br>• Insulin bolus calculator<br>• Clinical measurements logging<br>• Feedback	• No/usual care<br>• Standard medical care<br>• Another smartphone app	• HbA1c
Xu (2020) [38]	N/A	• Hypertension	• BP recording<br>• Medication reminder<br>• Abnormal values warning<br>• Patient education<br>• Health recommendation<br>• Encouragement	• No/usual care<br>• Self-measured BP<br>• Another mobile app<br>• Regular routine education	• SDP/DBP<br>• Medication adherence<br>• Physical activity

First Author (Year) [reference number in manuscript]	Countries of Included Studies	Health Conditions in Included Studies	Intervention Characteristics	Control Group Treatments	Outcomes Measured
Yang (2018) [39]	• China<br>• Germany<br>• Spain<br>• Italy<br>• Netherlands	• COPD	• Physiological status or health behavior monitoring<br>• Follow up and personalize feedback<br>• Self-monitoring<br>• Coaching	• Usual care	• Hospital admission<br>• Average days of hospital stay<br>• Exercise capacity and activity levels<br>• Lung function

Legend: ADHD: attention deficit hyperactivity disorder; AIDS: acquired immune deficiency syndrome; BG: blood glucose; BMI: body mass index; BP: blood pressure; CBT: cognitive behavioural therapy; CHD: coronary heart disease; COPD: chronic obstructive pulmonary disease; CSBS: communication and Symbolic Behaviour Scales; CVD: cardiovascular disease; DBP: diastolic blood pressure; FBG: fasting blood glucose; FMS: fibromyalgia syndrome; GP: general practitioner; HbA1c: glycated hemoglobin; HCP: health-care professional; HD: heart disease; HDL-c: high-density lipoprotein; HF: heart failure; HRQoL: health-related quality of life; LDL-c: low-density lipoprotein; MCDI: MacCarthur-Bates Communication Development Inventory; MI: myocardial infarction; MSEL: Mullen Scales of Early Learning; N/A: The information was not available in the text; PCI: percutaneous coronary intervention; PD: Parkinson's disease; PFMT: pelvic floor muscle training; PTSD: post-traumatic stress disorder; QoL: quality of life; SBP: systolic blood pressure; SUI: stress urinary incontinence; T1DM: type 1 diabetes mellitus; T2DM: type 2 diabetes mellitus; TC: total cholesterol; TG: triglyceride; UK: United Kingdom; US: United States

Supplementary Table 5. Details on review population characteristics of 48 included reviews on the effectiveness of app-based health interventions

First Author (Year) [reference number in manuscript]	Sample Size				Mean Age		
	Total	Minimum	Maximum	Mean (Median)	Minimum	Maximum	Mean
Al-Arkee (2021) [16]	2018	24	412	126 (86)	46.5	73.8	-
Amalindah (2020) [50]	1902	29	330	158.5 (136)	14	80	-
Armitage (2020) [17]	1194	24	412	- (102)	20.3	73.8	56.7
Bonoto (2017) [18]	1263	17	180	93 (72)	12.9	62.3	44.3
Cai (2020) [19]	2129	-	-	-	51.1	66.1	58.4
Chew (2022) [41]	2870	40	750	179 (114)	22.7	70.1	45
Chew (2023) [40]	2478	28	502	177 (99)	20	55	39.1
Cui (2016) [20]	1022	24	180	130 (117)	45.2	66.6	57.1
Davergne (2023) [42]	1030	20	305	103 (52)	11.6	79.3	46.82
Didyk (2021) [51]	2100	8	1245	349 (97)	40.63	48.66	42.6
DiFilippo (2015) [52]	282	58	128	94 (96)	18	63	42
El-Gayar (2021) [21]	1920	28	445	109 (86)	32.9	68.1	51.2
Enricho Nkhoma (2021) [22]	696	41	247	125 (98)	48.4	69.6	58.9
Han (2020) [15]	2965	-	-	-	25	76	-
He (2021) [23]	2585	54	247	135.6 (120)	31.7	68	52.7
Hernández-Gómez (2022) [61]	651	34	164	93 (93)	23.71	43.95	35.49
Hou (2016) [24]	509	53	130	107 (122.5)	34	36	35.7

Hou (2018) [25]	572	8	173	143 (95)	33.82	36.33	35
Hou (2022) [62]	884	33	262	147 (116)	18	86	44.28
Hrynyschyn (2021) [53]	1534	30	626	341 (432)	23.71	44.21	35.68
Hyun (2021) [26]	1608	14	250	114.0 (94.5)	48.8	68.0	57.04
Karatas (2022) [54]	755	39	234	107 (77)	6	18	-
Kassavou (2022) [27]	7415	-	-	-	18	-	-
Kim (2022) [28]	1307	34	170	93 (87)	18.5	49.65	35.87
Lee (2018) [55]	1415	28	372	117 (88)	30	75	-
Leme Nagib (2020) [56]	406	33	250	135.3 (123)	18	72	47.9
Liu (2020) [29]	2285	14	250	- (75)	48.4	69.5	57.3
Lu (2022) [43]	1942	30	283	115.7 (90)	19.8	56.5	35.5
Lunde (2018) [30]	1375	30	519	152.8 (131)	-	-	-
Marcano Belisario (2013) [57]	408	120	288	204 (204)	-	-	-
Mikulski (2021) [31]	-	-	-	443 (-)	44	60	-
Moon (2019) [32]	328	28	75	46 (45)	3	10	4.5
Moreno-Ligero (2023a) [44]	528	18	124	40.6 (38)	51.1	65.3	60.4
Moreno-Ligero (2023b) [60]	2641	8	597	120 (75)	22.8	70.2	38.93
Özden (2023a) [45]	-	-	-	-	59.3	67.7	-
Özden (2023b) [46]	-	-	-	-	>18	65.9	64.4
Park (2020) [33]	2956	25	626	173 (93)	18	48	34.75
Peng (2020) [34]	1785	24	411	127.5 (128)	47.3	71.9	58.1
Pi (2023) [47]	779	32	168	86.56 (80)	12.65	17.8	13.13

Seegan (2023) [49]	7669	20	1029	178 (120)	14.82	46	26.20
Shaw (2020) [35]	1447	23	343	111 (79)	58.37	72.9	67.18
Thompson (2023) [48]	845	20	220	84.5 (79)	27	63	46.21
Whitehead (2016) [58]	1139	48	288	126 (94)	33.8	72.1	51.69
Wickersham (2019) [59]	653	20	320	130.6 (120)	32	46	38.8
Wu (2017) [37]	974	30	137	89 (91)	-	-	-
Wu (2019) [36]	2526	13	498	109.83 (71)	32.44	66.29	50.09
Xu (2020) [38]	1607	50	443	200 (129)	-	-	-
Yang (2018) [39]	391	24	99	48 (44)	63.5	81	71.15

Supplementary Table 6. Summary of outcomes, effect estimates and conclusions of 48 included reviews on the effectiveness of app-based health interventions

First Author (Year) [reference number in manuscript]	Narratively or Quantitatively Reported Outcomes [a]	No. of Studies	Definition a Primary or Secondary Outcome	If Meta-Analysis was Conducted				Author's Conclusion
				Random/Fixed Effect Model	Effect Measure	Effect Estimate	(95% CI)	
Al-Arkee (2021) [16]	Medication adherence (MMAS)	6	Primary	Random	MD	0.9	(0.03, 1.78)	Better health-related outcomes
	SBP (mm Hg)	3	Secondary	Random	MD	-3.11	(-7.59, 1.37)	
	DBP (mm Hg)	2	Secondary	Random	MD	-3.13	(-6.40, 0.15)	
	LDL-C (mg/dL)	2	Secondary	Random	MD	-0.46	(-0.79, -0.12)	
	TC (mg/dL)	2	Secondary	Random	MD	-0.38	(-1.14, 0.38)	
	PAM	2	Secondary	N/A	N/A	N/A	N/A	
	CVD knowledge	2	Secondary	N/A	N/A	N/A	N/A	
	QoL	2	Secondary	N/A	N/A	N/A	N/A	
	HbA1c	2	Secondary	N/A	N/A	N/A	N/A	
Amalindah (2020) [50]	HbA1c	12	Primary	N/A	N/A	N/A	N/A	Better health-related outcomes
	LDL-c	2	Primary	N/A	N/A	N/A	N/A	
	HDL	2	Primary	N/A	N/A	N/A	N/A	
	BMI	3	Primary	N/A	N/A	N/A	N/A	
	Hypoglycemia effect	3	Primary	N/A	N/A	N/A	N/A	
	Psychological aspects: self-efficacy, depression, quality of life, knowledge, and adherence	4	Primary	N/A	N/A	N/A	N/A	
Armitage (2020) [17]	Medication adherence (MMAS)	9	Primary	Random	OR	2.12	(1.635, 2.747)	Better health-related outcomes
Bonoto (2017) [18]	HbA1c	13	Primary	Random	MD	-0.44	(-0.59, -0.29)	Better health-related outcomes
	FBG	4	Secondary	Joint Analysis	MD	0.05	(-1.39, 1.49)	

First Author (Year) [reference number in manuscript]	Narratively or Quantitatively Reported Outcomes [a]	No. of Studies	Definition a Primary or Secondary Outcome	If Meta-Analysis was Conducted				Author's Conclusion
				Random/Fixed Effect Model	Effect Measure	Effect Estimate	(95% CI)	
	Body weight	4	Secondary	Joint Analysis	MD	-0.39	(-1.43, 0.66)	
	SBP	4	Secondary	Joint Analysis	MD	0.1	(-2.36, 2.55)	
	DBP	4	Secondary	Joint Analysis	MD	0.37	(-1.10, 1.85)	
	TC	3	Secondary	Joint Analysis	MD	−3.44	(-12.87, 6.00)	
	HDL-C	3	Secondary	Joint Analysis	MD	-2.15	(-5.40, 1.10)	
	LDL-C	3	Secondary	Joint Analysis	MD	1.69	(-5.67, 9.06)	
	TG	3	Secondary	Joint Analysis	MD	-14.67	(-33.40, 4.06)	
Cai (2020) [19]	Body weight (kg)	9	Primary	Random	WMD	-0.84	(-1.51, -0.17)	Better health-related outcomes
	BMI (kg/m^2)	9	Secondary	Random	WMD	-0.08	(-0.41, 0.25)	
	Waist circumference (cm)	5	Secondary	Random	WMD	-1.35	(-2.16, -0.55)	
	Fat mass	1	Secondary	N/A	N/A	N/A	N/A	
	Percentage of body fat	1	Secondary	N/A	N/A	N/A	N/A	
Chew (2022) [41]	Weight loss (kg) (<3 months)	8	N/A	Random	WMD	-1.15	(-3.02, -0.72)	No improvement in health-related outcomes
	Weight loss (kg) (3 months)	11	N/A	Random	WMD	-2.18	(-3.59, -0.78)	
	Weight loss (kg) (6 months)	13	N/A	Random	WMD	-2.15	(-3.25, -1.05)	
	Weight loss (kg) (9 to 12 months)	5	N/A	Random	WMD	-1.63	(-2.99, -0.26)	
	Waist circumference (cm) (<3 months)	4	N/A	Random	WMD	-2.3	(-6.98, 2.38)	
	Waist circumference (cm) (3 months)	4	N/A	Random	WMD	-3.85	(-9.31, 1.60)	
	Waist circumference (cm) (6 months)	2	N/A	Random	WMD	−0.92	(-3.88, 2.04)	
	Waist circumference (cm) (12 months)	3	N/A	Random	WMD	−1.19	(-3.80, 1.43)	

First Author (Year) [reference number in manuscript]	Narratively or Quantitatively Reported Outcomes [a]	No. of Studies	Definition a Primary or Secondary Outcome	If Meta-Analysis was Conducted				Author's Conclusion
				Random/Fixed Effect Model	Effect Measure	Effect Estimate	(95% CI)	
	HDL-c (mg/dL) (3 months)	2	N/A	Random	WMD	0.01	(-0.15, 0.17)	
	LDL-c (mg/dL) (3 months)	2	N/A	Random	WMD	−0.06	(-1.31, 1.44)	
	HbA1c (%) (3-6 months)	3	N/A	Random	WMD	−0.22	(-1.03, -0.6)	
	Total energy intake per day (6-12 months)	3	N/A	Random	WMD	−86.2	(-494.53, 322.12)	
	SBP (mm Hg) (3 months)	3	N/A	Random	WMD	−4.67	(-5.95, -3.40)	
	SBP (mm Hg) (6 months	2	N/A	Random	WMD	−0.28	(-15.6, 15.03)	
	DBP (mm Hg) - 3 months	3	N/A	Random	WMD	−2.88	(-8.37, 2.62)	
	DBP (mm Hg) - 6 months	2	N/A	Random	WMD	−0.65	(-1.56, 0.26)	
Chew (2023) [40]	Weight loss (kg)	12	Primary	Random	MD	−2.15	(-3.17, -1.12)	Better health-related outcomes
	BMI (kg/m^2)	3	Primary	Random	MD	−0.82	(-2.03, 0.39)	
	Waist circumference (cm)	11	Primary	Random	MD	−2.48	(-3.51, -1.44)	
	SBP (mm Hg)	6	Secondary	Random	MD	−0.83	(-3.21, 1.54)	
	DBP (mm Hg)	6	Secondary	Random	MD	−0.89	(-2.75, 0.96)	
	Body fat (%/kg)	3	Secondary	Random	SMD	−0.36	(-1.90, 1.18)	
	Total cholesterol (mg/dL)	2	Secondary	Random	MD	2.82	(-2.86, 8.51)	
	LDL-C (mg/dL)	2	Secondary	Random	MD	−3.78	(-10.05, 2.49)	
	HDL-C (mg/dL)	2	Secondary	Random	MD	1.63	(-16.00, 19.25)	
	Triglyceride (mg/dL)	4	Secondary	Random	MD	−0.22	(-0.33, 0.11)	
	HbA1c (%)	4	Secondary	Random	MD	−0.12	(-0.21, -0.02)	

First Author (Year) [reference number in manuscript]	Narratively or Quantitatively Reported Outcomes [a]	No. of Studies	Definition a Primary or Secondary Outcome	If Meta-Analysis was Conducted				Author's Conclusion
				Random/Fixed Effect Model	Effect Measure	Effect Estimate	(95% CI)	
	Total calorie consumption/day (kcal)	5	Secondary	Random	MD	−128.30	(-182.67, -73.94)	
	Physical activity	6	Secondary	Random	SMD	−0.11	(-0.87, 0.65)	
Cui (2016) [20]	HbA1c (%) with feedback group	6	Main and subgroup for primary	Random	MD	-0.4	(-0.69, -0.11)	Slightly better health-related outcomes
	HbA1c (%) without feedback group	N/A	Subgroup for primary	Random	MD	-0.46	(-1.19, 0.26)	
	SBP (mm Hg)	4	Secondary	Random	MD	-2.62	(-5.6, 0.36)	
	DBP (mm Hg)	4	Secondary	Random	MD	-1.76	(-3.6, 0.07)	
	LDL-C	3	Secondary	Random	MD	-0.12	(-0.34, 0.11)	
	HDL-C	3	Secondary	Random	MD	0.01	(-.05, 0.07)	
	TG	3	Secondary	Random	MD	-0.06	(0.32, 0.19)	
	TC	3	Secondary	Random	MD	-0.15	(-0.6, 0.3)	
	Body weight (kg)	4	Secondary	Random	MD	-0.84	(-2.04, 0.36)	
	Physical activity (minutes)	1	Secondary	Random	MD	10.59	(4.94, 16.25)	
	Medication use changes	2	Secondary	N/A	N/A	N/A	N/A	
Davergne (2023) [42]	Physical function	8	N/A	Random	SMD	0.35	(0.51, 0.19)	Slightly better health-related outcomes
	QoL	3	N/A	Random	SMD	0.42	(-0.09, 0.93)	
	Adherence	6	N/A	N/A	N/A	N/A	N/A	
	Self-confidence	2	N/A	Random	MD	0.67	(0.37, 0.96)	
	Health care consumption	3	N/A	N/A	N/A	N/A	N/A	
	Adverse events	7	N/A	N/A	Risk ratio	0.68	(0.37, 1.23)	
Didyk (2021) [51]	Pain intensity	6	Primary	N/A	N/A	N/A	N/A	
	Disability/function	3	Primary	N/A	N/A	N/A	N/A	

First Author (Year) [reference number in manuscript]	Narratively or Quantitatively Reported Outcomes [a]	No. of Studies	Definition a Primary or Secondary Outcome	If Meta-Analysis was Conducted				Author's Conclusion
				Random/Fixed Effect Model	Effect Measure	Effect Estimate	(95% CI)	
	Pain self-efficacy	1	Primary	N/A	N/A	N/A	N/A	No improvement in health-related outcomes
	Health-related QoL	1	Primary	N/A	N/A	N/A	N/A	
	Adherence to home exercise	6	Primary	N/A	N/A	N/A	N/A	
DiFilippo (2015) [52]	Weight loss (kg)	3	Primary (study 1)	N/A	N/A	N/A	N/A	Better health-related outcomes
			Secondary (study 2)	N/A	Mean change (kg)	-3.3	(-5.4, 1.2)	
			Primary (study 3)	N/A	N/A	N/A	N/A	
	Perceived motivation	1	Secondary	N/A	N/A	N/A	N/A	
	Positive affect	1	Secondary	N/A	N/A	N/A	N/A	
	Adherence to diet monitoring	1	Secondary	N/A	N/A	N/A	N/A	
El-Gayar (2021) [21]	HbA1c (%)	24	Primary	Random	MD	-0.38	(-0.50, -0.26)	Better health-related outcomes
Enricho Nkhoma (2021) [22]	Medication adherence	3	Primary	Fixed	SMD	0.393	(0.17, 0.61)	Slightly better health-related outcomes
	Patient adherence	N/A	Primary	Fixed	SMD	0.632	(0.17, 0.61)	
	HbA1c	4	Secondary	Fixed	MD	-0.314	(-0.477, -0.151)	
	BMI	4	Secondary	Fixed	MD	-0.28	(-0.545, -0.015)	
	BP	N/A	Secondary	N/A	N/A	N/A	N/A	
	Cholesterol	N/A	Secondary	N/A	N/A	N/A	N/A	
Han (2020) [15]	SBP (mm Hg)	8	Primary	Random	MD	-8.12	(-11.47, -4.77)	Better health-related outcomes
	DBP (mm Hg)	18	Primary	Random	MD	-6.67	(-8.92, -4.41)	
He (2021) [23]	HbA1c pooled (%)	_	Primary	Random	MD	-0.45	(-0.58, -0.32)	Better health-related outcomes
	HbA1c 3 months	10	Primary	Random	MD	-0.41	(-0.57, -0.24)	
	HbA1c 3-6 months	12	Primary	Random	MD	-0.59	(-0.94, -0.25)	
	HbA1c 6-12 months	4	Primary	Random	MD	-0.47	(-1.06, 0.12)	

First Author (Year) [reference number in manuscript]	Narratively or Quantitatively Reported Outcomes [a]	No. of Studies	Definition a Primary or Secondary Outcome	If Meta-Analysis was Conducted				Author's Conclusion
				Random/Fixed Effect Model	Effect Measure	Effect Estimate	(95% CI)	
	Self-management pooled	11	Secondary	Random	SMD	0.73	(0.37, 1.09)	
	Self-management performance	3	Secondary	Random	SMD	3.03	(-0.35, 6.40)	
	Diet management	5	Secondary	Random	SMD	0.37	(-0.19, 0.93)	
	Exercise engagement	6	Secondary	Random	SMD	0.35	(-0.05, 0.75)	
	Medication adherence	2	Secondary	Random	SMD	0.8	(0.15, 1.46)	
	Blood glucose self-monitoring	4	Secondary	Random	SMD	0.83	(-0.17, 1.83)	
	Foot care	5	Secondary	Random	SMD	0.25	(-0.26, 0.76)	
	Psychological wellbeing pooled	6	Secondary	Random	SMD	-0.17	(-0.37, 0.03)	
	Depression	4	Secondary	Random	SMD	-0.22	(-0.61, 0.17)	
	Distress	4	Secondary	Random	SMD	-0.11	(-0.29, 0.07)	
	HDL-c (mmol/L)	6	Secondary	Fixed	MD	0.04	(0.00, 0.08)	
	SBP	7	Secondary	Random	MD	0.08	(-3.02, 3.17)	
	DBP	6	Secondary	Fixed	MD	-0.81	(-2.16, 0.53)	
	BMI	9	Secondary	Fixed	MD	-0.06	(-0.54, 0.42)	
	TC	6	Secondary	Fixed	MD	-0.1	(-0.22, 0.02)	
	LDL	7	Secondary	Fixed	MD	0.02	(-0.07, 0.12)	
	TG	7	Secondary	Fixed	MD	-0.11	(-0.26, 0.04)	
	QoL pooled	7	Secondary	Random	MD	0.01	(-0.24, 0.26)	
	Total score	5	Secondary	Random	SMD	0.11	(-0.15, 0.37)	
	Physical dimension	2	Secondary	Random	SMD	-0.19	(-0.73, 0.34)	
	Mental dimension	2	Secondary	Random	SMD	-0.04	(-1.17, 1.09)	
	Participant engagement evaluation	5	Secondary	N/A	N/A	N/A	N/A	
	Depressive symptoms (BDI-II, PHQ-9,	8	N/A	N/A	N/A	N/A	N/A	

First Author (Year) [reference number in manuscript]	Narratively or Quantitatively Reported Outcomes [a]	No. of Studies	Definition a Primary or Secondary Outcome	If Meta-Analysis was Conducted				Author's Conclusion
				Random/Fixed Effect Model	Effect Measure	Effect Estimate	(95% CI)	
Hernández-Gómez (2022) [61]	HAM-D6 and HDRS-17)							No improvement in health-related outcomes
	Rate of and accumulated duration of psychiatric admissions	1	N/A	N/A	N/A	N/A	N/A	
	Quality of life (QOLI and WHO-QOL-BREF)	5	N/A	N/A	N/A	N/A	N/A	
	Perceived psychological stress (PSS-10)	2	N/A	N/A	N/A	N/A	N/A	
	Unspecified psychological distress (K-10)	1	N/A	N/A	N/A	N/A	N/A	
	Anxiety levels (STAI-X2, GAD-7, and BAI)	4	N/A	N/A	N/A	N/A	N/A	
	Medication side effects	1	N/A	N/A	N/A	N/A	N/A	
	Internalization of mindfulness skills (FFMQ-SF) and resilience (Resilience Scale)	1	N/A	N/A	N/A	N/A	N/A	
	Dysfunctional attitudes (DAS)	1	N/A	N/A	N/A	N/A	N/A	
	Sleep disturbances (ISI)	1	N/A	N/A	N/A	N/A	N/A	
	Experiential avoidance (AQQ-II)	2	N/A	N/A	N/A	N/A	N/A	
	Self-esteem (RSES)	1	N/A	N/A	N/A	N/A	N/A	

First Author (Year) [reference number in manuscript]	Narratively or Quantitatively Reported Outcomes [a]	No. of Studies	Definition a Primary or Secondary Outcome	If Meta-Analysis was Conducted				Author's Conclusion
				Random/Fixed Effect Model	Effect Measure	Effect Estimate	(95% CI)	
	Adherence to medication (MARS)	1	N/A	N/A	N/A	N/A	N/A	
	Wellbeing (WHO-5)	1	N/A	N/A	N/A	N/A	N/A	
	Rumination (RRS)	1	N/A	N/A	N/A	N/A	N/A	
	Worrying (PSWQ)	1	N/A	N/A	N/A	N/A	N/A	
	Recovery (RAS)	3	N/A	N/A	N/A	N/A	N/A	
	Empowerment (Roger's Empowerment Scale)	1	N/A	N/A	N/A	N/A	N/A	
	Psychosocial functioning (FAST)	1	N/A	N/A	N/A	N/A	N/A	
	Behavioural activation (BADS)	1	N/A	N/A	N/A	N/A	N/A	
Hou (2016) [24]	HbA1c T1DM (%)	4	Primary	Random	MD	-0.36	(-0.87, 0.14)	Better health-related outcomes
	HbA1c T2DM (%)	10	Primary	Random	MD	-0.49	(-0.68, -0.3)	
Hou (2018) [25]	HbA1c T1DM (%)	7	primary	Random	MD	-0.49	(-0.94, -0.04)	Better health-related outcomes
	HbA1c T2DM (%)	16	Primary	Random	MD	-0.57	(-0.82, -0.32)	
Hou (2022) [62]	Severity of symptoms (ICIQ-UI-SF)	6	N/A	N/A	N/A	N/A	N/A	Slightly better health-related outcomes
	Severity of symptoms (ICIQ-VS)	1	N/A	N/A	N/A	N/A	N/A	
	Severity of symptoms (QUID)	1	N/A	N/A	N/A	N/A	N/A	
	QoL (ICIQ-LUTS QoL)	3	N/A	N/A	N/A	N/A	N/A	
	QoL (ICIQ-VS QoL)	1	N/A	N/A	N/A	N/A	N/A	
	QoL (EQ5D-VAS)	1	N/A	N/A	N/A	N/A	N/A	
	Adherence (MARS)	2	NA	NA	NA	NA	NA	
	Self-reported Adherence	1	NA	NA	NA	NA	NA	

First Author (Year) [reference number in manuscript]	Narratively or Quantitatively Reported Outcomes [a]	No. of Studies	Definition a Primary or Secondary Outcome	If Meta-Analysis was Conducted				Author's Conclusion
				Random/Fixed Effect Model	Effect Measure	Effect Estimate	(95% CI)	
Hrynyschyn (2021) [53]	Depressive symptoms	8	Primary	N/A	N/A	N/A	N/A	No improvement in health-related outcomes
	Anxiety	4	Primary	N/A	N/A	N/A	N/A	
	Self-efficacy or self-esteem	4	Primary	N/A	N/A	N/A	N/A	
	QoL	2	Primary	N/A	N/A	N/A	N/A	
Hyun (2021) [26]	HbA1c (%) (3 months) - app + e-coach	4	Subgroup for primary	random	MD	-0.22	(-0,47, 0,02)	Better health-related outcomes
	HbA1c (%) (3 months) - app	4	Subgroup for primary	random	MD	0.06	(-0.34, 0.45)	
	HbA1c (%) (6 months) - app + e-coach	6	Subgroup for primary	random	MD	-0.14	(-0.41, 0.13)	
	HbA1c (%) (6 months) - app	6	Subgroup for primary	random	MD	-0.6	(-1.04, -0.16)	
	HbA1c (%) (12 months) - app + e-coach	3	Subgroup for primary	random	MD	-0.44	(-1.06, 0.18)	
	HbA1c (%) (12 months) - app	3	Subgroup for primary	random	MD	-0.38	(-1.36, 0.61)	
	FBS (3 months) - app + e-coach	3	Subgroup for primary	random	MD	-0.89	(-1.88, 0.1)	
	FBS (6 months) - app + e-coach	2	Subgroup for primary	random	MD	-0.72	(-0.99, -0.44)	
	Hypoglycemia frequency (6 months)	3	Subgroup for primary	random	MD	0.86	(0.5, 1.47)	
Karatas (2022) [54]	Frequency of asthma attacks	1	Primary	N/A	N/A	N/A	N/A	Clinically meaningful and statistically relevant reductions in
	Respiratory infection and antibiotic use days	1	Primary	N/A	N/A	N/A	N/A	
	Medication adherence	2	Primary	N/A	N/A	N/A	N/A	

First Author (Year) [reference number in manuscript]	Narratively or Quantitatively Reported Outcomes [a]	No. of Studies	Definition a Primary or Secondary Outcome	If Meta-Analysis was Conducted				Author's Conclusion
				Random/Fixed Effect Model	Effect Measure	Effect Estimate	(95% CI)	
	Mean peak oxygen uptake	1	Primary	N/A	N/A	N/A	N/A	chronic disease management outcomes, but it is not possible to make definitive conclusions
	HbA1c	1	Primary	N/A	N/A	N/A	N/A	
	BMI	2	Primary	N/A	N/A	N/A	N/A	
	ADHD: pill counts and clinician rating scores	1	Primary	N/A	N/A	N/A	N/A	
	Health behaviour changes: consumption of sugary drinks, television use, and computer time	3	Primary	N/A	N/A	N/A	N/A	
	QoL	4	Primary	N/A	N/A	N/A	N/A	
	Psychosocial outcomes	1	Primary	N/A	N/A	N/A	N/A	
Kassavou (2022) [27]	SBP (mm Hg)	13	Primary	Random	MD	-1.64	(-2.73, -0.55)	Slightly better health-related outcomes
	SBP (mm Hg)	2	Primary	Random	OR	1.6	(0.74, 3.42)	
	DBP (mm Hg)	8	Primary	Random	MD	-0.39	(-2.01, 1.23)	
	DBP (mm Hg)	2	Primary	Random	OR	1.41	N/A	
	Medication adherence (days of adherence/week, summary score to a 5-point scale, 8-item questionnaire)	4	Primary	Random	SMD	0.78	(0.22, 1.34)	
	Medication adherence (days of adherence/week, summary score to a 5-point scale, 8-item questionnaire)	4	primary	Random	OR	3.83	(1.25, 11.76)	

First Author (Year) [reference number in manuscript]	Narratively or Quantitatively Reported Outcomes [a]	No. of Studies	Definition a Primary or Secondary Outcome	If Meta-Analysis was Conducted				Author's Conclusion
				Random/Fixed Effect Model	Effect Measure	Effect Estimate	(95% CI)	
	Physical activity (minutes/day, no. of exercise sessions/week)	4	Primary	Random	SMD	1.63	(-0.35, 3.6)	
	Healthy diet (urinalysis, reducing high sodium consumption)	4	Primary	Random	SMD	-0.44	(-0.79, -0.08)	
	Smoking and alcohol	1	Primary	Random	OR	1.53	(0.76, 3.09)	
Kim (2022) [28]	Depression symptoms (DASS, BDI-II, MADRS, HAM-D, HDRS-17, CDS)	13	Primary	Random	SMD	-0.255	(-0.37, -0.141)	Better health-related outcomes
	Mania symptoms (YMRS)	4	primary	Random	SMD	-0.279	(-0.456, -0.102)	
	Positive psychotic symptoms (PSYRATS, BPRS, PANSS)	5	Primary	Random	SMD	-0.205	(-0.388, -0.022)	
	Negative psychotic symptoms (PSYRATS, BPRS, PANSS)	2	Primary	Random	SMD	-0.406	(-0.791, -0.02)	
	Adverse event	2	Primary	Random	OR	0.779	(0.547, 1.111)	
Lee (2018) [55]	Fatigue	1	Primary	N/A	N/A	N/A	N/A	Better health-related outcomes
	Hand-foot syndrome	1	Primary	N/A	N/A	N/A	N/A	
	Pain management	1	Primary	N/A	N/A	N/A	N/A	
	QoL	2	Primary	N/A	N/A	N/A	N/A	
	Self-care and self-monitoring	2	Primary	N/A	N/A	N/A	N/A	

First Author (Year) [reference number in manuscript]	Narratively or Quantitatively Reported Outcomes [a]	No. of Studies	Definition a Primary or Secondary Outcome	If Meta-Analysis was Conducted				Author's Conclusion
				Random/Fixed Effect Model	Effect Measure	Effect Estimate	(95% CI)	
	HF-related hospital days	2	Primary	N/A	N/A	N/A	N/A	
	Balance in gait	1	Primary	N/A	N/A	N/A	N/A	
	Physical activity	1	Primary	N/A	N/A	N/A	N/A	
	Medication adherence	2	Primary	N/A	N/A	N/A	N/A	
	Caregiver communication	1	Primary	N/A	N/A	N/A	N/A	
	Reporting abnormal health indicators	1	Primary	N/A	N/A	N/A	N/A	
	Parkinson's disease functional scale	1	Primary	N/A	N/A	N/A	N/A	
Leme Nagib (2020) [56]	Urinary incontinence (ICIQ-UI SF)	3	Primary	N/A	N/A	N/A	N/A	Better health-related outcomes
	Urinary incontinence (ICIQ-VS)	1	Primary	N/A	N/A	N/A	N/A	
	Urinary incontinence (QUID)	1	Primary	N/A	N/A	N/A	N/A	
	Quality of life (ICIQ-LUTSQoL)	2	Secondary	N/A	N/A	N/A	N/A	
	Quality of life (ICIQ-VS-QoL)	1	Secondary	N/A	N/A	N/A	N/A	
	Impression of improvement (PGI-I)	2	Secondary	N/A	N/A	N/A	N/A	
	Quality of life (EQ5D-VAS)	1	Secondary	N/A	N/A	N/A	N/A	
	Treatment adherence	2	Secondary	N/A	N/A	N/A	N/A	
Liu (2020) [29]	HbA1c (%)	21	Primary	Random	SMD	-0.44	(-0.59, -0.29)	Better health-related outcomes
	SBP (mm Hg)	16	Primary	Random	SMD	-0.17	(-0.31, -0.03)	
	DBP (mm Hg)	14	Primary	Random	SMD	-0.17	(-0.30, -0.03)	
	FBG	6	Secondary	Random	SMD	−0.29	(-0.49, -0.10)	

First Author (Year) [reference number in manuscript]	Narratively or Quantitatively Reported Outcomes [a]	No. of Studies	Definition a Primary or Secondary Outcome	If Meta-Analysis was Conducted				Author's Conclusion
				Random/Fixed Effect Model	Effect Measure	Effect Estimate	(95% CI)	
	Waist circumference (cm)	4	Secondary	Random	SMD	−0.23	(-0.43, -0.04)	
	Body weight	9	Secondary	Random	SMD	−0.09	(-0.24, 0.07)	
	BMI	6	Secondary	Random	SMD	−0.06	(-0.23, 0.12)	
	TC	7	Secondary	Random	SMD	−0.18	(-0.37, 0.02)	
	LDL-c	7	Secondary	Random	SMD	−0.08	(-0.23, 0.07)	
	HDL-c	7	Secondary	Random	SMD	−0.10	(-0.28, 0.07)	
	TG	7	Secondary	Random	SMD	−0.13	(-0.29, 0.02)	
Lu (2022) [43]	Anxiety symptom management	15	Primary	Random	Hedge's g	-0.1	(-0.14, -0.05)	No improvement in health-related outcomes
	Anxiety symptom management	15	Primary	Fixed	Hedge's g	-0.1	(-0.23, 0.03)	
	Depressive symptom management	15	Primary	Random	Hedge's g	-0.08	(-0.23, 0.07)	
	Depressive symptom management	15	Primary	Fixed	Hedge's g	-0.06	(-0.18, 0.06)	
Lunde (2018) [30]	HbA1c (short-term) (%)	3	Primary	Random	MD	-0.5	(-0.91, -0.08)	Slightly better health-related outcomes
	HbA1c (long-term) (%)	4	primary	Fixed	MD	-0.24	(-0.43, -0.06)	
Marcano Belisario (2013) [57]	Asthma symptom scores (ACQ)	1	Primary	Fixed	MD	0.01	(-0.23, 0.25)	No improvement in health-related outcomes
	Unscheduled visits to emergency department	2	Primary	Fixed	OR	Study 1: 0.2; Study 2: 7.2	Study 1: (0.04, 0.99); Study 2: (0.37, 140.76)	
	Hospital admission	2	Primary	Fixed	OR	Study 1: 0.35; Study 2: 3.07	Study 1: (0.01, 8.79); Study 2: (0.32, 29.83)	

First Author (Year) [reference number in manuscript]	Narratively or Quantitatively Reported Outcomes [a]	No. of Studies	Definition a Primary or Secondary Outcome	If Meta-Analysis was Conducted				Author's Conclusion
				Random/Fixed Effect Model	Effect Measure	Effect Estimate	(95% CI)	
	GP consultations for asthma	1	Primary	Fixed	OR	1.4	(0.85, 2.31)	
	Unscheduled general practice nurse consultation	1	Primary	Fixed	OR	0.6	(0.37, 0.89)	
	Out of hours attendances	1	Primary	Fixed	OR	0.6	(0.14, 2.54)	
	HRQoL (SF-12)	1	Primary	N/A	N/A	N/A	N/A	
	HRQoL (mini-AQLQ)	1	Primary	Fixed	MD	0.02	(-0.35, 0.39)	
	Adherence to intervention	2	Secondary	N/A	N/A	N/A	N/A	
	Lung function - PEFR	1	Secondary	N/A	N/A	N/A	N/A	
	Lung function - FEV1	1	Secondary	N/A	N/A	N/A	N/A	
	Other adverse event - At least one acute asthma exacerbation	1	Secondary	Fixed	OR	0.95	(0.57, 1.57)	
	Other adverse event - Proportion of participants who required at least one course of steroids	1	Secondary	Fixed	OR	0.93	(0.52, 1.65)	
Mikulski (2021) [31]	Medication adherence (MMAS, MASES, % of perscribed doses)	9	Primary	Random	SMD	0.41	(0.02, 0.79)	Better health-related outcomes
Moon (2019) [32]	Visual reception (MSEL)	2	Primary	Random	SMD	0.41	(0.03, 0.80)	Slightly better health-related outcomes
	Fine motor (MSEL)	2	Primary	Random	SMD	0.44	(0.06, 0.81)	
	Receptive language (MSEL)	2	Primary	Random	SMD	0.24	(-0.13, 0.61)	
	Fine expressive (MSEL)	2	Primary	Random	SMD	0.25	(-0,36, 0,86)	

First Author (Year) [reference number in manuscript]	Narratively or Quantitatively Reported Outcomes [a]	No. of Studies	Definition a Primary or Secondary Outcome	If Meta-Analysis was Conducted				Author's Conclusion
				Random/Fixed Effect Model	Effect Measure	Effect Estimate	(95% CI)	
	Word produced (6 months) (MCDI)	2	Primary	Random	SMD	-0.23	(-0.68, 0.22)	
	Gestures (6 months) (MCDI)	2	Primary	Random	SMD	0.32	(-0.05, 0.69)	
	Social communication (3 months) (CSBS)	2	Primary	Random	SMD	0.18	(-0.20, 0.56)	
	Speech (3 months) (CSBS)	2	Primary	Random	SMD	-0.16	(-0.54, 0.22)	
	Symbolic (3 months) (CSBS)	2	Primary	Random	SMD	0.05	(-0.33, 0.43)	
	Social communication (6 months) (CSBS)	2	Primary	Random	SMD	0	(-0.55, 0.55)	
Moreno-Ligero (2023a) [44]	Gait spatiotemporal parameters (speed)	5	Primary	Fixed	MD	0.1	(0.07, 0.13)	Slightly better health-related outcomes
	Gait spatiotemporal parameters (cadence)	2	Primary	Fixed	MD	8.01	(3.3, 12.72)	
	Gait spatiotemporal parameters (stride length)	2	Primary	Fixed	MD	0.08	(-0.00, 0.16)	
	Gait spatiotemporal parameters (affected step length)	2	Primary	Fixed	MD	8.89	(4.88, 12.9)	
	Gait spatiotemporal parameters (non-affected step length)	2	Primary	Fixed	MD	8.08	(2.64, 13.51)	
	Dynamic balance (TUG)	4	Primary	Fixed	MD	-7.15	(-9.3, -4.99)	
	Dynamic balance (PASS (mobility))	2	Primary	Fixed	MD	1.71	(1.39, 2.04)	
	Dynamic balance (FSST)	2	Primary	Fixed	MD	-1.9	(-3.82, 0.03)	

First Author (Year) [reference number in manuscript]	Narratively or Quantitatively Reported Outcomes [a]	No. of Studies	Definition a Primary or Secondary Outcome	If Meta-Analysis was Conducted				Author's Conclusion
				Random/Fixed Effect Model	Effect Measure	Effect Estimate	(95% CI)	
	Dynamic balance (6MWT)	5	Primary	Fixed	MD	11.74	(-16.4, 39.89)	
	Dynamic balance (2MWT)	2	Primary	Fixed	MD	5.13	(-10.55, 20.81)	
Moreno-Ligero (2023b) [60]	Pain intensity (NRS, VAS, KOOS, HOOS, WOMAC)	17	Primary	N/A	N/A	N/A	N/A	Slightly better health-related outcomes
	QoL (SF-36, EuroQoL-5D)	15	Primary	N/A	N/A	N/A	N/A	
	Functional disability (NDI, FIQ, KOOS, HOOS, WOMAC)	17	Primary	N/A	N/A	N/A	N/A	
Özden (2023a) [45]	Disease/symptom severity (UPDRS III)	2	N/A	Random	SMD	0.86	(-0.94, 2.46)	Slightly better health-related outcomes
	Drug adherence	N/A	N/A	N/A	N/A	N/A	N/A	
	Quality of life (SF-36, EQ-5D-5 L)	N/A	N/A	N/A	N/A	N/A	N/A	
	Aerobic capacity	2	NA	NA	NA	NA	NA	
	Function (balance and gait kinematics) (MiniBESTest)	2	N/A	Random	SMD	0.15	(-0.33, 0.26)	
	Cognition and psychology (MOCA, GDS, CTT)	N/A	N/A	N/A	N/A	N/A	N/A	
Özden (2023b) [46]	Function (KOOS)	2	N/A	Random	SMD	0.23	(0.11, 1.02)	Better health-related outcomes
	Pain (VAS)	N/A	N/A	N/A	N/A	N/A	N/A	

First Author (Year) [reference number in manuscript]	Narratively or Quantitatively Reported Outcomes [a]	No. of Studies	Definition a Primary or Secondary Outcome	If Meta-Analysis was Conducted				Author's Conclusion
				Random/Fixed Effect Model	Effect Measure	Effect Estimate	(95% CI)	
	QoL (EQ-5D-5L)	N/A	N/A	N/A	N/A	N/A	N/A	
	Range of motion (active ROM)	2	N/A	Random	SMD	0.28	(-0.31, 0.79)	
Park (2020) [33]	Depressive symptoms (PHQ-9, MADRS, HDRS, BDI-II, DASS, MDI, CES-D)	22	Primary	Random	Hedge's g	-0.212	(-0.351, -0.073)	Slightly better health-related outcomes
Peng (2020) [34]	Medication adherence (self-report, pill count, medication event monitoring system)	14	Primary	Random	SMD	0.4	(0.27, 0.52)	Better health-related outcomes
Pi (2023) [47]	HbA1c (%)	9	Primary	Random	WMD	-0.26	(-0.56, 0.05)	No improvement in health-related outcomes
Seegan (2023) [49]	Anxiety symptoms severity (DASS-A, GAD, HADS-A, BAI)	28	Primary	Random	MD (Hedge's g)	0.306	(0.22, 0.393)	Better health-related outcomes
	Depression symptoms severity (BAI, DASS-D, BDI, PHQ-9, CES-D, HADS-D)	39	Primary	Random	MD (Hedge's g)	0.35	(0.26, 0.44)	
Shaw (2020) [35]	Frequency of COPD exacerbations	5	Primary	N/A	N/A	N/A	N/A	No improvement in health-related outcomes
	Physial function (incremental shuttlewalking test, 6-minute walk test)	4	Secondary	Random	MD	8.38	(-4.4, 21.17)	

First Author (Year) [reference number in manuscript]	Narratively or Quantitatively Reported Outcomes [a]	No. of Studies	Definition a Primary or Secondary Outcome	If Meta-Analysis was Conducted				Author's Conclusion
				Random/Fixed Effect Model	Effect Measure	Effect Estimate	(95% CI)	
	QoL (SF-12, SF-36, CCQ, Chronic Respiratory Disease Questionnaire, St. George's Respiratory Questionnaire, COPD Assessment Test)	8	Secondary	Random	SMD	-0.4	(-0,86, 0.05)	
	Dyspnea	5	Secondary	N/A	N/A	N/A	N/A	
	Fatigue	5	Secondary	N/A	N/A	N/A	N/A	
	Physical activity	5	Secondary	N/A	N/A	N/A	N/A	
	Self-efficacy	4	Secondary	N/A	N/A	N/A	N/A	
	Anxiety and depression	2	Secondary	N/A	N/A	N/A	N/A	
Thompson (2023) [48]	Pain intensity (VAS, NPRS, WOMAC, KOOS-Pain)	9	N/A	Random	SMD	-0.6	(-0.93, -0.27)	Slightly better health-related outcomes
	Pain interference (NDI, MODI)	3	N/A	Random	SMD	–0.66	(-1.52, 0.19)	
	Self-reported physical function (WOMAC, KOOS, FADI)	5	N/A	Random	SMD	-0.92	(-1.57, -0.27)	
	Physical performance	6	N/A	N/A	N/A	N/A	N/A	
	Adherence	3	N/A	N/A	N/A	N/A	N/A	
	Psychosocial outcomes	2	N/A	N/A	N/A	N/A	N/A	
	QoL (SF-36)	2	N/A	N/A	N/A	N/A	N/A	
Whitehead (2016) [58]	HbA1c T1DM	2	Primary	N/A	N/A	N/A	N/A	Slightly better health-related outcomes
	HbA1c T2DM	3	Primary	N/A	N/A	N/A	N/A	
	6MWT	1	Primary	N/A	N/A	N/A	N/A	

First Author (Year) [reference number in manuscript]	Narratively or Quantitatively Reported Outcomes [a]	No. of Studies	Definition a Primary or Secondary Outcome	If Meta-Analysis was Conducted				Author's Conclusion
				Random/Fixed Effect Model	Effect Measure	Effect Estimate	(95% CI)	
	Lung function parameters	3		N/A	N/A	N/A	N/A	
Wickersham (2019) [59]	PTSD symptoms	5	Primary	N/A	N/A	N/A	N/A	Slightly better health-related outcomes
	Stress, anxiety, depression, self-compassion, sleep quality, pain, psychosocial functioning, and quality of life.	4	Secondary	N/A	N/A	N/A	N/A	
Wu (2017) [37]	HbA1c (%)	12	Primary	Random	MD	-0.48	(-0.19, -0.78)	Better health-related outcomes
	Adverse events	5	N/A	N/A	N/A	N/A	N/A	
Wu (2019) [36]	HbA1c T1DM pooled	5	Primary	Random	MD	-0.21	(-0.52, 0.09)	Better health-related outcomes
	HbA1c T2DM pooled	11	Primary	Fixed	MD	-0.35	(-0.48, -0.21)	
	HbA1c prediabetes total	2	Primary	Random	MD	-0.03	(-0.17, 0.11)	
Xu (2020) [38]	SBP (mm Hg)	6	Primary	Random	WMD	-2.28	(-3.90, -0.66)	Better health-related outcomes
	DBP (mm Hg)	5	Primary	Random	WMD	-1.84	(-3.49, -9.19)	
	Medication adherence (MMAS, pill count, Hypertension SM Behavior Questionnaire)	4	Primary	Random	SMD	0.38	(0.26, 0.50)	
	Physical activity	2	Primary	Random	SMD	0.13	(-0.11, 0.37)	
Yang (2018) [39]	Hospital admission	6	Primary	Random	RR	0.73	(0,52, 1,04)	Slightly better health-related outcomes
	Average hospital stay (days)	6	Primary	Random	SMD	-0.06	(-0.31, 0.18)	
	Exercise capacity and acitivity levels	5	Secondary	N/A	N/A	N/A	N/A	
	Lung function - FEV1	2	Secondary	N/A	N/A	N/A	N/A	

First Author (Year) [reference number in manuscript]	Narratively or Quantitatively Reported Outcomes [a]	No. of Studies	Definition a Primary or Secondary Outcome	If Meta-Analysis was Conducted				Author's Conclusion
				Random/Fixed Effect Model	Effect Measure	Effect Estimate	(95% CI)	
Legend: [a] If there were no pooled effects of the main outcome, then the subgroup analysis were included here as a main outcome<br><br>2MWT: 2-minute Walk Test; 6MWT: 6-minute Walk Test; ACQ: Asthma Control Questionnaire; ADHD: Attention deficit hyperactivity syndrome; AQLQ: Asthma Quality of Life Questionnaire; AQQ-II: Acceptance and Action Questionnaire; BADS: Behavioral Activation for Depression Scale; BAI: Beck Anxiety Inventory; BDI: Beck Depression Inventory; BDI-II: Beck Depression Inventory; BMI: Body mass index; BP: Blood pressure; BPRS: Brief Psychiatric Rating Scale; CCQ: Chronic COPD Questionnaire; CDS: Calgary Depression Scale; CES-D: Center for Epidemiological Studies Depression Scale; COPD: Chronic obstructive pulmonary disease; CSBS: Communication and Symbolic Behaviour Scales; CTT: color trail test; CVD: Cardiovascular; DAS: Dysfunctional Attitude Scale; DASS: Depression Anxiety Stress Scale; DBP: Diastolic blood pressure; DM: Diabetes mellitus; EQ5D-VAS: EuroQol 5D-Visual Analog Scale; FADI: Foot and Ankle Disability Index; FAST: Functional Assessment Short Test; FBG: Fasting blood glucose; FBS: Fasting Blood Sugar; FEV: forced expiratory volume; FFMQ-SF: Five Facet Mindfulness Questionnaire Short Form; FIQ: Fibromyalgia Impact Questionnaire; FSST: Four Squared Step Test; GAD-7: Generalized Anxiety Disorder 7-item; GDS: Global deterioration scale; HADS-A: Hospital and anxiety depression scale - anxiety; HADS-D: Hospital and anxiety depression scale - depression; HAM-D: Hamilton Depression Rating; HAM-D6: Hamilton Depression Scale 6 items; HbA1c: Glycated haemoglobin; HDL-C: High density lipoprotein; HDRS: Hamilton Depression Rating Scale; HOOS: Hip injury and Osteoarthritis Outcome Score; ICIQ-UI SF: International Consultation on Incontinence Modular Questionnaire Urinary Incontinence Short Form; ICIQ-VS: International Consultation on Incontinence Questionnaire- Vaginal Symptoms; ICIQ-LUTSQoL: ICIQ Lower Urinary Tract Symptoms QoL; ICIQ-VS-QoL: International Consultation on Incontinence Questionnaire - Vaginal Symptoms - Quality of Life; ISI: Insomnia Severity Index; K-10: Kessler 10-item Psychological Distress Scale; KOOS: Knee injury and Osteoarthritis Outcome Score; LDL-C: Low density lipoprotein-cholesterol; MADRS: Montgomery-Asberg Depression Rating Scale; MARS: Medicine Adherence Rating Scale; MCDI: MacCarthur-Bates Communication Development Inventory; MD: Mean difference; MDI: Major Depression Inventory; MMAS: Morisky Medication Adherence Scale; MOCA: Montreal Cognitive Assessment; MODI: Modified Oswestry Disability Index; MSEL: Mullen Scales of Early Learning; NDI: Neck Disability Index; NPRS: Numerical Pain Rating Scale; NRS: numeric rating scale; PAM: Patient Activation Measures; PANSS: Positive and Negative Syndrome Scale; PASS: Postural Assessment Scale for Stroke; PEFR: peak expiratory flow rate; PGI–I: Patient's Global Impression of Improvement; PHQ-9: Patient-Health Questionnaire 9; PSS-10: Perceived Stress Scale; PSWQ: Penn State Worry Questionnaire; PTSD: Post-traumatic stress disorder; QoL: Quality of life; QOLI: Quality of Life Inventory; QUID: Questionnaire for Urinary Incontinence Diagnosis; RAS: Recovery Assessment Scale; ROM: range of motion; RRS: Ruminative Response Scale; RSES: Rosenberg Self-Esteem Scale; SBP: Systolic blood pressure; SF-36: Short Form 36 quality of life questionnaire; SMD: Standardized mean difference; STAI-X2: State-Trait Anxiety Inventory; T1DM: Type 1 diabetes mellitus; T2DM: Type 2 diabetes mellitus; TC: Total cholesterol; TG: Triglycerides; TUG: Timed Up and Go; UPDRS: The unified Parkinson's disease rating scale; VAS: visual analogue scale; WHO-5: The World Health Organization-Five Wellbeing Index; WHO-QOL-BREF: WHO Quality of Life-BREF; WOMAC: Western Ontario and McMaster; YMRS: Young Manic Rating Scale								

Supplementary Table 7. Summary of Subgroup Analyses

First Author (Year) [reference number in manuscript]	Outcomes Measured [a]	Subgroup [b]
Bonoto (2017) [18]	HbA1c	With 1, 2, 3, or 4 features; remote or usual care access
Cai (2020) [19]	Body weight	BMI >30, BMI <30; ethnicity; standalone and complementary; with and without exercise, diet, weight, glucose monitoring/recording
Chew (2022) [41]	Weight loss	Type of control condition
Chew (2023) [40]	Weight loss	Type of control conditions; number of coaching sessions
Cui (2016) [20]	HbA1c	Feedback; methodology; HbA1c at baseline < and >8%
El-Gayar (2021) [21]	HbA1c	T1/2DM; trial duration (<=3 mo, >3 - <=9 mo, >9mo)
He (2021) [23]	HbA1c	Duration of intervention; format of delivery; frequency of facilitator-patient interaction; user training; baseline of HbA1c < or >=9%
Hou (2016) [24]	HbA1c T2DM	Follow-up duration; age of participants; no. of self-monitoring tasks; with/without HCP feedback; quality of studies
Hou (2018) [25]	HbA1c T1DM	With/without HCP feedback
	HbA1c T2DM	With no, low, and high frequency HCP feedback
Hrynyschyn (2021) [53]	Depressive symptoms	Mild depression; baseline depression
Hyun (2021) [26]	HbA1c	Homogenous/heterogenous gender ratio
	FBG	Homogenous/heterogenous gender ratio
Kassavou (2022) [27]	SBP	Tailored and nontailored intervention
	DBP	Tailored and nontailored intervention
Kim (2022) [28]	Depression symptoms	Applications with feedback, notifications, use of data tracking, and additional strategies
	Mania symptoms	Applications with feedback, notifications, and additional strategies
	Positive psychotic symptoms	Applications with feedback, use of data tracking, and additional strategies
Liu (2020) [29]	SBP	By disease type (DM/HTN); intervention feature
	DBP	By disease type (DM/HTN); intervention feature
Lu (2022) [43]	Anxiety and depression symptom management	Depression vs anxiety
Marcano Belisario (2013) [57]	HRQoL (SF-12)	Meta-analysis of subgroups at 3,4,5,6 months of follow-up
	Adherence to intervention	Meta-analysis of subgroups at 3,4,5,6 months of follow-up

	Lung function - PEFR	Meta-analysis of subgroups at 1,2,3,4,5,6 months
	Lung function - FEV1	Meta-analysis of subgroups at 3 and 6 months
Park (2020) [33]	Depressive symptoms	Comparing smartphone intervention with active conditions/with inactive conditions; depressive symptoms with bipolar disorder/with suicide risk/with MDD/with other specified depression/with self-reported depression
Peng (2020) [34]	Medication adherence	Sample size; mean age; region; type of chronic disease; intervention duration; type of outcome assessing method; medication reminder; medication education; clinical decision support; 2-way communication; intervention components
Pi (2023) [47]	HbA1c	Auxiliary-style apps vs recording-style apps; intervention duration
Seegan (2023) [49]	Anxiety and depression symptom severity	Demographics, trial design and characteristics, DMHA characteristics
Thompson (2023) [48]	Pain intensity	Type of disease
	Pain interference	
	Self-reported physical function	
	Physical performance	
	Psychosocial outcomes	
	QoL	
Wu (2017) [37]	HbA1c	T1/2DM; modules, risks, and technologies
	Adverse events	
Wu (2019) [36]	HbA1c T1DM	Short- and long-term effects
	HbA1c T2DM	Short- and long-term effects

Legend: [a] If there were no pooled effects reported for the main outcome across all participants, then a subgroup is additionally indicated in this column.
[b] Subgroups as indicated by the respective authors for additional analyses

BMI: body mass index; DBP: diastolic blood pressure; FBG: fasting blood glucose; FEV: forced expiratory volume; HbA1c: glycated haemoglobin; HCP: health care professional; HRQoL: health-related quality of life; HTN: hypertension; MDD: major depressive disorder; SBP: systolic blood pressure; T1DM: type 1 diabetes mellitus; T2DM: type 2 diabetes mellitus; PAM: Patient Activation Measures; PEFR: peak expiratory flow rate; QoL: quality of life.

Supplementary Table 8. PRISMA Checklist

Section and Topic	Item #	Checklist item	Location where item is reported
**TITLE**			
Title	1	Identify the report as a systematic review.	Page 1
**ABSTRACT**			
Abstract	2	See the PRISMA 2020 for Abstracts checklist.	Page 2
**INTRODUCTION**			
Rationale	3	Describe the rationale for the review in the context of existing knowledge.	Page 4-5
Objectives	4	Provide an explicit statement of the objective(s) or question(s) the review addresses.	Page 5-6
**METHODS**			
Eligibility criteria	5	Specify the inclusion and exclusion criteria for the review and how studies were grouped for the syntheses.	Page 19-21,23
Information sources	6	Specify all databases, registers, websites, organisations, reference lists and other sources searched or consulted to identify studies. Specify the date when each source was last searched or consulted.	Page 21
Search strategy	7	Present the full search strategies for all databases, registers and websites, including any filters and limits used.	Suppl.Tab.8,9
Selection process	8	Specify the methods used to decide whether a study met the inclusion criteria of the review, including how many reviewers screened each record and each report retrieved, whether they worked independently, and if applicable, details of automation tools used in the process.	Page 21,22
Data collection process	9	Specify the methods used to collect data from reports, including how many reviewers collected data from each report, whether they worked independently, any processes for obtaining or confirming data from study investigators, and if applicable, details of automation tools used in the process.	Page 22
Data items	10a	List and define all outcomes for which data were sought. Specify whether all results that were compatible with each outcome domain in each study were sought (e.g. for all measures, time points, analyses), and if not, the methods used to decide which results to collect.	Page 22
	10b	List and define all other variables for which data were sought (e.g. participant and intervention characteristics, funding sources). Describe any assumptions made about any missing or unclear information.	Page 22

Section and Topic	Item #	Checklist item	Location where item is reported
Study risk of bias assessment	11	Specify the methods used to assess risk of bias in the included studies, including details of the tool(s) used, how many reviewers assessed each study and whether they worked independently, and if applicable, details of automation tools used in the process.	Page 22-23
Effect measures	12	Specify for each outcome the effect measure(s) (e.g. risk ratio, mean difference) used in the synthesis or presentation of results.	Suppl.Tab.5
Synthesis methods	13a	Describe the processes used to decide which studies were eligible for each synthesis (e.g. tabulating the study intervention characteristics and comparing against the planned groups for each synthesis (item #5)).	Page 23,24
	13b	Describe any methods required to prepare the data for presentation or synthesis, such as handling of missing summary statistics, or data conversions.	Not Applicable
	13c	Describe any methods used to tabulate or visually display results of individual studies and syntheses.	Page 23
	13d	Describe any methods used to synthesize results and provide a rationale for the choice(s). If meta-analysis was performed, describe the model(s), method(s) to identify the presence and extent of statistical heterogeneity, and software package(s) used.	Page 23
	13e	Describe any methods used to explore possible causes of heterogeneity among study results (e.g. subgroup analysis, meta-regression).	Not Applicable
	13f	Describe any sensitivity analyses conducted to assess robustness of the synthesized results.	Not Applicable
Reporting bias assessment	14	Describe any methods used to assess risk of bias due to missing results in a synthesis (arising from reporting biases).	Not Applicable
Certainty assessment	15	Describe any methods used to assess certainty (or confidence) in the body of evidence for an outcome.	Not Applicable
**RESULTS**			
Study selection	16a	Describe the results of the search and selection process, from the number of records identified in the search to the number of studies included in the review, ideally using a flow diagram.	Page 6
	16b	Cite studies that might appear to meet the inclusion criteria, but which were excluded, and explain why they were excluded.	Page 6,17,18 Suppl.Tab.1
Study characteristics	17	Cite each included study and present its characteristics.	Page 6-11
Risk of bias in studies	18	Present assessments of risk of bias for each included study.	Page 7, Suppl.Tab.3

Section and Topic	Item #	Checklist item	Location where item is reported
Results of individual studies	19	For all outcomes, present, for each study: (a) summary statistics for each group (where appropriate) and (b) an effect estimate and its precision (e.g. confidence/credible interval), ideally using structured tables or plots.	Suppl.Tab.5
Results of syntheses	20a	For each synthesis, briefly summarise the characteristics and risk of bias among contributing studies.	Page 6-11
	20b	Present results of all statistical syntheses conducted. If meta-analysis was done, present for each the summary estimate and its precision (e.g. confidence/credible interval) and measures of statistical heterogeneity. If comparing groups, describe the direction of the effect.	Not Applicable
	20c	Present results of all investigations of possible causes of heterogeneity among study results.	Not Applicable
	20d	Present results of all sensitivity analyses conducted to assess the robustness of the synthesized results.	Not Applicable
Reporting biases	21	Present assessments of risk of bias due to missing results (arising from reporting biases) for each synthesis assessed.	Not Applicable
Certainty of evidence	22	Present assessments of certainty (or confidence) in the body of evidence for each outcome assessed.	Not Applicable
**DISCUSSION**			
Discussion	23a	Provide a general interpretation of the results in the context of other evidence.	Page 11-16
	23b	Discuss any limitations of the evidence included in the review.	Page 16-18
	23c	Discuss any limitations of the review processes used.	Page16-18
	23d	Discuss implications of the results for practice, policy, and future research.	Page 18
**OTHER INFORMATION**			
Registration and protocol	24a	Provide registration information for the review, including register name and registration number, or state that the review was not registered.	Not Applicable
	24b	Indicate where the review protocol can be accessed, or state that a protocol was not prepared.	Page 19
	24c	Describe and explain any amendments to information provided at registration or in the protocol.	Page 19
Support	25	Describe sources of financial or non-financial support for the review, and the role of the funders or sponsors in the review.	Page 24

Section and Topic	Item #	Checklist item	Location where item is reported
Competing interests	26	Declare any competing interests of review authors.	Page 24
Availability of data, code and other materials	27	Report which of the following are publicly available and where they can be found: template data collection forms; data extracted from included studies; data used for all analyses; analytic code; any other materials used in the review.	Page 24

From: Page MJ, McKenzie JE, Bossuyt PM, Boutron I, Hoffmann TC, Mulrow CD, et al. The PRISMA 2020 statement: an updated guideline for reporting systematic reviews. BMJ 2021;372:n71. doi: 10.1136/bmj.n71

For more information, visit: http://www.prisma-statement.org/

Supplementary Table 9. Search Strategy and Hits in PubMed

Search	Query	Hits up to March 15, 2022	Hits March 15 to August 28, 2023
#1	mobile applications[MeSH Terms]	9,619	11,608
#2	computers, handheld[MeSH Terms]	11,243	13,113
#3	"Medical Informatics Applications"[Mesh:NoExp]	2,549	2,551
#4	mobile health[Title/Abstract] OR mhealth[Title/Abstract] OR m-health[Title/Abstract]	11,137	14,226
#5	mobile application[Title/Abstract] OR mobile applications[Title/Abstract] OR smartphone application[Title/Abstract] OR smartphone applications[Title/Abstract] OR smart-phone application[Title/Abstract] OR smart-phone applications[Title/Abstract] OR mobile-phone application[Title/Abstract] OR mobile-phone applications[Title/Abstract] OR mobilephone application[Title/Abstract] OR mobilephone applications[Title/Abstract]	7,611	10,044
#6	**(app[Title/Abstract] NOT amyloid[Title/Abstract])**	21,441	27,326
#7	**(apps[Title/Abstract] NOT amyloid[Title/Abstract])**	8,680	11,294
#8	smart-phone based[Title/Abstract] OR smartphone based[Title/Abstract] OR mobile-phone based[Title/Abstract] OR mobilephone based[Title/Abstract] OR mobile	5,003	6,596

	based[Title/Abstract] OR application based[Title/Abstract]		
#9	#1 OR #2 OR #3 OR #4 OR #5 OR #6 OR #7 OR #8	50,142	62,214
#10	systematic review[Title] OR meta-analysis[Title]	246,247	305,3016
#11	"Systematic Review" [Publication Type]	188,027	235,972
#12	"Meta-Analysis" [Publication Type]	154,817	185,481
#13	#10 OR #11 OR #12	320,900	391,159
#14	efficac*[Title/Abstract] OR effect*[Title/Abstract] OR intervention[Title/Abstract] or interventions[Title/Abstract]	8,789,584	9,655,991
#15	#9 AND #13 AND #14	1,354	1,820
#16	#15 Filters: English	1,341	1,803

Supplementary Table 10. Search Strategy in the Cochrane Database of Systematic Reviews

Search	Query	Hits as of March 28, 2023
#1	("mobile applications"):kw in Cochrane Reviews	5
#2	("computers, handheld"):kw in Cochrane Reviews	5
#3	smartphone:kw in Cochrane Reviews	3
#4	("Medical Informatics Applications"):kw in Cochrane Reviews	1
#5	("mobile health"):ab,ti in Cochrane Reviews	3
#6	mhealth:ab,ti in Cochrane Reviews	4
#7	(m-health):ab,ti in Cochrane Reviews	4
#8	(mobile NEAR/1 application?):ti,ab in Cochrane Reviews	3
#9	(smartphone NEAR/1 application?):ti,ab in Cochrane Reviews	8
#10	(smart-phone NEAR/1 application?):ti,ab in Cochrane Reviews	0
#11	(mobilephone NEAR/1 application?):ti,ab in Cochrane Reviews	0
#12	(mobile-phone NEAR/1 application?):ti,ab in Cochrane Reviews	5
#13	(app? not amyloid):ti,ab in Cochrane Reviews	59
#14	("smartphone based"):ti,ab in Cochrane Reviews	0
#15	("smart-phone based"):ti,ab in Cochrane Reviews	0
#16	("mobilephone based"):ti,ab in Cochrane Reviews	0
#17	("mobile-phone based"):ti,ab in Cochrane Reviews	6

#18	("mobile based"):ti,ab in Cochrane Reviews	1
#19	("application based"):ti,ab in Cochrane Reviews	0
#20	efficac*:ti,ab in Cochrane Reviews	4,776
#21	effect*:ti,ab in Cochrane Reviews	8,554
#22	intervention?:ti,ab in Cochrane Reviews	5,176
#23	#1 or #2 or #3 or #4 in Cochrane Reviews	11
#24	#5 or #6 or #7 in Cochrane Reviews	4
#25	#8 or #9 or #10 or #11 or #12	14
#26	#14 or #15 or #16 or #17 or #18 or #19	7
#27	#13 or #23 or #24 or #25 or #26	79
#28	#20 or #21 or #22	8,722
#29	#27 and #28	78

Supplementary Note 1.

Additional Notes on Decision Rules Applied During the AMSTAR2 Rating Process

Item 2: If the systematic review provided a protocol registration number e.g. for PROSPERO, we checked the protocol to see if all relevant items were listed. We accepted search strategies if the protocol comprised both the databases to be searched and the list of seach terms with operators. If a protocol was mentioned but we were unable to find it in a public repository, the item was rated as “No” unless the systematic review stated explicitly that all items required for “Partial Yes” were prespecified in the protocol.

Item 4: If no rationale was provided for language restrictions (e.g. inclusion only if full texts were available in English), we rated this item as “No”.

Item 5: If the systematic review reported that study selection was done by two or more reviewers without explicitly mentioning a consensus process or stating that each record was independently screened by more than one author, we rated these statements as “No” since AMSTAR2 does not provide the possibility to rate an item as “inconclusive” or “ambiguous”. Also, if the systematic review reported that only titles and abstracts or only the full texts were screened in duplicate, we rated this item as “No”.

Item 6: If the systematic review reported only data extraction by two or more reviewers without explicitly mentioning a consensus process or stating that each record was independently extracted by more than one author, we rated these inconclusive statements as “No” since AMSTAR2 does not provide the possibility to rate an item as “inconclusive” or “ambiguous”.
